# Neuronal Subtype-Specific Expression of γ-Enolase: Its Role in Neuronal Differentiation

**DOI:** 10.1007/s12017-025-08902-9

**Published:** 2026-01-30

**Authors:** Selena Horvat, Urša Pečar Fonović, Nace Zidar, Bojan Doljak, Janko Kos, Anja Pišlar

**Affiliations:** 1 Department of Pharmaceutical Biology, Faculty of Pharmacy, University of Ljubljana, Aškerčeva 7, Ljubljana, 1000 Slovenia; 2 Department of Pharmaceutical Chemistry, Faculty of Pharmacy, University of Ljubljana, Aškerčeva 7, Ljubljana, 1000 Slovenia; 3 Department of Biotechnology, Jožef Stefan Institute, Jamova 39, Ljubljana, 1000 Slovenia

**Keywords:** Neuronal specific subtype, Enolase isoforms, γ-Enolase, Neurotrophic activity, Cathepsin x regulation

## Abstract

Neuronal differentiation into specific subtypes is crucial for nervous system development and function, guided by neurotrophic factors. γ-Enolase, a neuron-specific glycolytic enzyme, exhibits neurotrophic-like properties and supports neuronal differentiation; however, its role in specific neuronal subtypes remains unknown. Here, we investigate the role of γ-enolase in differentiation dopaminergic-, cholinergic-, and adrenergic-like neuronal cells. Our results demonstrate that γ-enolase expression is significantly upregulated in differentiated cells, with the highest expression observed in cholinergic-like neurons. Full-length γ-enolase, compared to its truncated form, promoted enhanced neurite outgrowth and increased β-tubulin, a cytoskeletal marker. Conversely, silencing endogenous γ-enolase significantly reduced neurite length, confirming its essential role in driving neuronal morphological maturation. Furthermore, a γ-enolase-derived peptide corresponding to the active C-terminus of γ-enolase significantly promoted neurite outgrowth and increased β-tubulin expression, particularly in cholinergic-like neuronal cells. Notably, γ-enolase activity is regulated by cathepsin X, a lysosomal peptidase that cleaves γ-enolase at its C-terminus, reducing its neurotrophic effects. Confocal microscopy revealed increased co-localization of γ-enolase and cathepsin X in differentiated neuronal cells, emphasizing their interaction in cholinergic-like neurons. Inhibiting cathepsin X preserved active γ-enolase, promoted neuronal differentiation, and altered cytoskeletal marker expression. These findings suggest an important role for γ-enolase in cholinergic-like neuronal cells and propose cathepsin X as a regulatory modulator of γ-enolase activity, suggesting novel therapeutic strategies for neuroregeneration.

**Supplementary Information:**

The online version contains supplementary material available at 10.1007/s12017-025-08902-9.

## Introduction

Enolase is a glycolytic enzyme with different isoforms: α-enolase, which is ubiquitously expressed, and β- and γ-enolase, which are tissue-specific. These isoforms form active dimers with distinct physiological roles (Fletcher et al., 1976; Pancholi, 2001). All enolase isoforms participate in catalyzing the conversion of 2-phosphoglycerate to phosphoenolpyruvate during glycolysis. Nevertheless, γ-enolase has gained attention due to its prominent non-glycolytic roles (reviewed in Horvat et al., 2024). In addition to its metabolic role, γ-enolase has been implicated in neurotrophic support, neurite outgrowth, and neuroprotection in neurodegenerative and neuroinflammatory conditions (Hattori et al., 1995; Hafner et al., 2012, 2013; Fu et al., 2023). Furthermore, γ-enolase is predominantly expressed in mature neurons and neuroendocrine cells and thus serves as an established neuronal marker (Marangos & Schmechel, 1987).

In the developing brain, the transition from αα to γγ dimers occurs in cell types with high energy requirements. This transition is closely associated with the morphological and functional maturation of neurons, underscoring its role in neuronal differentiation and synaptic integration (Schmechel et al., 1980). Similar isoform transitions have been observed in neuronal cultures (Liegro et al., 1991), and elevated γ-enolase levels were detected in differentiating neuroblastoma cells, highlighting the association of γ-enolase with neuronal differentiation (Marangos & Schmechel, 1987). Indeed, γ-enolase promotes neuronal survival and neurite outgrowth by activating the phosphatidylinositol 3-kinase and mitogen-activated protein kinase pathways, resulting in cytoskeleton reorganization and cell remodeling (Hafner et al., 2012). The neurotrophic activity of γ-enolase in neuronal cells is regulated by various proteins. For example, γ1-syntrophin enables the localization of γ-enolase within the plasma membrane (Hattori et al., 1994, 1995; Takei et al., 1991; Hafner et al., 2010). Conversely, cathepsin X, a lysosomal enzyme, cleaves the C-terminus of γ-enolase, preventing its binding to γ1-syntrophin and thus translocation to the plasma membrane (Obermajer et al., 2009). Understanding these regulatory interactions in differentiated neuronal subtypes can provide valuable insights into the specific roles of γ-enolase in neuroprotection and regeneration.

Despite its well-documented importance in general neuronal function, any potential differential roles of γ-enolase in specific neuronal subtypes remain underexplored. Neurons are highly diverse in both function and morphology, performing specialized roles in the central and peripheral nervous systems (Purves, 2004). Cholinergic neurons, which primarily release acetylcholine, are of fundamental importance to processes such as motor control, attention, and memory. Dysregulation within this system is strongly associated with neurodegenerative diseases such as Alzheimer’s disease, in which cholinergic deficits contribute to cognitive impairments (Gasiorowska et al., 2021; Ahmed et al., 2019). Conversely, dopaminergic neurons are essential for functions related to motivation, reward, and motor coordination. The degeneration of dopaminergic neurons in the substantia nigra is a hallmark of Parkinson’s disease, leading to motor symptoms such as tremors and bradykinesia (Harsing, 2008). Furthermore, adrenergic neurons, which release norepinephrine, play a key role in autonomic and stress responses. Dysregulated adrenergic signaling has been implicated in psychiatric disorders such as anxiety, depression, and post-traumatic stress disorder (Amalric et al., 2021). Adrenergic neurons also interact with cholinergic and dopaminergic systems, influencing complex behaviors such as attention, decision-making, and emotional regulation, highlighting the interconnected nature of these neural systems. To date, the expression patterns and functions of γ-enolase across these neuronal subtypes remain underexplored.

Several neuronal cell lines can be differentiated into specific neuronal subtypes (Cetin et al., 2022), which exhibit molecular, structural, and functional properties of mature neurons, making them essential tools for studying cell-specific protein expression and function. The human neuroblastoma cell line SH-SY5Y is the most commonly used cell line in studies of cell differentiation and neurodegeneration. Depending on the culture medium, SH-SY5Y cells can differentiate into several distinct neuronal subtypes (e.g., cholinergic-like, adrenergic-like, and dopaminergic-like) and thus provide a reliable in vitro system for studying neuronal differentiation (Kovalevich & Langford, 2013). Additionally, Neuro-2a is a mouse neuroblastoma cell line that differentiates into dopaminergic neurons (Tremblay et al., 2010), and LA-N-2 is a human neuroblastoma cell line that differentiates into cholinergic neurons and is used to study cholinergic functions (Crosland, 1996).

In this study, we investigated the expression patterns and roles of enolase isoforms, in particular γ-enolase, in neuronal cells differentiated into specific subtypes as well as the regulation of γ-enolase by cathepsin X. First, we established and validated a differentiation model for distinct neuronal subtypes using subtype-specific markers. Our findings reveal significantly increased γ-enolase expression in differentiated cells, with the highest expression observed in cholinergic-like neuronal cells, in which γ-enolase promoted neurite outgrowth and enhanced differentiation. Overexpression of full-length γ-enolase enhanced neurite outgrowth but decreased cytoskeletal marker expression in differentiated cells, whereas it increased cytoskeletal marker expression in non-differentiated cells. Confocal microscopy revealed increased co-localization of γ-enolase and cathepsin X in differentiated cells, particularly in cholinergic-like neuronal cells. Furthermore, inhibition of cathepsin X increased active γ-enolase expression and enhanced neuronal differentiation. These findings suggest that γ-enolase plays an important role in neuronal differentiation, particularly in cholinergic-like cells, with its regulation by cathepsin X contributing to the differentiation process.

## Materials and Methods

### Reagents

Retinoic acid (RA; R2625, Sigma-Aldrich, St. Louis, MO, USA) was prepared as a 10 mM stock solution in dimethyl sulfoxide (DMSO; Sigma-Aldrich). Phorbol 12-myristate 13-acetate (PMA; P1585), N6,2′-O-dibutyryladenosine 3′,5′-cyclic monophosphate sodium salt (dbcAMP; D0260), and brain-derived neurotrophic factor human (BDNF; B3795) were from Sigma-Aldrich. The γ-Eno peptide, corresponding to the last 30 amino acids of γ-enolase (AKYNQLMRIEEELGDEARFAGHNFRNPSVL), was obtained from GenScript (Piscataway, NJ, USA) and prepared as a 0.712 mM stock solution in Dulbecco’s phosphate-buffered saline (DPBS; D8537, Sigma-Aldrich). The irreversible inhibitor of cathepsin X, AMS36, was synthesized as reported by Pišlar et al. (Pišlar et al., 2014) and prepared as a 10 mM stock solution in DMSO.

### Cell Cultures

The human neuroblastoma SH-SY5Y and murine neuroblastoma Neuro-2a cell lines were obtained from the American Type Culture Collection (CRL-2266 and CCL-131, respectively; Manassas, VA, USA). Cells were cultured in Dulbecco’s modified Eagle’s medium (DMEM)/F12 (D8437, Sigma-Aldrich) supplemented with 10% (v/v) fetal bovine serum (FBS; Thermo Fisher Scientific, Waltham, MA, USA) and 1% penicillin-streptomycin (P4333, Sigma-Aldrich). The human neuroblastoma LA-N-2 cell line was obtained from Leibniz Institute DSMZ-German Collection of Microorganisms and Cell Cultures GmbH (ACC 671; Braunschweig, Germany). Cells were cultured in DMEM/F12 (D8437, Sigma-Aldrich) supplemented with 20% (v/v) FBS (Thermo Fisher Scientific) and 2% penicillin-streptomycin (P4333, Sigma-Aldrich). Cells were maintained at 37 °C in a humidified atmosphere containing 5% CO_2_ and grown to 80% confluence. Cells were grown in 75 cm^2^ flasks, passaged twice a week when approximately 80% confluent, and used only up to passage 30.

### Cell Differentiation and Treatment

To differentiate the SH-SY5Y cell line into dopaminergic-, cholinergic-, and adrenergic-like neuronal cells, we adapted protocols from Kovalevich and Langford (Kovalevich & Langford, 2013), Shipley et al. (Shipley et al., 2016), Medeiros et al. (Medeiros et al., 2019), and Kume et al. (Kume et al., 2008). For differentiation experiments, plates were pre-coated with collagen (20 µg/mL; C3867; Sigma-Aldrich) in Dulbecco’s phosphate-buffered saline (DPBS; D8537, Sigma-Aldrich) for 2 h at 37 °C and washed twice with DPBS. SH-SY5Y cells were then seeded, and after 1 day, the cells were exposed to reduced-serum medium (2% FBS and 0.25% penicillin-streptomycin) containing 10 µM RA. Differentiation medium with the same proportion of DMSO was used as a negative control, representing non-differentiated SH-SY5Y cells. After 72 h, SH-SY5Y cell differentiation into the dopaminergic-like neuronal subtype was induced with reduced-serum medium (2% FBS and 0.25% penicillin-streptomycin) containing a combination of 10 µM RA and 80 nM PMA.

Cell differentiation into the cholinergic-like neuronal subtype was induced with reduced-serum medium (2% FBS and 0.25% penicillin-streptomycin) containing a combination of 10 µM RA and 50 ng/mL BDNF for an additional 72 h. To obtain the adrenergic-like neuronal subtype, the day after seeding, SH-SY5Y cells were exposed to reduced-serum medium containing 0.5 mM dbcAMP for 72 h. All control cells were treated with an equivalent volume of the solvent used for RA (DMSO) or dbcAMP (dH_2_O) and were characterized as non-differentiated SH-SY5Y cells.

To differentiate Neuro-2a cells into a dopaminergic-like neuronal subtype, we adapted the protocol from Tremblay et al. (Tremblay et al., 2010). Cells were seeded onto non-coated plates and the next day exposed to reduced-serum medium (2% FBS and 0.25% penicillin-streptomycin) containing 0.5 mM dbcAMP for 72 h. All control cells were treated with an equivalent volume of the solvent used for dbcAMP (dH_2_O) and were characterized as non-differentiated Neuro-2a cells.

To differentiate LA-N-2 cells into a cholinergic-like neuronal subtype, we adapted the protocol from Singh et al. (Singh et al., 1990). Cells were seeded onto non-coated plates and the next day exposed to reduced-serum medium (4% FBS and 0.5% penicillin-streptomycin) containing 10 µM RA for 72 h. All control cells were treated with an equivalent volume of the solvent used for RA (DMSO) and were characterized as non-differentiated LA-N-2 cells.

For γ-Eno peptide treatment, the peptide was added to the differentiation medium at a final concentration of 100 nM on days 1 and 4. Additionally, for cathepsin X inhibition, AMS36 at a final concentration of 10 µM was added to differentiation media on days 1 and 4. Non-treated control cells were treated with an equal volume of the solvent used for γ-Eno (DPBS) and AMS36 (DMSO).

### Vector Construction

Human enolase 2 VersaClone cDNA (RDC3186; R&D Systems, Minneapolis, MN, USA) and the pcDNA3-GFP expression vector (13031; Addgene, Watertown, MA, USA) were used. The following primers were used for cloning: 5′-TTGTACAAGTCCATAGAGAAGATCTGG-3′ and 5′-AATCTAGATCACAGCACACTGG-3′ for enolase 2, and 5′-TTGTACAAGTCCATAGAGAAGATCTGG-3′ and 5′-AATCTAGATCAACTGGGATTACG-3′ for enolase 2 without the last two amino acids on its C-terminus (ΔC). PCR products were ligated into the *XbaI* and *BsrGI* sites of pcDNA3-GFP, placing enolase 2 in-frame downstream of GFP and resulting in N-terminal GFP fusions to enolase 2 or enolase 2-ΔC. Prepared vectors were multiplied in *Escherichia coli* TOP10. Enolase 2 and enolase 2-ΔC genes were verified by sequencing.

### Cell Transfection

For the overexpression of γ-enolase, SH-SY5Y cells were seeded in growth medium without antibiotics into 6-well culture plates (1.5 × 10^5^ cells/mL for the 7-day differentiation protocol and 3 × 10^5^ cells/mL for the 4-day differentiation protocol). The next day, lipofection was performed using Lipofectamine 2000 (11668027; Thermo Fisher Scientific) according to the manufacturer’s instructions. Briefly, 2 µg of DNA plasmid in 100 µL of serum-free medium without antibiotics was mixed with an equal amount of medium containing 4 µL of Lipofectamine and incubated for 20 min at room temperature (RT). The DNA-lipid mixture was added to the cells dropwise and incubated at 37 °C and 5% CO_2_ for 5 h. For the 7-day cell differentiation protocol, cells were exposed to another transfection in the same manner on day 4. After transfection, the transfection medium was removed and replaced with differentiation medium as indicated in the *Cell differentiation and treatment* section. The plasmids used for γ-enolase overexpression were constructed using the pcDNA3-GFP vector, which contains a constitutively active cytomegalovirus (CMV) promoter. Consequently, the expression of GFP–γ-enolase fusion proteins is not under the control of differentiation agents such as RA, PMA, dbcAMP, or BDNF, as these treatments do not target any inducible promoter elements within the plasmid. To assess the efficiency of transfection, cells were visualized with fluorescence microscopy and the EVOS Cell Imaging System (Thermo Fisher Scientific) for qualitative assessment of protein expression and cell morphology and viability. Afterwards, cells were harvested for cell pellets, and the efficiency of γ-enolase transfection was evaluated by western blot analysis. Two specific mouse monoclonal antibodies were used: one raised against amino acids 416–433 of γ-enolase, which detects the C-terminal region and therefore recognizes only the full-length variant (1:250; sc-21738, Santa Cruz Biotechnology, Dallas, TX, USA), and another raised against amino acids 271–285, which detects both the full-length and C-terminally truncated (ΔC) variants, representing total γ-enolase expression (1:350; sc-21737, Santa Cruz Biotechnology).

For γ-enolase silencing, Neuro-2a and LA-N-2 cells were seeded in growth medium without antibiotics into 12-well culture plates (1 × 10^5^ cells/mL). For mouse γ-enolase, Silencer^®^ siRNA (s65514; Thermo Fisher Scientific) targeting the sequence 5′-GGACUUUGUCCGGAACUAUtt-3′ (sense) and 5′-AUAGUUCCGGACAAAGUCCtg-3′ (antisense) was used. For human γ-enolase, Stealth RNAi™ siRNA specific for ENO2 (HSS176535; Thermo Fisher Scientific) was applied, targeting the sequence 5′-CGACUAGGUGCAGAGGUCUACCAUA-3’. Cells were transfected with control siRNA (sc-37007; Santa Cruz Biotechnology) as control to siRNA of mRNA target. Cells were transiently transfected with Stealth RNAi/γ-Eno using Lipofectamine 2000 (Thermo Fisher Scientific), according to the manufacturer’s protocol. Briefly, Lipofectamine 2000 was gently mixed before use, diluted in serum-free medium without antibiotics, and left for 5 min at RT. The diluted Lipofectamine 2000 was then combined with the diluted siRNA oligomer in serum-free medium without antibiotics, gently mixed, and left for a further 20 min at RT. Next, 200 µL of the transfection complex was added to the cells to achieve a final siRNA concentration of 20 nM in the well and incubated at 37 °C in a humidified atmosphere of 5% CO_2_ for 5 h. The transfection medium was removed and replaced with differentiation medium as indicated in the *Cell differentiation and treatment* section. To assess the efficiency of transfection, cells were harvested for cell pellets, and the efficiency of γ-enolase transfection was evaluated by western blot analysis.

### Evaluation of Cell Extensions

SH-SY5Y, Neuro-2a, and LA-N-2 cells were seeded in growth medium into 6-well culture plates in duplicate. The next day, cells were treated with differentiation medium as described above. Cell extensions were evaluated by morphological examination and captured using the EVOS Cell Imaging System (Thermo Fisher Scientific). ImageJ software was used to determine the number and length of cell extensions (in pixels) that were longer than the corresponding cell diameter. Approximately 100 cells per condition were measured in each experiment. The data are expressed relative to the control, i.e., non-differentiated cells.

### Cell Proliferation Assay

SH-SY5Y, Neuro-2a, and LA-N-2 cells were seeded in growth media in 6-well culture plates in duplicate. The next day, cells were stained with CellTrace carboxyfluorescein succinimidyl ester (CFSE) reagent (1 µM; Thermo Fisher Scientific) according to the manufacturer’s protocol. Subsequently, the cells were differentiated as described above. Then, cells were collected, and their mean fluorescence intensities were measured in the BL-1 channel using a flow cytometer (Attune NxT, Thermo Fisher Scientific). The obtained data were analyzed in FlowJo software (Tree Star Inc., Ashland, OR, USA), and mean CFSE fluorescence intensities were normalized to the control cells. Three independent experiments with two replicates per differentiation condition were performed.

### Cell Lysate Preparation

After 4 days of differentiation for Neuro-2a, LA-N-2, and dbcAMP-treated SH-SY5Y cells, or after 7 days of differentiation for SH-SY5Y cells treated with RA combined with PMA or BDNF, the cells were harvested. Cell lysis was completed with either of two lysis buffers: for assessing protein expression by western blotting and enzyme-linked immunosorbent assay (ELISA) (50 mM HEPES, pH 6.5, 150 mM NaCl, 1 mM EDTA, 0.25% Triton X-100) or for quantifying cathepsin X activity (50 mM Na-acetate, pH 5.5, 1 mM EDTA, 100 mM NaCl, 0.25% Triton X-100). After lysis, whole-cell lysates were incubated for 30 min on ice, preserved at − 80 °C, subjected to freeze-thaw cycles, sonicated, and centrifuged at 15,000 × g for 15 min at 4 °C. Protein concentrations in the obtained lysates were quantified using the DC Protein Assay (Bio-Rad, Hercules, CA, USA) and bovine serum albumin (BSA; Sigma-Aldrich) as a protein standard.

### Western Blotting

Equal protein concentrations from whole-cell lysates (1 µg/µL) were denatured by the addition of SDS-PAGE buffer, heated (100 °C) for 5 min, and resolved by SDS-PAGE on 12% Tris-glycine gels. The proteins were then transferred to polyvinylidene difluoride membranes using iBlot (Thermo Fisher Scientific). Membranes were blocked in 5% (w/v) non-fat dried milk powder in Tris-buffered saline with Tween 20 (TBST; 20 mM Tris/HCl, pH 7.4, 137 mM NaCl, and 0.1% Tween 20) at RT for 1 h. Subsequently, the membranes were incubated overnight at 4 °C with primary antibodies in TBST containing 3% (w/v) BSA. The following primary antibodies and dilutions were used: mouse anti-tyrosine hydroxylase (1:250; sc-25269), mouse anti-acetylcholinesterase (1:500; sc-373901), mouse anti-α-enolase (1:500; sc-100812), mouse anti-γ-enolase raised against amino acids 416–433 of γ-enolase, determining the active form of γ-enolase (1:250; sc-21738, Santa Cruz Biotechnology), mouse anti-γ-enolase raised against amino acids 271–285 of γ-enolase, determining the total form of γ-enolase (1:350; sc-21737;Santa Cruz Biotechnology), rabbit anti-α−2 adrenergic receptors (ADRA2A) (1:300; 14266-1-AP), rabbit anti-glyceraldehyde-3-phosphate dehydrogenase (GAPDH; 1:10000; 10494-1-AP; both from Proteintech, Rosemont, IL, USA), mouse anti-β-tubulin (1:1000; T4026; Sigma-Aldrich), and goat anti-cathepsin X (1:500; AF934; R&D Systems, Minneapolis, MN, USA). The membrane for goat anti-cathepsin X antibody incubation was blocked with 1.5% (w/v) non-fat dried milk powder in TBST and diluted in TBST containing 1.5% non-fat dried milk powder and 1% (w/v) BSA. After washing, the following secondary horseradish-peroxidase-conjugated antibodies in TBST containing 5% (w/v) non-fat dried milk powder were added for 1 h: anti-mouse (1:5000; 111-035-068), anti-rabbit (1:5000; 111-035-045; both from Jackson ImmunoResearch, West Grove, PA, USA), and anti-goat (1:2000; sc-2354; Santa Cruz Biotechnology) antibodies. After washing, protein bands were visualized with enhanced chemiluminescence detection kits (Thermo Fisher Scientific) and recorded with a G: Box imager (Syngene, UK). When necessary, membranes were stripped with stripping buffer (62.5 mM Tris/HCl, pH 5.7, 100 mM 2-mercaptoethanol, and 2% SDS) for 1 h at 65 °C. Band intensities were quantified using Gene Tools software (Sygene). To determine the protein levels, values were expressed as a ratio to GAPDH and were normalized to the control sample.

### ELISA

The protein levels of α-enolase and γ-enolase were detected with the ENO1 SimpleStep ELISA^®^ (ab181417; Abcam) and NSE SimpleStep ELISA^®^ (ab217778; Abcam) kits, respectively. Following the manufacturer’s instructions, standards and equal protein amounts of samples (50 µg) were added to the wells, followed by the antibody mix. After 1 h incubation, the wells were washed, and TMB Development Solution (Merck Millipore, MA, USA) was added. After 10 min, the reaction was stopped by adding Stop Solution. Protein amounts were determined by measuring the absorbance at 450 nm using a Tecan Safire spectrophotometer (Tecan Safire^2^), and protein levels were expressed relative to the control.

Cathepsin X protein levels were determined by ELISA. Microtiter plates were coated with equal aliquots of goat anti-cathepsin X antibody (1:250, AF934; R&D Systems) in 0.01 M carbonate/bicarbonate buffer, pH 9.6, and incubated at 4 °C overnight. After incubation with blocking buffer (2% BSA in PBS, pH 7.4) for 1 h at RT, samples containing equal protein amounts (25 µg) were added. Following a 2-h incubation at 37 °C, the wells were washed and filled with mouse anti-cathepsin X monoclonal antibody conjugated with horseradish peroxidase (1:3000; 3B10; produced in-house) in a blocking buffer. After an additional 1.5 h incubation at 37 °C, 100 µL of 3,3′,5,5′-tetramethylbenzidine substrate (Sigma-Aldrich) was added to each well. The reaction was stopped after 15 min by adding 50 µL of 2 M H_2_SO_4_ to each well. Protein levels were determined by measuring the absorbance at 450 nm using a Tecan Safire spectrophotometer (Tecan Safire^2^, Switzerland), and protein levels were expressed relative to the control.

### Cathepsin X Activity Assay

Cell lysates were diluted to a protein concentration of 0.125 mg/mL in 50 mM acetate buffer, pH 5.5, with 5 mM dithiothreitol (43819; Sigma-Aldrich) and 1.5 mM EDTA (03690; Sigma-Aldrich) followed by incubation at 37 °C for 10 min. In duplicate, 95 µL of lysate was transferred into the wells of a black microtiter plate (Nunclon Delta Surface; Thermo Fisher Scientific) containing 5 µL of the previously added fluorogenic substrate Abz-FEK(Dnp)-OH (Puzer et al., 2005) at a final concentration of 5.9 µM. The subsequent degradation was monitored continuously at 320 ± 5 nm excitation and 420 ± 5 nm emission using a Tecan Safire spectrophotometer (Tecan Safire^2^, Switzerland). Kinetic measurements were analyzed with Magellan™ software (Magellan™, 2011), and the resulting data, expressed in relative fluorescence units, were normalized to the control sample.

### Enolase Glycolytic Activity Assay

The glycolytic activity of enolase, i.e., the conversion of 2-phosphoglycerate to phosphoenolpyruvate, was assessed with the Enolase Assay Kit (ab241024; Abcam), according to the manufacturer’s instructions. Briefly, cell lysates were prepared using the Enolase buffer supplied in the Enolase Assay Kit, and protein concentrations were determined as described in the previous paragraph. Initially, standards and samples were prepared (1 µg/mL) and loaded in duplicate onto the plate, forming two separate sets of samples. Additionally, 100-fold and 1000-fold dilutions of the positive control were prepared and loaded onto the plate in duplicate. The reaction and a corresponding background control mixture (substituting substrate with buffer) were formulated and applied to the plate. Absorbance was measured at 570 nm using a Tecan Safire spectrophotometer in kinetic mode for 60 min at RT. Sample absorbances were corrected using their respective background controls. Results were expressed as the slope of the linear portion of the reaction curve over time, with data normalized to control cells.

### Flow Cytometry Analysis of Protein Expression

Flow cytometry was used to analyze the protein expression of tyrosine hydroxylase (TH), choline acetyltransferase (ChAT), and ADRA2A in treated cells. For all experiments, cells were washed with PBS (pH 7.4), fixed with 4% paraformaldehyde (1510; Electron Microscopy Science, Hatfield, PA, USA) for 10 min at RT, and permeabilized with specific reagents. For TH detection, cells were permeabilized with 90% methanol at − 20 °C for 30 min, followed by incubation with anti-TH antibody (1:2500; ab209921, Abcam) at RT for 30 min. A rabbit monoclonal IgG phycoerythrin-conjugated isotype control (1:2500; ab209478, Abcam) was used under the same conditions. ChAT expression was assessed after permeabilization with 0.05% Triton X-100 in PBS for 15 min at RT, followed by incubation with anti-ChAT antibody (1:5000; ab224001, Abcam) for 30 min at RT. A rabbit monoclonal IgG allophycocyanin-conjugated isotype control (Abcam, ab232814) was included as a control. For ADRA2A detection, cells were permeabilized with 0.5% Tween 20 in PBS for 10 min at RT and incubated with anti-ADRA2A antibody (1:50; PA5-18475, Thermo Fisher Scientific) at 4 °C for 60 min, followed by Alexa Fluor 488-labeled secondary antibody (1:500; Thermo Fisher Scientific) for 30 min at RT. After antibody incubations, cells were washed and collected. Mean fluorescence intensities were measured using an Attune NxT flow cytometer (Thermo Fisher Scientific) in the BL-1 channel for TH and ADRA2A and the BL-2 channel for ChAT.

The relative TH, ChAT, and ADRA2A contents of the cells were evaluated using FlowJo software and were determined as the mean fluorescence of each sample relative to the fluorescence intensity of control cells.

### Double Immunofluorescence Staining

Cells were seeded onto glass coverslips in growth medium in duplicate and subjected to the differentiation protocol. Afterwards, cells were fixed with 4% paraformaldehyde (1510; Electron Microscopy Science) in DPBS for 30 min at RT and then permeabilized with 0.5% Tween 20 (P9416; Sigma-Aldrich) in PBS for 10 min. Non-specific staining was blocked with 10% donkey serum (S-30; Sigma-Aldrich) in PBS with 0.05% Triton X-100 (T8787; Sigma-Aldrich) for 30 min at RT. Cells were then incubated with rabbit phycoerythrin (PE)-conjugated anti-TH (1:200; ab209921; Abcam), rabbit allophycocyanin (APC)-conjugated anti-ChAT (1:200; ab224001; Abcam), rabbit Alexa Fluor 555 anti-β-tubulin (1:200; ab202519; Abcam), goat anti-ADRA2A (1:50; PA5-18475; Thermo Fisher Scientific), rabbit anti-α-enolase (1:200; 11204-1-AP; Proteintech, Rosemont, IL, USA), mouse anti-γ-enolase (1:50; 66150-1-Ig; Proteintech), and goat anti-cathepsin X (1:75; AF934; R&D System) antibodies in blocking solution for 2 h at RT. Afterwards, the cells were washed with PBS, and those incubated with non-conjugated primary antibodies were further exposed to Alexa Fluor 488-, Alexa Fluor 555-, and Alexa Fluor 647-labeled secondary antibodies (1:1000; Thermo Fisher Scientific) for an additional 1.5 h. After washing with PBS, a ProLong Gold antifade reagent with DAPI (P36935; Thermo Fisher Scientific) was used for mounting the coverslips onto glass slides. Fluorescence microscopy was performed using a confocal microscope (LSM 710; Carl Zeiss, Oberkochen, Germany and AX; Nikon, Tokyo, Japan) with the ZEN 3.4 image software or NIS-Elements image software. Co-localization analysis was performed on immunofluorescence images using Carl Zeiss ZEN software. The degree of co-localization was quantified using the Pearson’s correlation coefficient (referred to as Correlation R). Relative co-localization areas were analyzed in at least 10 cells per condition.

### Statistical Analysis

All statistical analyses were performed using parametric tests appropriate to the experimental design. Data are expressed as means with 95% confidence intervals (CI) from N independent biological replicates, with N specified in the corresponding figure legends. Differences between groups were assessed by one-way analysis of variance (ANOVA) followed, where appropriate, by Tukey’s post hoc multiple comparisons test. Analyses were conducted using GraphPad Prism version 10 (GraphPad Software, San Diego, CA, USA). Exact p-values are reported in the figure legends; for graphical representation, statistical significance is indicated as *p* < 0.05 (*), and *p* < 0.01 (**). Sample-size justification for key experiments was based on power analyses performed with G*Power 3.1, using effect sizes estimated from the data, an α-level of 0.05, and a target power of 0.80 (Supplementary Table S1).

## Results

### Differentiated Neuronal-like Cells Exhibit Subtype-specific Markers and Distinct γ-Enolase Expression Patterns

After successful differentiation of neuronal cell lines, cells should exhibit the morphological, biochemical, and functional characteristics of specific neuronal subtypes (Agholme et al., 2010). In the current study, differentiation protocols were adapted to induce specific neuronal subtypes in SH-SY5Y, Neuro-2a and LA-N-2 cells. In SH-SY5Y cells, dopaminergic differentiation was induced with RA and PMA in reduced-serum media to generate dopaminergic-like neuronal cells, cholinergic differentiation was induced with RA and BDNF in reduced-serum media to generate cholinergic-like neuronal cells, and adrenergic differentiation was induced with dbcAMP in reduced-serum media to generate adrenergic-like neuronal cells. Cells exhibited enhanced neurite outgrowth (Suppl. Fig. S1A) and decreased cell proliferation (Suppl. Fig. S1B) with differentiation into dopaminergic- and cholinergic-like neuronal cells, specifically at days 7 and 10. Day 7 was chosen as the optimal timepoint, as cells displayed well-defined neuronal features, including extensive neurite outgrowth, while maintaining experimental consistency.After differentiation under the optimized protocol, we analyzed the morphological characteristics, proliferation rates, and expression of specific markers to validate the protocols for differentiation into each neuronal subtype. Neuron-like morphological changes were observed after differentiation into dopaminergic- (Suppl. Fig. S2A), cholinergic- (Suppl. Fig. S2B), and adrenergic-like (Suppl. Fig. S2C) neuronal cells. In dopaminergic-like (Suppl. Fig. S1D) and cholinergic-like (Suppl. Fig. S2F) neuronal cells, neurite lengths were up to three times greater than cell diameters, whereas adrenergic differentiation resulted in a smaller increase in neurite length (Suppl. Fig. S2H). Control SH-SY5Y cells cultured in reduced-serum media exhibited polygonal cell morphology with few short extensions, whereas differentiated cells exhibited several neuritic projections that formed connections with surrounding cells. Dopaminergic-like and adrenergic-like neuronal cells showed a branched morphology, characterized by new bud formation and additional growth centers. Conversely, cholinergic-like neuronal cells developed the longest neurites, as revealed by semi-quantitative analysis of cell extensions. Differentiation was further confirmed by proliferation analysis, which revealed significantly decreased cell proliferation, as indicated by elevated CFSE levels, in dopaminergic- (Suppl. Fig. S2E), cholinergic- (Suppl. Fig. S2G), and adrenergic-like neuronal cells (Suppl. Fig. S2I).To further validate differentiation, subtype-specific neuronal markers were used. Immunofluorescence and western blot analyses of dopaminergic-like neuronal cells showed distinct increased TH expression, indicating differentiation into a dopaminergic-like subtype (Suppl. Fig. S2J, S2M). Conversely, cholinergic-like neuronal cells exhibited increased fluorescence intensity of ChAT (Suppl. Fig. S2K, immunofluorescent analysis) and increased acetylcholinesterase expression (Suppl. Fig. S2N, western blot), suggesting cholinergic neuron-like differentiation. Adrenergic-like neuronal cells exhibited increased ADRA2A expression compared to controls, as confirmed by confocal microscopy and western blot analysis (Suppl. Fig. S2L, S2O), indicating adrenergic neuron-like differentiation. Additionally, flow cytometry analysis revealed similarly increased expression patterns for TH, ChAT, and ADRA2A in the corresponding neuronal subtypes (Suppl. Fig. S3). These findings confirm the suitability of our differentiation protocols for obtaining dopaminergic-, cholinergic-, and adrenergic-like neuronal cells from SH-SY5Y cells.

**Fig. 1:**
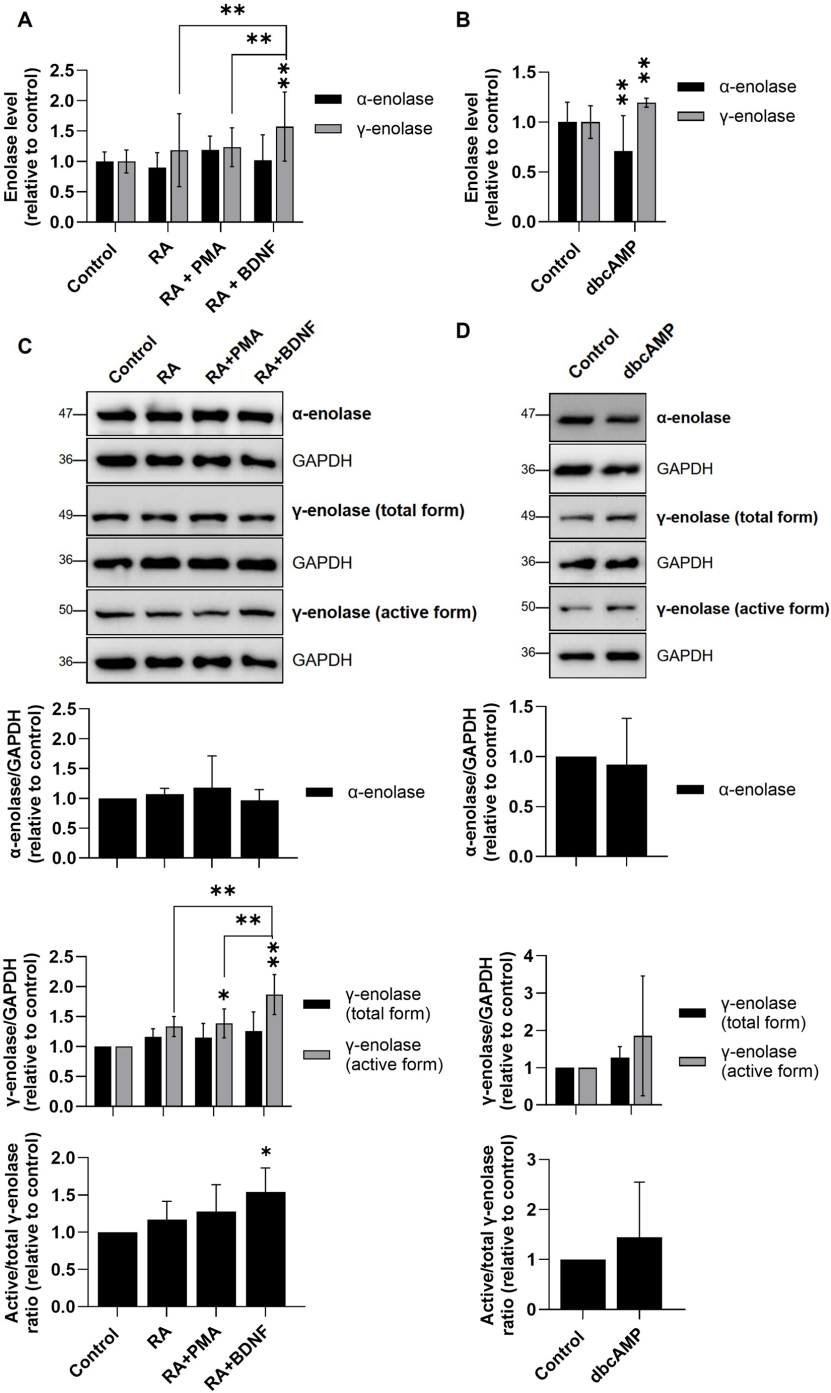
Expression patterns of α-enolase and γ-enolase in SH-SY5Y cells differentiated into specific neuronal subtypes. (**A**,** B**) ELISA results of α-enolase and γ-enolase expression in (**A**) dopaminergic-like neuronal cells differentiated with retinoic acid (RA) and phorbol 12-myristate 13-acetate (PMA) and cholinergic-like neuronal cells differentiated with RA and brain-derived neurotrophic factor (BDNF) and (**B**) adrenergic-like cells differentiated with dibutyryl cyclic AMP (dbcAMP). Data are shown as mean ± 95% CI from *N* = 2 independent experiments, each performed in duplicate. Statistical significance was assessed by one-way ANOVA followed by Tukey’s post hoc test. Exact p-values: (**A**) α-enolase, *p* = 0.108; γ-enolase - Control vs. RA, *p* = 0.582, Control vs. RA + PMA, *p* = 0.061; Control vs. RA + BDNF, *p* = 0.001; RA vs. RA + PMA, *p* = 0.205; RA vs. RA + BDNF, *p* = 0.001; RA + PMA vs. RA + BDNF, *p* = 0.001; (**B**) α-enolase - Control vs. dbcAMP, *p* = 0.005; γ-enolase – Control vs. dbcAMP, *p* = 0.005. (**C**,** D**) Representative western blots (top) and quantification (bottom) of the expression of α-enolase and γ-enolase and the active-to-total γ-enolase ratio in (**C**) dopaminergic-, cholinergic-, and (**D**) adrenergic-like neuronal cells. Protein levels are normalized to GAPDH. Data are shown as mean ± 95% CI from *N* = 2–7 independent experiments. Statistical significance was assessed by one-way ANOVA followed by Tukey’s post hoc test. Exact p-values: (**C**) α-enolase, *p* = 0.660; γ-enolase (total form), *p* = 0.224; γ-enolase (active form) - Control vs. RA, *p* = 0.070, Control vs. RA + PMA, *p* = 0.029; Control vs. RA + BDNF, *p* = 0.001; RA vs. RA + PMA, *p* = 0.900; RA vs. RA + BDNF, *p* = 0.002; RA + PMA vs. RA + BDNF, *p* = 0.005; γ-enolase (active/total form) - Control vs. RA, *p* = 0.726, Control vs. RA + PMA, *p* = 0.381; Control vs. RA + BDNF, *p* = 0.019; RA vs. RA + PMA, *p* = 0.900; RA vs. RA + BDNF, *p* = 0.158; RA + PMA vs. RA + BDNF, *p* = 0.425; (**D**) α-enolase - Control vs. dbcAMP, *p* = 0.378; γ-enolase (total from) - Control vs. dbcAMP, *p* = 0.130; γ-enolase (active from) - Control vs. dbcAMP, *p* = 0.150; γ-enolase (active/total from) - Control vs. dbcAMP, *p* = 0.123. Data were obtained after 7 days of differentiation into dopaminergic- and cholinergic-like neuronal cells and after 4 days of differentiation into adrenergic-like cells, and are expressed relative to the control

The glycolytic enzyme γ-enolase is well established as a general marker for mature neurons in the central nervous system (Isgrò et al., 2015). Nevertheless, we investigated whether γ-enolase expression and activity differ among distinct neuronal subtypes, which would suggest a potential subtype-specific role. Glycolytic enolase activity was unchanged in the dopaminergic- and cholinergic-like neuronal subtypes, but increased in the adrenergic-like neuronal subtype (Suppl. Fig. S4). ELISA assays revealed that α-enolase expression remained similar between control and differentiated SH-SY5Y cells, whereas γ-enolase expression was increased in dopaminergic-, cholinergic- (Fig. 1A), and adrenergic-like neuronal cells (Fig. 1B). The largest increase was observed in cholinergic-like neuronal cells, highlighting particularly an association between γ-enolase expression and cholinergic differentiation. Furthermore, western blot analyses showed similar expression of α-enolase in differentiated SH-SY5Y cells and increased expression of γ-enolase in its total form in differentiated SH-SY5Y cells compared to control cells (Fig. 1C and D). Nevertheless, the levels of active γ-enolase followed a similar trend as those of the total form of γ-enolase, with the most pronounced expression observed in cholinergic-like neuronal cells, further emphasizing the prominent role of γ-enolase in this neuronal subtype. Importantly, the active-to-total γ-enolase ratio was most prominently increased in cholinergic-like neuronal cells, suggesting a more prominent functional role of γ-enolase in cholinergic-like neuronal cells.

**Fig. 2:**
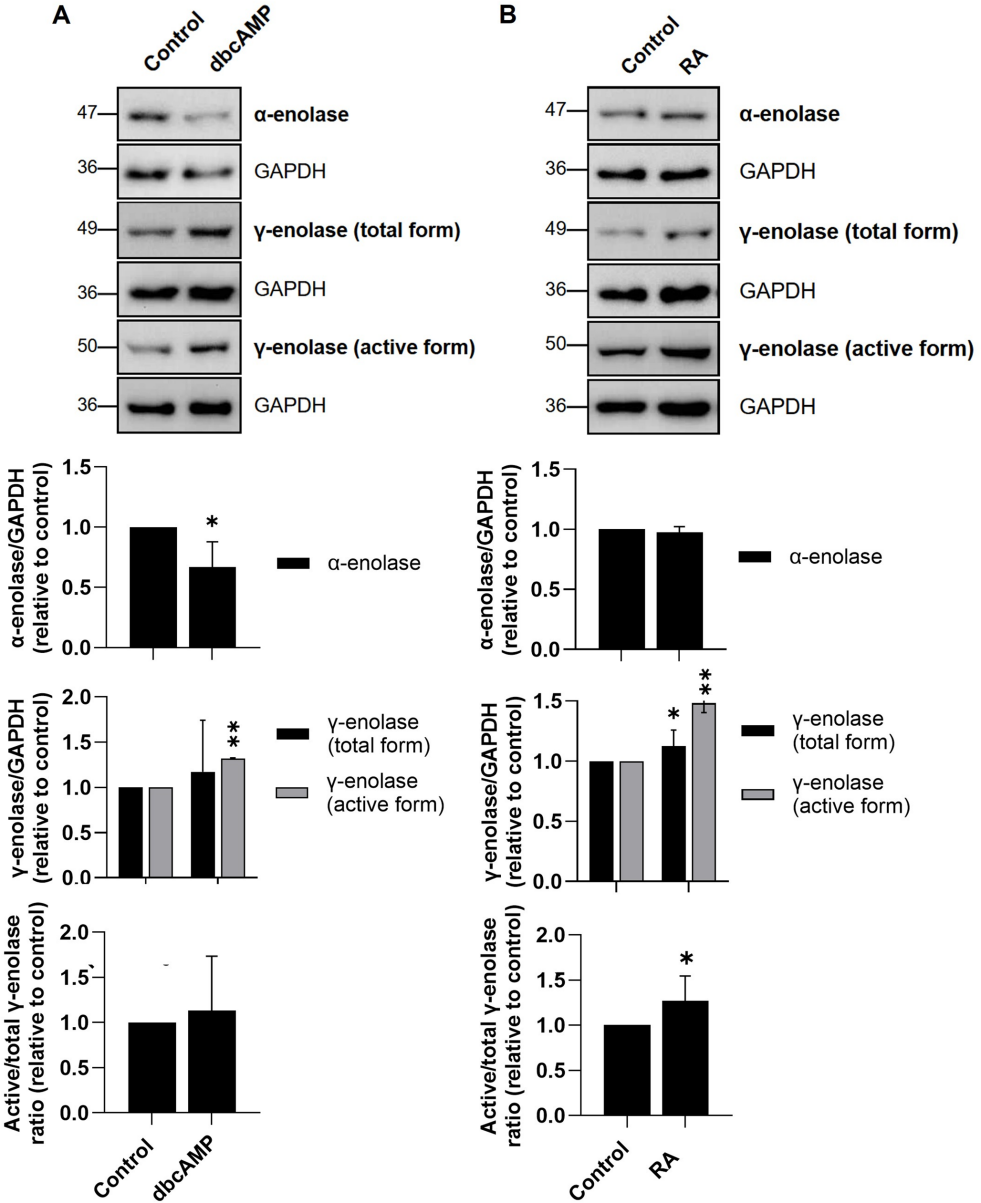
Expression patterns of α-enolase and γ-enolase in Neuro-2a and LA-N-2 cells differentiated into specific neuronal subtypes. **(A**,** B**) Representative western blots (top) and quantification (bottom) of the expression of α-enolase and γ-enolase in (**A**) Neuro-2a and (**B**) LA-N-2 cells after differentiation. Protein levels are normalized to GAPDH. Data are shown as mean ± 95% CI from *N* = 2–4 independent experiments. Statistical significance was assessed by one-way ANOVA followed by Tukey’s post hoc test. Exact p-values: (**A**) α-enolase - Control vs. dbcAMP, *p* = 0.035; γ-enolase (total form) - Control vs. dbcAMP, *p* = 0.187; γ-enolase (active form) - Control vs. dbcAMP, *p* = 0.001; γ-enolase (active/total form) - Control vs. dbcAMP, *p* = 0.252; (**B**) α-enolase - Control vs. RA, *p* = 0.073; γ-enolase (total form) - Control vs. RA, *p* = 0.024; γ-enolase (active form) - Control vs. RA, *p* = 0.005; γ-enolase (active/total from) - Control vs. RA, *p* = 0.033. Data were obtained after 4 days of differentiation into dopaminergic-like neuronal cells (Neuro-2a), and cholinergic-like neuronal cells (LA-N-2), and are expressed relative to the control

Following the evaluation of α-enolase and γ-enolase expression patterns in the specific neuronal subtypes differentiated from SH-SY5Y cells, we extended our analysis to other neuroblastoma cell lines to confirm our findings. We used the mouse Neuro-2a and human LA-N-2 cell lines, known to differentiate into the dopaminergic- and cholinergic-like neuronal subtypes, respectively (Tremblay et al., 2010; Singh et al., 1990). The subtypes were confirmed through analyses of cell morphology, proliferation rates, and the expression of specific markers for dopaminergic- and cholinergic-like neuronal cells (Suppl. Fig. S5). Our results revealed distinct enolase expression patterns across neuronal subtypes. Western blot analysis revealed decreased α-enolase expression in differentiated Neuro-2a cells expressing the dopaminergic-like subtype (Fig. 2A) and unchanged α-enolase expression in differentiated LA-N-2 cells expressing the cholinergic-like subtype (Fig. 2B), compared to non-differentiated cells. Of note, both total and active γ-enolase levels were increased in differentiated Neuro-2a and LA-N-2 cells, with the highest levels observed in cholinergic-like LA-N-2 cells. Moreover, the active-to-total γ-enolase ratio was increased in both differentiated cell lines, with the most pronounced difference observed in cholinergic-like LA-N-2 cells (Fig. 2), which correlates well with the results obtained from differentiated SH-SY5Y cells (Fig. 1). Collectively, our findings show distinct enolase expression patterns in differentiated neuronal subtypes. The prominence of γ-enolase, particularly its active form, in cholinergic-like neuronal cells suggests a specialized functional role in this subtype.

### Overexpression of γ-Enolase Affects SH-SY5Y Cell Differentiation

To investigate whether γ-enolase plays a role in neuronal differentiation into specific neuronal subtypes, we transfected SH-SY5Y cells with either a plasmid encoding full-length γ-enolase (total γ-enolase pcDNA3-GFP), a plasmid encoding truncated γ-enolase lacking the C-terminus (ΔC γ-enolase pcDNA3-GFP), or an empty control plasmid (pcDNA3-GFP). The specificity of γ-enolase antibodies in detecting either the total or active parts of the protein was confirmed by western blot analysis (Suppl. Fig. S6). In cells transfected with the full-length γ-enolase plasmid, bands were observed at 50 kDa (corresponding to the active form of γ-enolase) and 77 kDa (corresponding to the active form – GFP fusion protein). In cells transfected with either the full-length or truncated γ-enolase plasmid, bands were observed at 48 kDa (corresponding to the total form of γ-enolase) and 77 kDa (corresponding to the total form – GFP fusion protein). Therefore, western blot analysis using the C-terminal-specific antibody confirmed that the active form of γ-enolase was present exclusively in cells expressing the full-length variant. In contrast, the ΔC variant, which is truncated at the C-terminus and lacks the final two amino acids, was not recognized by this antibody, indicating C-terminal truncation and absence of the active form.

**Fig. 3:**
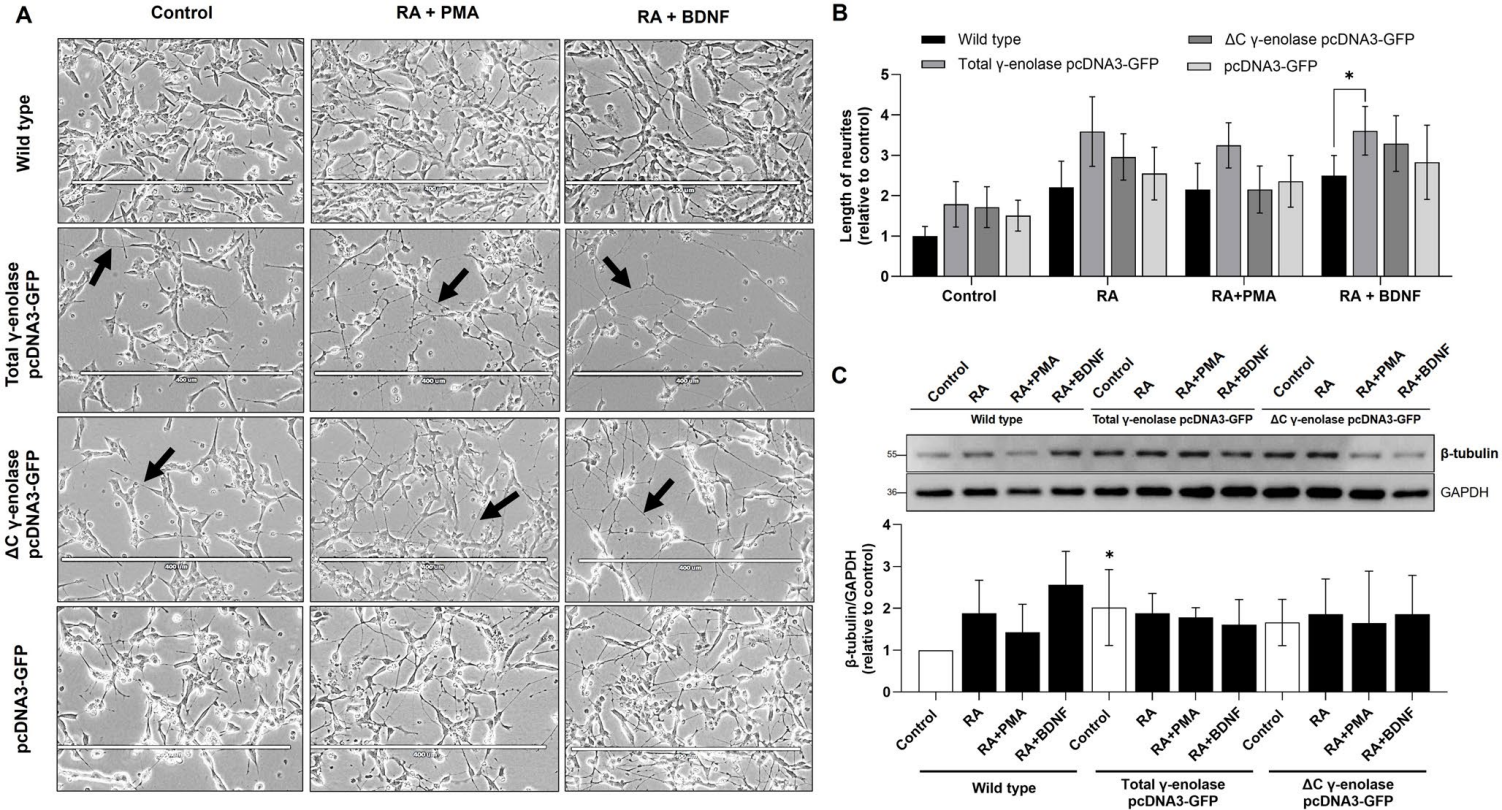
Morphological changes and expression of β-tubulin after γ-enolase upregulation in differentiated SH-SY5Y neuronal subtypes. (**A**) Representative phase-contrast images of SH-SY5Y cells transfected with the ΔC γ-enolase pcDNA3-GFP and total γ-enolase pcDNA3-GFP plasmids. Black arrows indicate cell extensions. Scale bars: 400 μm. (**B**) Neurite lengths were measured in pixels using ImageJ when the extensions were longer than the cell diameter. Data are shown as mean ± 95% CI from *N* = 2 independent experiments. Statistical significance was assessed by one-way ANOVA followed by Tukey’s post hoc test. Exact p-values: Control - Wild type vs. Total γ-enolase pcDNA3-GFP, *p* = 0.051; Wild type vs. ΔC γ-enolase pcDNA3-GFP, *p* = 0.069; Wild type vs. pcDNA3-GFP, *p* = 0.181; Total γ-enolase pcDNA3-GFP vs. ΔC γ-enolase pcDNA3-GFP, *p* = 0.900; Total γ-enolase pcDNA3-GFP vs. pcDNA3-GFP, *p* = 0.541; ΔC γ-enolase pcDNA3-GFP, vs. pcDNA3-GFP, *p* = 0.713; RA – Wild type vs. Total γ-enolase pcDNA3-GFP, *p* = 0.098; Wild type vs. ΔC γ-enolase pcDNA3-GFP, *p* = 0.390; Wild type vs. pcDNA3-GFP, *p* = 0.844; Total γ-enolase pcDNA3-GFP vs. ΔC γ-enolase pcDNA3-GFP, *p* = 0.529; Total γ-enolase pcDNA3-GFP vs. pcDNA3-GFP, *p* = 0.203; ΔC γ-enolase pcDNA3-GFP, vs. pcDNA3-GFP, *p* = 0.744; RA + PMA – Wild type vs. Total γ-enolase pcDNA3-GFP, *p* = 0.064; Wild type vs. ΔC γ-enolase pcDNA3-GFP, *p* = 0.900; Wild type vs. pcDNA3-GFP, *p* = 0.900; Total γ-enolase pcDNA3-GFP vs. ΔC γ-enolase pcDNA3-GFP, *p* = 0.064; Total γ-enolase pcDNA3-GFP vs. pcDNA3-GFP, *p* = 0.116; ΔC γ-enolase pcDNA3-GFP, vs. pcDNA3-GFP, *p* = 0.900; RA + BDNF – Wild type vs. Total γ-enolase pcDNA3-GFP, *p* = 0.042; Wild type vs. ΔC γ-enolase pcDNA3-GFP, *p* = 0.120; Wild type vs. pcDNA3-GFP, *p* = 0.623; Total γ-enolase pcDNA3-GFP vs. ΔC γ-enolase pcDNA3-GFP, *p* = 0.634; Total γ-enolase pcDNA3-GFP vs. pcDNA3-GFP, *p* = 0.123; ΔC γ-enolase pcDNA3-GFP, vs. pcDNA3-GFP, *p* = 0.400. (**C**) Representative western blots (top) and quantification (bottom) of the expression of β-tubulin for dopaminergic-like and cholinergic-like neuronal cells. Protein levels are normalized to GAPDH and expressed relative to the control (wild type). Data are shown as mean ± 95% CI from *N* = 3–4 independent experiments. Statistical significance was assessed by one-way ANOVA followed by Tukey’s post hoc test. Exact p-values: Control - Wild type vs. Total γ-enolase pcDNA3-GFP, *p* = 0.020; Wild type vs. ΔC γ-enolase pcDNA3-GFP, *p* = 0.153; Total γ-enolase pcDNA3-GFP vs. ΔC γ-enolase pcDNA3-GFP, *p* = 0.525; RA – not significant, *p* = 0.998; RA + PMA - not significant, *p* = 0.686; RA + BDNF – not significant, *p* = 0.110. Data were obtained after 7 days of differentiation into dopaminergic- and cholinergic-like neuronal cells and are expressed relative to the control

Morphological analysis revealed significant differences in neurite lengths across neuronal subtypes after SH-SY5Y cell transfection and differentiation. Cells transfected with the total γ-enolase plasmid consistently showed increased neurite outgrowth compared to those transfected with the ΔC γ-enolase plasmid or control vector (Fig. 3A). In dopaminergic-like neuronal cells, neurite outgrowth was enhanced in cells overexpressing the total γ-enolase compared to wild-type cells. Likewise, in cholinergic-like neuronal cells, neurite outgrowth was enhanced in cells overexpressing total γ-enolase. Moreover, neurites were longer compared to dopaminergic-like or adrenergic-like neuronal cells (Suppl. Fig. S7A). Of note, the truncated ΔC γ-enolase plasmid did not increase neurite length to the same extent as the total γ-enolase plasmid, highlighting the functional importance of full-length γ-enolase in promoting neurite elongation. In dopaminergic- and adrenergic-like neuronal cells, transfection with the ΔC γ-enolase plasmid resulted in neurite lengths similar to those in wild type controls. In cholinergic-like neuronal cells, transfection with the ΔC γ-enolase plasmid resulted in increased neurite length compared to the wild type control; however, this increase was slightly less pronounced than that observed with the total γ-enolase plasmid (Fig. 3A).

**Fig. 4:**
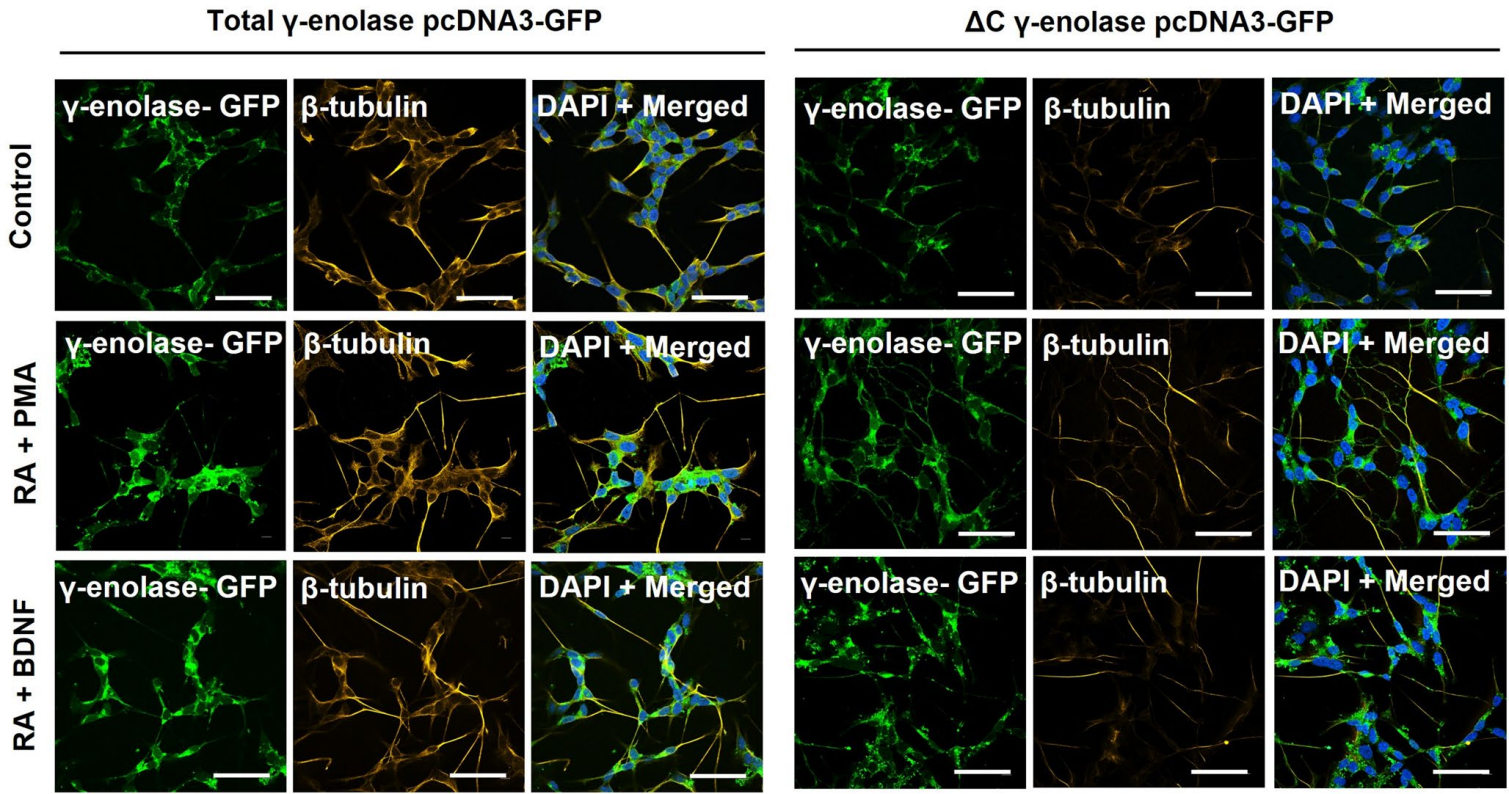
Immunofluorescence images of β-tubulin expression after γ-enolase upregulation in differentiated SH-SY5Y neuronal subtypes. Representative images of immunofluorescence staining for β-tubulin (*yellow*) in SH-SY5Y cells transfected with plasmids (*green*). Nuclei were counterstained with DAPI (*blue*). Scale bars: 50 μm. Two independent experiments (*N* = 2) were performed. Data were obtained after 7 days of differentiation into dopaminergic- and cholinergic-like neuronal cells

Based on our observations of extensive neurite outgrowth following SH-SY5Y cell transfection and differentiation, we further investigated whether γ-enolase plays differential roles in cytoskeletal organization and neurite outgrowth across different neuronal subtypes. To address this, we analyzed the expression of β-tubulin, a marker of neuronal structural differentiation. Western blot analysis demonstrated increased β-tubulin expression in differentiated compared to non-differentiated wild type SH-SY5Y cells, with the most prominent effect in cholinergic-like neuronal cells (Fig. 3B). The increase in β-tubulin expression was less pronounced in adrenergic-like neuronal cells (Suppl. Fig. S7B). Moreover, cell transfection with both the full-length and ΔC γ-enolase plasmids resulted in increased β-tubulin expression compared to non-differentiated wild type cells; however, the effect was more pronounced in cells expressing full-length γ-enolase. Interestingly, in dopaminergic- or cholinergic-like neuronal cells, transfection with either the total γ-enolase or ΔC γ-enolase plasmids did not increase β-tubulin expression (Fig. 3B). In adrenergic-like neuronal cells, transfection with the ΔC γ-enolase plasmid consistently decreased β-tubulin expression (Suppl. Fig. S7B), indicating that the C-terminal region of γ-enolase may be essential for cytoskeletal differentiation. Nevertheless, immunofluorescence staining of differentiated cells expressing full-length γ-enolase revealed a strong fluorescence signal for β-tubulin in the cell bodies and processes, highlighting the cytoskeletal structures, which was significantly higher than the signal in cells expressing truncated γ-enolase (Fig. 4). The latter expression pattern suggests a role of intact γ-enolase in cytoskeletal organization, with microtubules extending into neurites, which is typical for neuronal differentiation.

### Silencing of γ-Enolase Expression Impairs Neurite Outgrowth in Differentiated Neuro-2a and LA-N-2 Cells

**Fig. 5:**
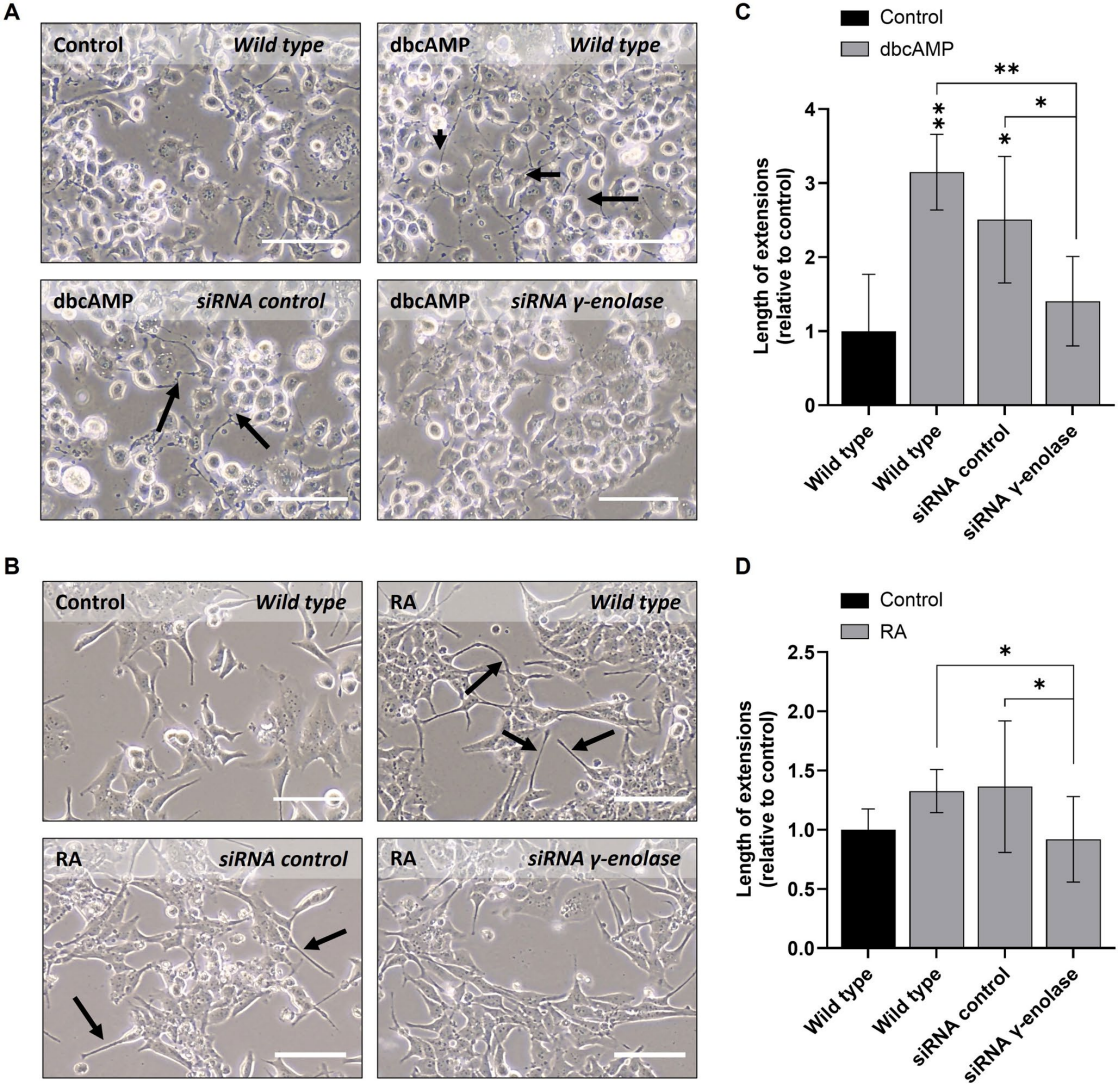
Morphological changes after γ-enolase silencing in differentiated Neuro-2a and LA-N-2 neuronal subtypes. (**A**,** B**) Representative phase-contrast images of differentiated Neuro-2a (**A**) and LA-N-2 (**B**) cells transfected with siRNA γ-enolase. Scale bars: 100 μm. Black arrows indicate cell extensions. (**C**,** D)** Neurite lengths in differentiated Neuro-2a (**C**) and LA-N-2 (**D**) cells transfected with siRNA γ-enolase were determined in pixels when the extensions were longer than the cell diameter using ImageJ software. Data are shown as mean ± 95% CI from *N* = 2 independent experiments each in duplicate. Statistical significance was assessed by one-way ANOVA followed by Tukey’s post hoc test. Exact p-values: (**C**) Control/Wild type vs. dbcAMP/Wild type, *p* = 0.004; dbcAMP/Wild type vs. dbcAMP/siRNA control, *p* = 0.203; dbcAMP/Wild type vs. dbcAMP/siRNA γ-enolase, *p* = 0.009; dbcAMP/siRNA control vs. dbcAMP/siRNA γ-enolase, *p* = 0.044; (**D**) Control/Wild type vs. RA/Wild type, *p* = 0.072; RA/Wild type vs. RA/siRNA control, *p* = 0.900; RA/Wild type vs. RA/siRNA γ-enolase, *p* = 0.065; RA/siRNA control vs. RA/siRNA γ-enolase, *p* = 0.026. Data were obtained after 4 days of differentiation into dopaminergic-like neuronal cells (Neuro-2a), and cholinergic-like neuronal cells (LA-N-2), and are expressed relative to the control

To investigate whether endogenous γ-enolase is required for neuronal differentiation, we silenced γ-enolase expression in Neuro-2a and LA-N-2 cells using siRNA and differentiated the cells into dopaminergic-like (dbcAMP-treated Neuro-2a) and cholinergic-like (RA-treated LA-N-2) neuronal subtypes, respectively. Non-transfected (Wild type) and non-targeting siRNA-transfected (siRNA control) cells served as controls under identical differentiation conditions. Western blot analysis confirmed successful γ-enolase silencing in both neuronal cell lines (Supplementary Fig. S8). In differentiated Neuro-2a and LA-N-2 cells, γ-enolase protein levels were comparable between wild type and control siRNA groups, whereas γ-enolase levels were strongly reduced in cells transfected with γ-enolase-specific siRNA. Morphological analysis revealed that dbcAMP-treated wild type and control siRNA-transfected Neuro-2a cells developed numerous short neuritic processes, forming early neurite networks (Fig. 5A). In contrast, Neuro-2a cells transfected with γ-enolase siRNA showed a noticeable reduction in neurite outgrowth, as observed by cells remaining more rounded, with fewer and shorter processes. Semi-quantitative assessment of neurite length confirmed that γ-enolase silencing attenuated dbcAMP-induced neurite elongation compared to both wild type and siRNA control conditions (Fig. 5C). A similar effect was observed in RA-differentiated LA-N-2 cells. RA-treated wild type LA-N-2 cells developed a characteristic cholinergic-like neuronal morphology, with elongated, thin neurites extending from cell bodies (Fig. 5B). Cells transfected with control siRNA displayed extensive neurite outgrowth comparable to RA-treated wild type cells, again indicating that the transfection procedure did not impair differentiation. In contrast, RA-treated LA-N-2 cells transfected with γ-enolase siRNA exhibited markedly reduced neurite length. These cells displayed only short, poorly branched processes, and the typical long, thin neurites characteristic of RA-differentiated cholinergic-like cells were largely absent. Correspondingly, semi-quantitative analysis showed a reduction in average neurite length upon γ-enolase silencing, bringing values closer to those of non-differentiated controls (Fig. 5D).

### γ-Enolase–derived Peptide Enhances Neurite Outgrowth and Differentiation Specifically in Cholinergic-like SH-SY5Y Cells

**Fig. 6:**
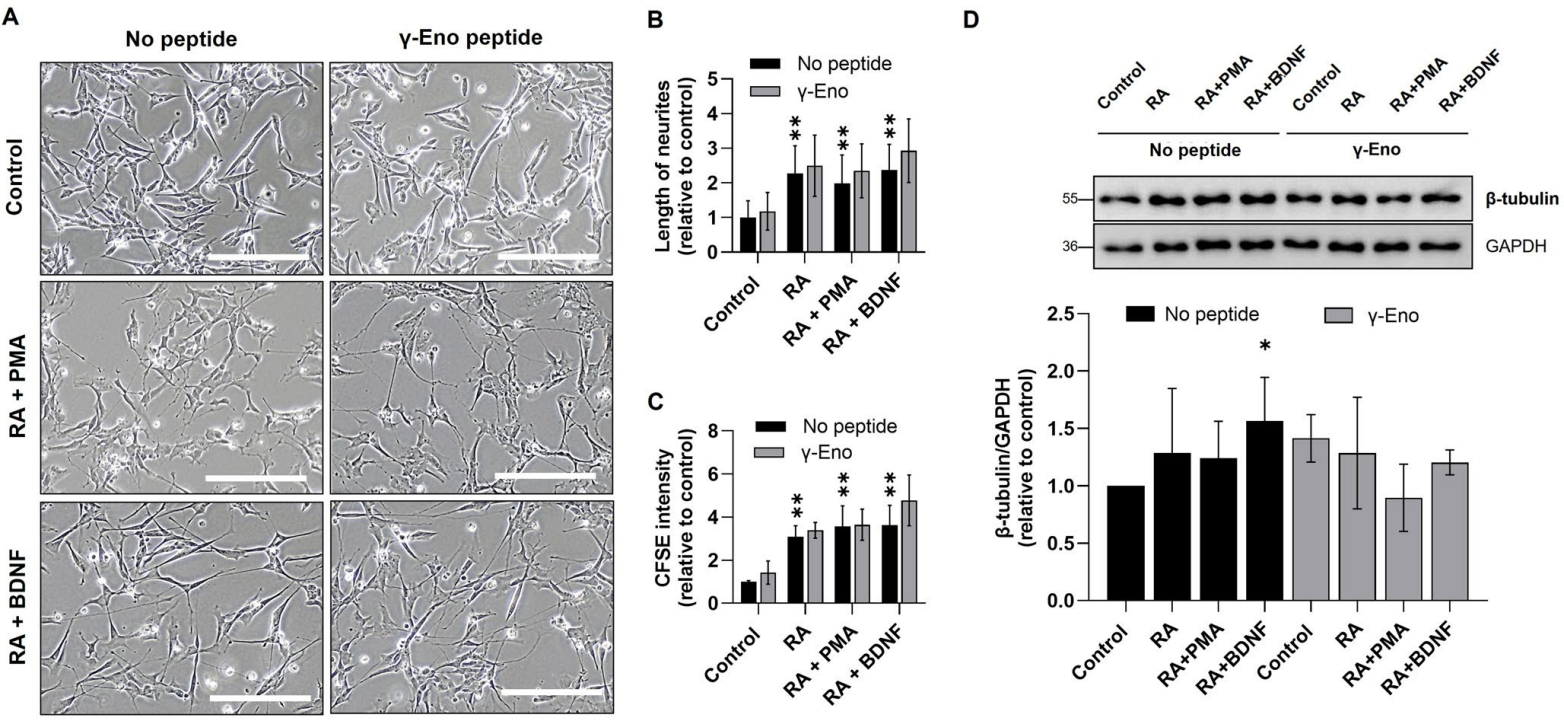
The effects of γ-enolase peptide treatment on cell morphology, proliferation, and β-tubulin expression in differentiated SH-SY5Y neuronal subtypes. (**A**) Representative phase-contrast images of SH-SY5Y cells treated with γ-enolase peptide corresponding to the last 30 amino acids of the protein (γ-Eno). Scale bars: 100 μm. (**B)** Neurite lengths in dopaminergic- and cholinergic-like neuronal cells treated with γ-Eno were determined in pixels when the extensions were longer than the cell diameter using ImageJ software. Data are shown as mean ± 95% CI from *N* = 2 independent experiments each in duplicate. Statistical significance was assessed by one-way ANOVA followed by Tukey’s post hoc test. Exact p-values: Control/No peptide vs. RA/No peptide, *p* = 0.001; Control/No peptide vs. RA + PMA/No peptide, *p* = 0.002; Control/No peptide vs. RA + BDNF/No peptide, *p* = 0.006; Control/No peptide vs. Control/γ-Eno peptide, *p* = 0.675; RA/No peptide vs. RA/γ-Eno peptide, *p* = 0.418; RA + PMA/No peptide vs. RA + PMA/γ-Eno peptide, *p* = 0.149; RA + BDNF/No peptide vs. RA + BDNF/γ-Eno peptide, *p* = 0.161. (**C**) The proliferation rates of dopaminergic- and cholinergic-like neuronal cells treated with γ-Eno were assessed with carboxyfluorescein succinimidyl ester (CFSE) labeling and flow cytometry. Data are shown as mean ± 95% CI from *N* = 2 independent experiments, each in duplicate. Statistical significance was assessed by one-way ANOVA followed by Tukey’s post hoc test. Exact p-values: Control/No peptide vs. RA/No peptide, *p* = 0.001; Control/No peptide vs. RA + PMA/No peptide, *p* = 0.001; Control/No peptide vs. RA + BDNF/No peptide, *p* = 0.005 Control/No peptide vs. Control/γ-Eno peptide, *p* = 0.680; RA/No peptide vs. RA/γ-Eno peptide, *p* = 0.769; RA + PMA/No peptide vs. RA + PMA/γ-Eno peptide, *p* = 0.900; RA + BDNF/No peptide vs. RA + BDNF/γ-Eno peptide, *p* = 0.020. (**D**) Representative western blots (top) and quantification (bottom) of the expression of β-tubulin. Protein levels are normalized to GAPDH and expressed relative to the control. Data are shown as mean ± 95% CI from *N* = 2 independent experiments. Statistical significance was assessed by one-way ANOVA followed by Tukey’s post hoc test. Exact p-values: Control/No peptide vs. RA/No peptide, *p* = 0.430; Control/No peptide vs. RA + PMA/No peptide, *p* = 0.547; Control/No peptide vs. RA + BDNF/No peptide, *p* = 0.049 Control/No peptide vs. Control/γ-Eno peptide, *p* = 0.500; RA/No peptide vs. RA/γ-Eno peptide, *p* = 0.900; RA + PMA/No peptide vs. RA + PMA/γ-Eno peptide, *p* = 0.157; RA + BDNF/No peptide vs. RA + BDNF/γ-Eno peptide, *p* = 0.051. Data were obtained after 7 days of differentiation into dopaminergic- and cholinergic-like neuronal cells and are expressed relative to the control

The role of γ-enolase was additionally evaluated by applying a synthetic peptide (γ-Eno) corresponding to the C-terminal 30-amino-acid sequence of human brain γ-enolase. Treatment with γ-Eno affected neurite outgrowth in all SH-SY5Y cell subtypes (Fig. 6A), with the most pronounced effects observed in cholinergic-like neuronal cells, which exhibited enhanced neurite elongation compared to untreated controls and dopaminergic-like neuronal cells (Fig. 6B and D). Additionally, this subtype demonstrated the highest increase in CFSE levels, indicating that γ-Eno enhances cell differentiation (Fig. 6C and E). By contrast, dopaminergic-like neuronal cells showed moderate neurite outgrowth with no significant changes in proliferation. Similarly, adrenergic-like neuronal cells exposed to γ-Eno peptide showed no significant changes in neurite outgrowth or cell proliferation (Suppl. Fig. S9). These findings suggest that γ-enolase plays a role in promoting both structural and proliferative differentiation, particularly in cholinergic-like neuronal cells. In addition, western blot analysis of β-tubulin (Fig. 6D) revealed distinct effects of γ-Eno depending on the differentiation state of SH-SY5Y cells. In non-differentiated cells, γ-Eno significantly increased β-tubulin expression, suggesting its role in promoting cytoskeletal organization and initiating structural neuronal features in non-differentiated cells. By contrast, β-tubulin expression decreased in both dopaminergic- and cholinergic-like differentiated cells after treatment, suggesting that γ-enolase may have a limited effect on cytoskeletal marker expression once cells have undergone differentiation. These results suggest that γ-Eno might play a specific role in modulating cytoskeletal elements in a differentiation-dependent manner.

### Cathepsin X Regulates γ-Enolase Expression in SH-SY5Y Cells Differentiated into Specific Subtypes

**Fig. 7:**
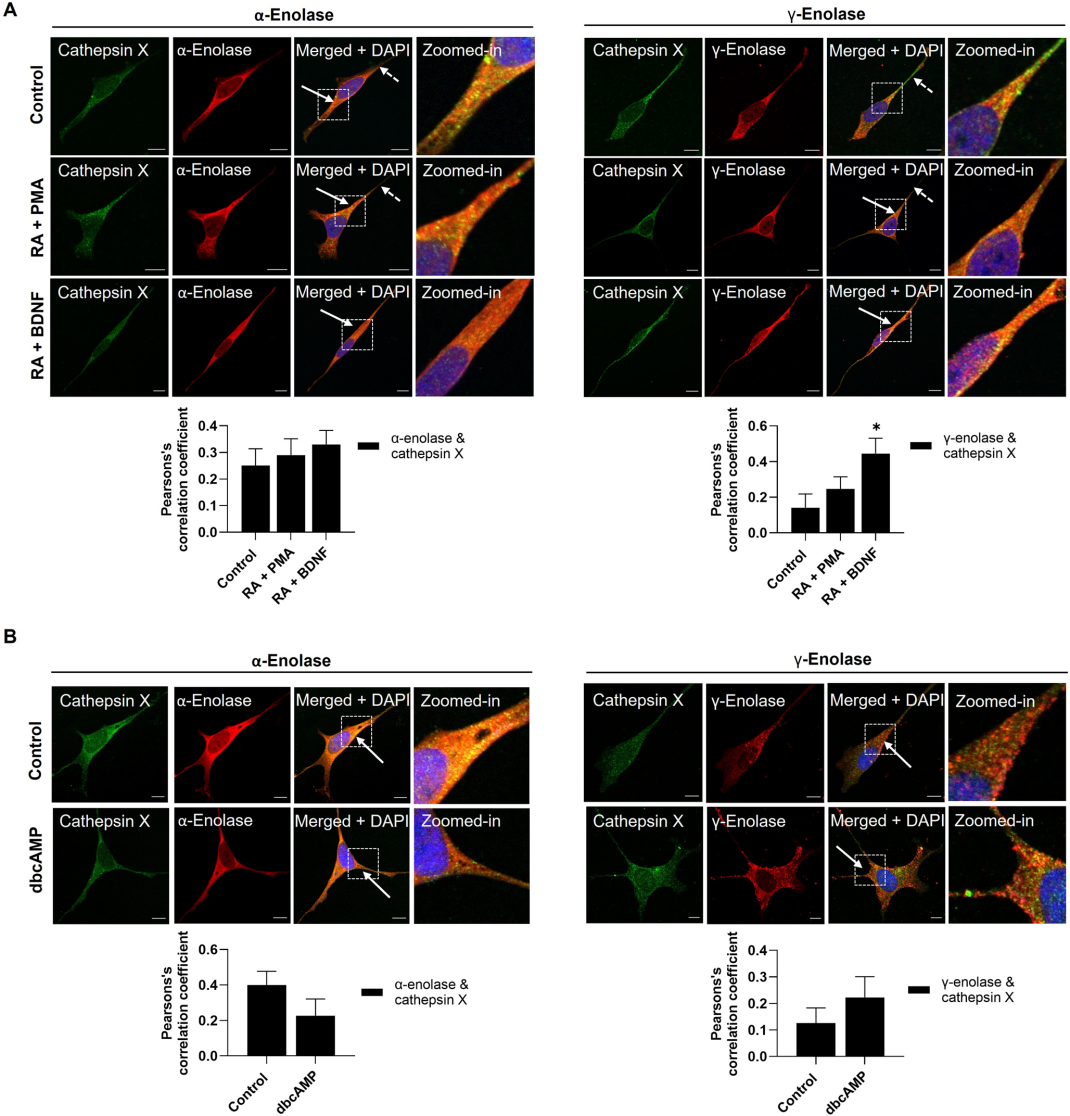
Cathepsin X co-localization with enolase isoforms in differentiated SH-SY5Y neuronal subtypes. (**A**,** B**) Representative images of immunofluorescence staining for cathepsin X (green) and α-enolase (red, left), and γ-enolase (red, right) for (**A**) dopaminergic-, cholinergic-, and (**B**) adrenergic-like neuronal cells. Nuclei were counterstained with DAPI (blue). White solid arrows indicate areas with strong co-localization. White dashed arrows indicate areas on neurites with low co-localization. Scale bars: 10 μm. The graphs below show Pearson’s correlation coefficients. Data are shown as mean ± 95% CI from *N* = 2 independent experiments. Statistical significance was assessed by one-way ANOVA. Exact p-values: (**A**) α-enolase & cathepsin X - not significant, *p* = 0.661; γ-enolase & cathepsin X - Control vs. RA + PMA, *p* = 0.181; Control vs. RA + BDNF, *p* = 0.002; RA + PMA vs. RA + BDNF, *p* = 0.015; (**B**) α-enolase & cathepsin X Control vs. dbcAMP, *p* = 0.042; γ-enolase & cathepsin X - Control vs. dbcAMP, *p* = 0.208. Data were obtained after 7 days of differentiation into dopaminergic- and cholinergic-like neuronal cells and after 4 days of differentiation into adrenergic-like neuronal cells and are expressed relative to the control

Due to the functional role of γ-enolase in differentiated cells, we were interested in the regulation of its neurotrophic activity by the carboxypeptidase cathepsin X. To explore this, we first evaluated the cellular co-localization of both proteins in differentiated SH-SY5Y cells. As revealed by confocal microscopy, the co-localization of α-enolase and cathepsin X was unchanged in differentiated dopaminergic- and cholinergic-like neuronal cells (Fig. 7A), but lower in adrenergic-like neuronal cells; however, the difference was not significant (Fig. 7B). By contrast, the co-localization of γ-enolase and cathepsin X was increased in all differentiated SH-SY5Y subtypes compared to non-differentiated cells, significantly in cholinergic-like neuronal cells (Fig. 7A and B). Of note, the most significant co-localization was observed in cholinergic-like neuronal cells, suggesting an important interaction between γ-enolase and cathepsin X in this neuronal subtype. We further assessed cathepsin X expression and activity in differentiated SH-SY5Y cells (Suppl. Fig. S10). ELISA revealed increased cathepsin X levels in dopaminergic-like neuronal cells compared to non-differentiated controls and cholinergic-like neuronal cells. Furthermore, enzyme activity assays revealed increased cathepsin X activity in dopaminergic-like neuronal cells compared to cholinergic-like neuronal cells. Conversely, adrenergic-like neuronal cells exhibited slightly increased expression but decreased enzyme activity of cathepsin X (Suppl. Fig. S10).

We further validated our findings in the Neuro-2a and LA-N-2 cell lines differentiated into dopaminergic- and cholinergic-like neuronal subtypes. Similar co-localization patterns of cathepsin X with enolase isoforms were observed in these differentiated cells as in differentiated SH-SY5Y cells. Compared to the corresponding non-differentiated cells, dopaminergic-like Neuro-2a and cholinergic-like LA-N-2 cells exhibited lower co-localization of α-enolase and cathepsin X and higher co-localization of γ-enolase and cathepsin X; however, these differences were not significant (Suppl. Fig. S11A, S11B). Further analysis demonstrated increased cathepsin X levels and activity in differentiated Neuro-2a and LA-N-2 cells compared to their non-differentiated controls (Suppl. Figure 11 C–11 F). These results further support the differentiation-dependent interaction between γ-enolase and cathepsin X, consistent with the patterns observed in SH-SY5Y neuronal subtypes.

**Fig. 8:**
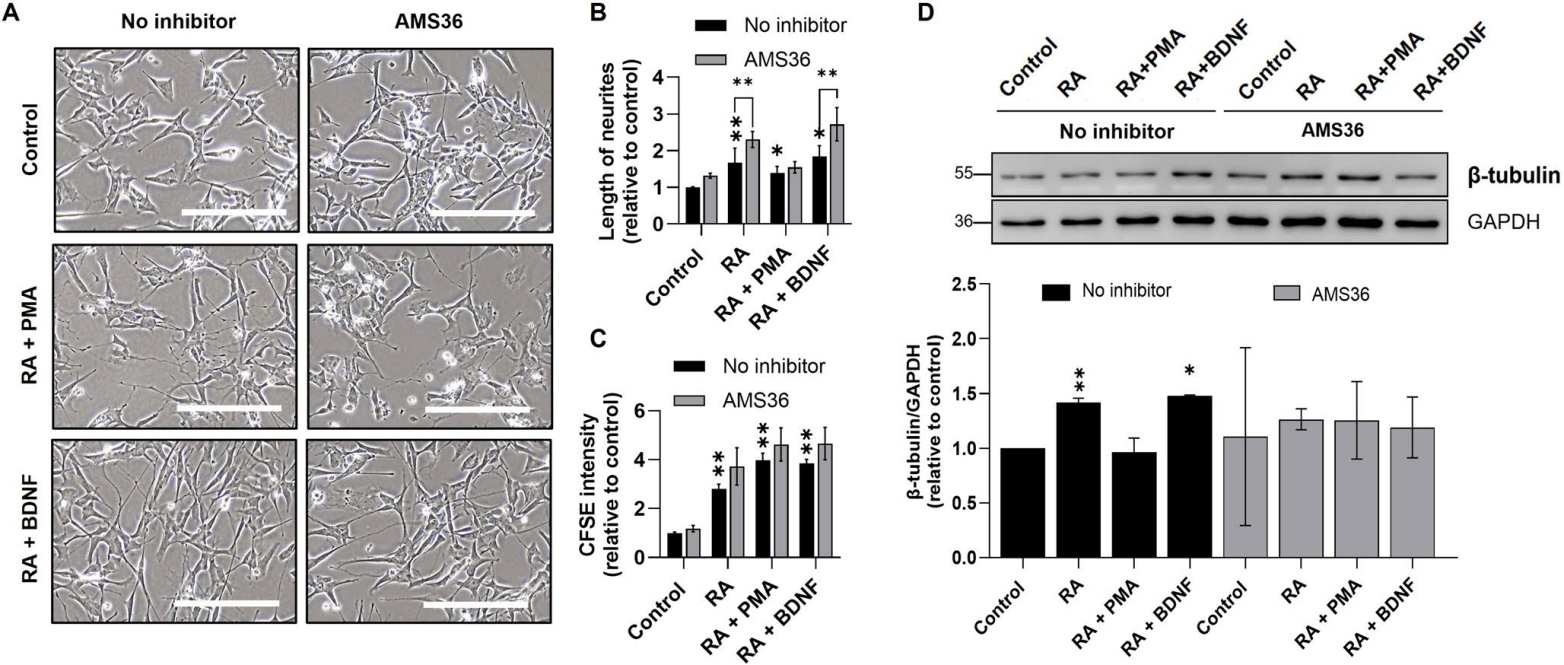
The effects of cathepsin X inhibition on cell morphology, proliferation, and expression of γ-enolase, β-tubulin in differentiated SH-SY5Y neuronal subtypes. (**A**) Representative phase-contrast images of SH-SY5Y cells treated with AMS36. Scale bars: 100 μm. (**B**) Neurite lengths in dopaminergic- and cholinergic-like neuronal cells treated with AMS36 were determined in pixels using ImageJ software when cell extensions were longer than the cell diameter. Data are shown as mean ± 95% CI from *N* = 2 independent experiments, each in duplicate. Statistical significance was assessed by one-way ANOVA followed by Tukey’s post hoc test. Exact p-values: Control/No inhibitor vs. RA/No inhibitor, *p* = 0.003; Control/No inhibitor vs. RA + PMA/No inhibitor, *p* = 0.038; Control/No inhibitor vs. RA + BDNF/No inhibitor, *p* = 0.009; Control/No inhibitor vs. Control/AMS36, *p* = 0.094; RA/No inhibitor vs. RA/AMS36, *p* = 0.004; RA + PMA/No inhibitor vs. RA + PMA/AMS36, *p* = 0.614; RA + BDNF/No inhibitor vs. RA + BDNF/AMS36, *p* = 0.008. (**C**) The proliferation rates of dopaminergic- and cholinergic-like neuronal cells treated with AMS36 were assessed with carboxyfluorescein succinimidyl ester (CFSE) labeling and flow cytometry. Data are shown as mean ± 95% CI from *N* = 3 independent experiments, each in duplicate. Statistical significance was assessed by one-way ANOVA followed by Tukey’s post hoc test. Exact p-values: Control/No inhibitor vs. RA/No inhibitor, *p* = 0.001; Control/No inhibitor vs. RA + PMA/No inhibitor, *p* = 0.001; Control/No inhibitor vs. RA + BDNF/No inhibitor, *p* = 0.001; Control/No inhibitor vs. Control/AMS36, *p* = 0.900; RA/No inhibitor vs. RA/AMS36, *p* = 0.156; RA + PMA/No inhibitor vs. RA + PMA/AMS36, *p* = 0.290; RA + BDNF/No inhibitor vs. RA + BDNF/AMS36, *p* = 0.088. **(D)** Representative western blots (top) and quantification (bottom) of the expression of β-tubulin. Protein levels are normalized to GAPDH and expressed relative to the control. Data are shown as mean ± 95% CI from *N* = 2 independent experiments. Statistical significance was assessed by one-way ANOVA followed by Tukey’s post hoc test. Exact p-values: Control/No inhibitor vs. RA/No inhibitor, *p* = 0.006; Control/No inhibitor vs. RA + PMA/No inhibitor, *p* = 0.900; Control/No inhibitor vs. RA + BDNF/No inhibitor, *p* = 0.032; Control/No inhibitor vs. Control/AMS36, *p* = 0.900; RA/No inhibitor vs. RA/AMS36, *p* = 0.831; RA + PMA/No inhibitor vs. RA + PMA/AMS36, *p* = 0.268; RA + BDNF/No inhibitor vs. RA + BDNF/AMS36, *p* = 0.278. Data were obtained after 7 days of differentiation into dopaminergic- and cholinergic-like neuronal cells and are expressed relative to the control

To explore the functional role of cathepsin X in regulating γ-enolase in differentiated cells, we investigated the effects of AMS36, a cathepsin X inhibitor, on cell differentiation. AMS36 induced morphological changes, including increased neurite length, compared to control cells (Fig. 8A and B). In dopaminergic-like neuronal cells, AMS36 slightly enhanced neurite outgrowth and increased CFSE intensity, compared to control cells (Fig. 8C). In cholinergic-like neuronal cells, AMS36 increased both neurite length and CFSE intensity and thus demonstrated enhanced differentiation (Fig. 8B and C). By contrast, in adrenergic-like neuronal cells, AMS36 did not affect neurite outgrowth or CFSE intensity (Suppl. Fig. S12). These findings suggest that AMS36 enhances neuronal outgrowth and differentiation, particularly in cholinergic-like neuronal cells, underscoring its potential role in regulating γ-enolase activities in specific neuronal subtypes. Indeed, western blot analyses revealed the effects of AMS36 on the expression of the active and total forms of γ-enolase across differentiated neuronal subtypes and non-differentiated SH-SY5Y cells (Suppl. Fig. S13). AMS36 slightly increased the expression of the active form of γ-enolase in differentiated SH-SY5Y cells compared to DMSO-treated cells, whereas the total form remained unchanged. This suggests that AMS36 prevents the cleavage of the C-terminal part of γ-enolase, thereby increasing its active form without altering total γ-enolase expression. Moreover, western blot analysis was performed to evaluate the expression of β-tubulin (Fig. 8D) in differentiated neuronal subtypes treated with AMS36 or vehicle. Compared to vehicle-treated controls, AMS36-treated cells exhibited decreased β-tubulin expression, indicating that AMS36 does not enhance cytoskeletal protein expression. This decrease was consistently observed in both dopaminergic- and cholinergic-like neuronal cells, in which β-tubulin levels remained similar between these subtypes after treatment. By contrast, in adrenergic-like neuronal cells, AMS36 significantly decreased β-tubulin levels compared to vehicle-treated controls (Fig. S12D). Collectively, these results suggest that cathepsin X inhibition by AMS36 enhances γ-enolase activity, thereby promoting neurite outgrowth and differentiation, particularly in cholinergic-like neuronal cells, while differentially modulating cytoskeletal markers across all neuronal subtypes.

## Discussion

The present study has investigated the role of γ-enolase in neuronal cells differentiated into specific subtypes. The optimization of SH-SY5Y cell differentiation into dopaminergic-, cholinergic-, and adrenergic-like neuronal subtypes enabled us to analyze the mechanisms and regulation of γ-enolase action. Significant neurite outgrowth was observed particularly in cholinergic-like neuronal cells, consistent with the role of BDNF in promoting neurite elongation in SH-SY5Y cells (Medeiros et al., 2019; Targett et al., 2024; Hromadkova et al., 2020; Encinas et al., 2000). By contrast, dopaminergic- and adrenergic-like neuronal cells developed a branched morphology with new bud formations, as reported in similar studies (Kume et al., 2008; Pifferi et al., 2024). Furthermore, the significant inhibition of cell proliferation, indicated by increased CFSE levels, confirms the differentiated status of neuronal cells in this study (Pifferi et al., 2024; Cooper, 2000). Immunostaining, western blot, and flow cytometry analyses validated subtype-specific differentiation. Dopaminergic-like neuronal cells showed increased TH levels (Presgraves et al., 2003; Xie et al., 2010); cholinergic-like neuronal cells increased ChAT and acetylcholinesterase levels (Medeiros et al., 2019); and adrenergic-like neuronal cells upregulated ADRA2A levels (Kovalevich & Langford, 2013). Similar validation of Neuro-2a and LA-N-2 cells through TH and acetylcholinesterase expression confirmed the reliability of our differentiation protocols for functional experiments (Tremblay et al., 2010; Singh et al., 1990).

The most significant finding was that enolase isoforms exhibited differential expression patterns across neuronal subtypes, advancing previous research that had identified γ-enolase as a general marker of mature neurons (Schmechel et al., 1980). Cholinergic-like neuronal cells exhibited significantly higher γ-enolase expression than dopaminergic- and adrenergic-like neuronal cells and non-differentiated cells. Additionally, the increased active-to-total γ-enolase ratio in cholinergic-like neuronal cells suggests a more prominent role for γ-enolase in this neuronal subtype.

Previous research has shown that neurotrophic factors promote cholinergic differentiation in neurons derived from human embryonic stem cells (Nilbratt et al., 2010). This suggests that γ-enolase, a neurotrophic-like factor (Hattori et al., 1995), may play a similar role in supporting cholinergic differentiation and maturation. Moreover, studies have identified the tropomyosin receptor kinase (Trk) family as a critical mediator of neurotrophic signaling, with γ-enolase potentially acting as a binding partner (Pišlar & Kos, 2021). Our results of increased γ-enolase expression following RA and BDNF treatment are consistent with the role of BDNF in neuronal development via TrkB activation (Lewin & Carter, 2014), suggesting that TrkB signaling may regulate γ-enolase activity, enhancing its neuroprotective and neurotrophic effects. Furthermore, studies have shown that RA increases TrkB expression in SH-SY5Y cells, making them more sensitive to stimulation by BDNF (Kaplan et al., 1993; Edsjö et al., 2003). BDNF activates TrkB, which promotes neuronal differentiation and survival. This suggests a potential link between TrkB activation and increased γ-enolase expression, which may explain the neurotrophic role of γ-enolase in cholinergic neurons. Additionally, adrenergic-like neuronal cells also exhibited increased γ-enolase expression, suggesting that dbcAMP promotes neuronal differentiation through protein kinase A signaling, which is known to regulate cytoskeletal rearrangements and neurite outgrowth (Aglah et al., 2008). This finding indicates that γ-enolase may be involved in multiple signaling pathways, depending on the neuronal subtype. Moreover, the expression of α-enolase remained consistent or decreased in cholinergic- and adrenergic-like neuronal cells, emphasizing the distinct expression patterns between α- and γ-enolase. Previous studies have shown that γ-enolase is more closely associated with neuronal differentiation and survival, whereas α-enolase plays a broader role in metabolic processes (Fletcher et al., 1976; Schmechel et al., 1980). The differential expression of these isoforms further suggests that γ-enolase may be a critical factor in the differentiation and maintenance of neuronal subtypes, whereas α-enolase serves more general cellular functions.

The role of γ-enolase in neuronal differentiation was further explored by overexpressing γ-enolase in the different neuronal subtypes. Overexpression of γ-enolase significantly enhanced neurite outgrowth, especially in cholinergic-like neuronal cells. This aligns with previous findings that γ-enolase promotes neurite outgrowth in non-differentiated SH-SY5Y cells (Pišlar & Kos, 2021), extending its role to differentiated subtypes. Moreover, differentiated cells transfected with the truncated ΔC γ-enolase plasmid exhibited slightly shorter neurites compared to cells transfected with the total γ-enolase plasmid, suggesting that the C-terminal region of γ-enolase is important in promoting neurite outgrowth. This finding aligns with previous research demonstrating that the C-terminal region of γ-enolase enhances cell survival and neurite outgrowth by activating the PI3K/Akt and MAPK/ERK signaling pathways (Hattori et al., 1995; Hafner et al., 2012).

The analysis of β-tubulin expression revealed a differentiation-dependent role of γ-enolase in cytoskeletal organization. The higher β-tubulin expression in wild type cholinergic-like neuronal cells, compared to dopaminergic- and adrenergic-like neuronal cells, highlights subtype-specific differences in cytoskeletal dynamics. These findings align with previous research showing that β-tubulin upregulation is closely associated with neurite outgrowth and cytoskeletal reorganization during neuronal differentiation (Encinas et al., 2000). Interestingly, transfection with total γ-enolase or ΔC γ-enolase did not upregulate β-tubulin in differentiated dopaminergic- or cholinergic-like neuronal cells; therefore, the effect of γ-enolase on cytoskeletal organization may be attenuated once differentiation is established. This was confirmed in non-differentiated cells, in which both γ-enolase constructs increased β-tubulin expression, indicating a key role of γ-enolase in initiating cytoskeletal assembly and structural neuronal features at earlier differentiation stages. The lower β-tubulin levels observed in cells transfected with the ΔC γ-enolase plasmid, particularly in adrenergic-like neuronal cells, suggest that the C-terminal region of γ-enolase plays a role in cytoskeletal organization, consistent with its role in neurite extension and cytoskeletal remodeling (Hafner et al., 2012). Silencing of γ-enolase further supported its role in neurite outgrowth and subtype-specific differentiation. Attempts to silence γ-enolase in differentiated SH-SY5Y cells using siRNA were not successful; transfection efficiency was low, and the degree of silencing varied considerably between days 5 and 7 of differentiation. Consequently, loss-of-function studies were performed in Neuro-2a and LA-N-2 cells, which can be robustly differentiated into dopaminergic- and cholinergic-like neuronal subtypes in 3 days, respectively. In these models, γ-enolase silencing produced the opposite phenotype to that observed with γ-enolase overexpression. In dbcAMP-differentiated Neuro-2a cells, siRNA-mediated silencing of γ-enolase resulted in a reduction of neurite number and length, with cells retaining a more rounded morphology and forming only a few neuritic connections, whereas wild type and non-targeting siRNA controls exhibited clear neurite extension. This inhibitory effect on neurite outgrowth was observed in RA-differentiated LA-N-2 cells as well, in which γ-enolase silencing disrupted the typical cholinergic-like morphology characterized by long and thin neurites. Together, the overexpression and silencing data indicate that γ-enolase plays a key role in neurite elongation and cytoskeletal organization.

The role of γ-enolase in differentiated neuronal subtypes and cytoskeletal organization was further elucidated by treatment with γ-Eno, a synthetic peptide corresponding to the C-terminal 30-amino acid sequence of γ-enolase. The obtained results align with the transfection experiments, as γ-Eno similarly promoted neurite elongation and increased CFSE levels in cholinergic-like neuronal cells, indicating enhanced differentiation. This is consistent with prior findings demonstrating that the C-terminal region of γ-enolase possesses neurotrophic properties (Hafner et al., 2012; Hattori et al., 1994). Both full-length γ-enolase and the γ-Eno peptide increased β-tubulin expression in non-differentiated cells. However, this effect diminished in differentiated dopaminergic- and cholinergic-like neuronal cells, in which decreased β-tubulin expression after γ-Eno treatment suggests stabilization of the cytoskeletal framework during differentiation. The stabilization of β-tubulin in differentiated neurons aligns with evidence from previous studies demonstrating that microtubules undergo post-translational modifications, such as acetylation and polyglutamylation, during neuronal maturation (Audebert et al., 1994). These modifications enhance microtubule stability and reduce the need for dynamic tubulin turnover, which may explain the diminished effects of γ-enolase transfection and γ-Eno on β-tubulin expression in mature neurons. Importantly, cross-linking of γ-enolase peptide to microspheres, preventing its internalization, reduced the peptide effect significantly (Hafner et al., 2012). Moreover, full neurotrophic activity requires γ-enolase to localize to the cell surface, a process facilitated by its PDZ-binding motif at the C-terminus and the scaffold protein γ1-syntrophin that recruits γ-enolase to the plasma membrane (Hafner et al., 2010; Pišlar & Kos, 2021). Previous studies have clearly demonstrated that a synthetic peptide corresponding to the C-terminal 30 amino acids of γ-enolase is sufficient to drive neuronal survival, promote differentiation, and stimulate neurite outgrowth, effectively mimicking the neurotrophic functions of the full-length protein (Hafner et al., 2012; Hattori et al., 1994; Obermajer et al., 2009). Moreover, BDNF has been shown to play a critical role in regulating dendritic spine morphology and synaptic structure in mature neurons, with its effects being activity-dependent and focused on stabilizing neuronal architecture rather than driving cytoskeletal remodeling (Kellner et al., 2014). The diminished effect of γ-enolase on β-tubulin expression in differentiated neurons may reflect a parallel role, in which neurotrophic-like factors transition from facilitating dynamic growth to maintaining structural stability. It appears that γ-enolase plays a role in non-differentiated neurons by regulating cytoskeletal components and thereby promoting neurite growth. However, as neurons undergo differentiation and cytoskeletal stabilization, its influence diminishes, reflecting a functional shift similar to the activity-dependent effects of BDNF in mature neurons (Kellner et al., 2014). Overall, these results highlight the critical role of the C-terminal region of γ-enolase in promoting neurite outgrowth and cytoskeletal differentiation, especially in non-differentiated cells and cholinergic-like neuronal cells.

By analyzing the co-localization of cathepsin X with enolase isoforms and its expression and activity across neuronal subtypes, we identified a functional relationship highlighting the role of these proteins in regulating neuronal differentiation and structural plasticity. Cathepsin X, a cysteine peptidase, is known to regulate neuronal survival and neuritogenesis by cleaving the C-terminal amino acids of both γ- and α-enolase (Obermajer et al., 2009; Kos et al., 2009). The co-localization of cathepsin X and γ-enolase was increased in all differentiated SH-SY5Y cells, particularly in cholinergic-like neuronal cells. By contrast, α-enolase exhibited decreased co-localization with cathepsin X in adrenergic-like neuronal cells, indicating a diminished functional interaction in this subtype. This finding may indicate that the role of α-enolase in adrenergic-like neuronal cells is more closely tied to its glycolytic activity than to neurotrophic functions modulated by cathepsin X. Cathepsin X exhibited increased activity in dopaminergic-like neuronal cells, and its subtype-specific activity highlights its differential roles in neuronal differentiation.

Inhibition of cathepsin X with AMS36 revealed the functional significance of cathepsin X in promoting differentiation and neurite outgrowth, particularly in cholinergic-like neuronal cells. Previous research demonstrated a protective role of cathepsin X against 6-hydroxydopamine-induced oxidative cytotoxicity in non-differentiated SH-SY5Y cells (Pišlar et al., 2014). Our current study demonstrated that AMS36 significantly increased neurite lengths and CFSE levels in cholinergic-like neuronal cells. AMS36 also decreased β-tubulin expression, particularly in dopaminergic- and adrenergic-like neuronal cells, further illustrating the differentiation-dependent role of cathepsin X and suggesting that cathepsin X inhibition does not directly enhance cytoskeletal protein expression. In cholinergic-like neuronal cells β-tubulin expression was slightly decreased, indicating that AMS36 primarily influences γ-enolase-mediated processes rather than directly modulating cytoskeletal processes. These findings suggest that AMS36-induced cathepsin X inhibition increases the level of the active part of γ-enolase and thus supports neurite outgrowth and differentiation, particularly in cholinergic-like neuronal cells. The differential effects across subtypes highlight the complexity of the interactions between cathepsin X and γ-enolase, with limited effects on cytoskeletal markers, and warrant further research.

## Conclusion

This study advances our understanding of the role of γ-enolase in specific neuronal subtypes, particularly in cholinergic-like neuronal cells, in which it promotes neurite outgrowth. Targeting regulatory pathways involving γ-enolase and cathepsin X (e.g., by using γ-enolase mimetic peptides or cathepsin X inhibitors) shows potential for enhancing neuronal repair and differentiation, potentially complementing existing neurotrophic treatments for neurodegenerative diseases. Although primary neuronal models were not employed, the neuronal differentiation patterns observed in our in vitro systems align with previously published data, supporting the robustness and translational value of our findings. Collectively, these results establish a mechanistic and functional framework for developing γ-enolase–targeted interventions for neurodegenerative disorders.

## Supplementary Information

Below is the link to the electronic supplementary material.

Supplementary Material 1

Supplementary Material 2

Supplementary Material 3

## Data Availability

The datasets generated and/or analyzed during the current study are available from the corresponding author upon reasonable request.
