# Supplementary material for: Neuronal Subtype-Specific Expression of γ-Enolase: Its Role in Neuronal Differentiation
Source: Neuromolecular Med. 2026 Jan 30;28(1):4. doi: 10.1007/s12017-025-08902-9 (PMC12858592; doi:10.1007/s12017-025-08902-9)
Supplement: Supplementary file 1 — Supplementary Material 1 [file 12017_2025_8902_MOESM1_ESM.docx]

**Neuronal Subtype-Specific Expression of γ-Enolase: Its Role in Neuronal Differentiation**

Selena Horvat ([selena.horvat@ffa.uni-lj.si](mailto:selena.horvat@ffa.uni-lj.si))^a^, Urša Pečar Fonović ([ursa.pecarfonovic@ffa.uni-lj.si](mailto:ursa.pecarfonovic@ffa.uni-lj.si))^a^, Nace Zidar ([nace.zidar@ffa.uni-lj.si](mailto:nace.zidar@ffa.uni-lj.si))^b^, Bojan Doljak ([bojan.doljak@ffa.uni-lj.si](mailto:bojan.doljak@ffa.uni-lj.si))^a^, Janko Kos ([janko.kos@ffa.uni-lj.si](mailto:janko.kos@ffa.uni-lj.si))^a,c^, Anja Pišlar ([anja.pislar@ffa.uni-lj.si](mailto:anja.pislar@ffa.uni-lj.si))^a,*^

^a^ Department of Pharmaceutical Biology, Faculty of Pharmacy, University of Ljubljana, Aškerčeva 7, 1000 Ljubljana, Slovenia

^b^ Department of Pharmaceutical Chemistry, Faculty of Pharmacy, University of Ljubljana, Aškerčeva 7, 1000 Ljubljana, Slovenia

^c^ Department of Biotechnology, Jožef Stefan Institute, Jamova 39, 1000 Ljubljana, Slovenia

***Correspondence: Anja Pišlar**

Department of Pharmaceutical Biology, Faculty of Pharmacy, University of Ljubljana, Aškerčeva 7, 1000 Ljubljana, Slovenia; Tel: +386-1-4769526; Fax: +386-1-4258031; E-mail: [anja.pislar@ffa.uni-lj.si](mailto:anja.pislar@ffa.uni-lj.si); ORCID iD: 0000-0002-1159-1024.

**Supplementary: Original Western Blots**

**
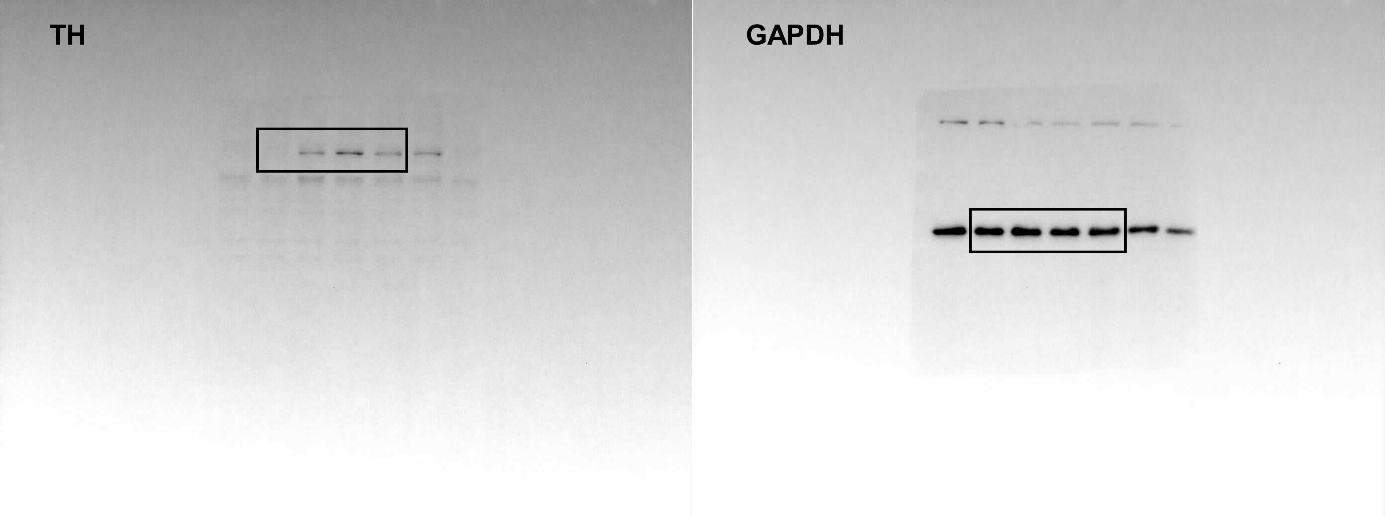
**

**Addition to Fig. 1M:** Raw images of the representative western-blotted membrane of the expression of tyrosine (TH) with the appropriate representative western-blotted membrane of the expression of GAPDH**.**

**
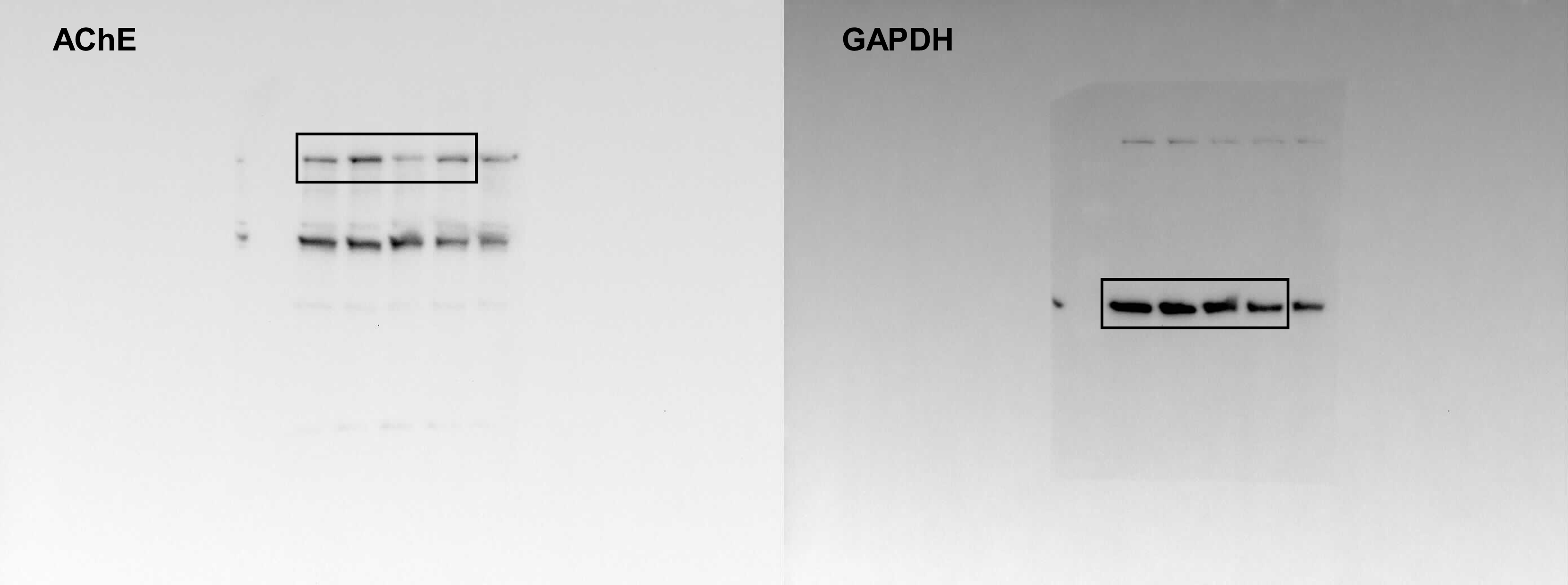
**

**Addition to Fig. 1N:** Raw images of the representative western-blotted membrane of the expression of acetylcholinesterase (AChE) with the appropriate representative western-blotted membrane of the expression of GAPDH**.**

**
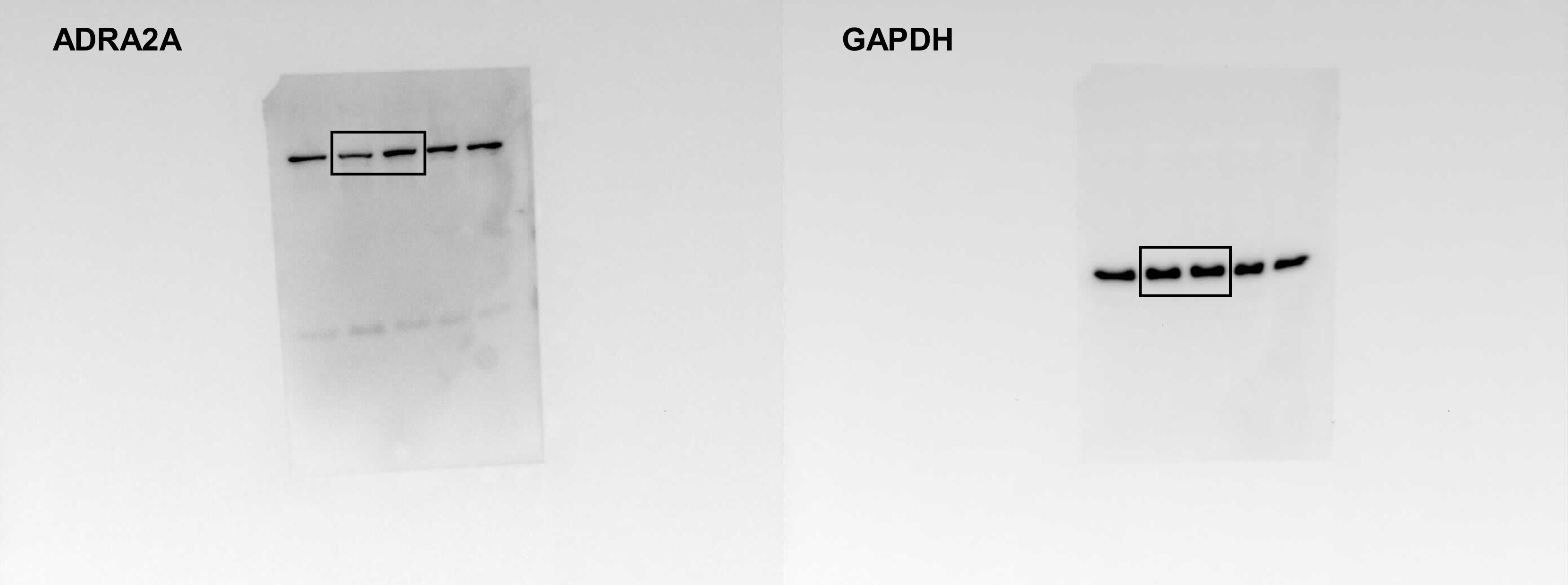
**

**Addition to Fig. 1O:** Raw images of the representative western-blotted membrane of the expression of α-2 adrenergic receptor (ADRA2A) with the appropriate representative western-blotted membrane of the expression of GAPDH**.**

**
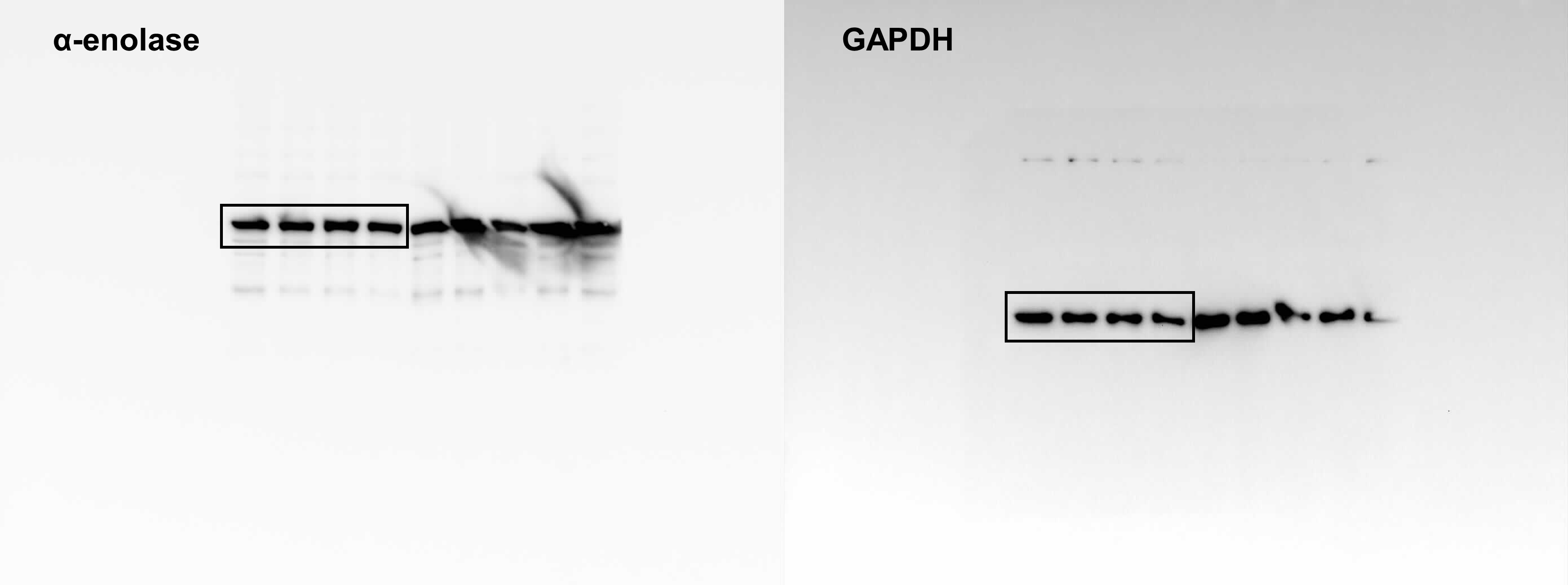
**

**
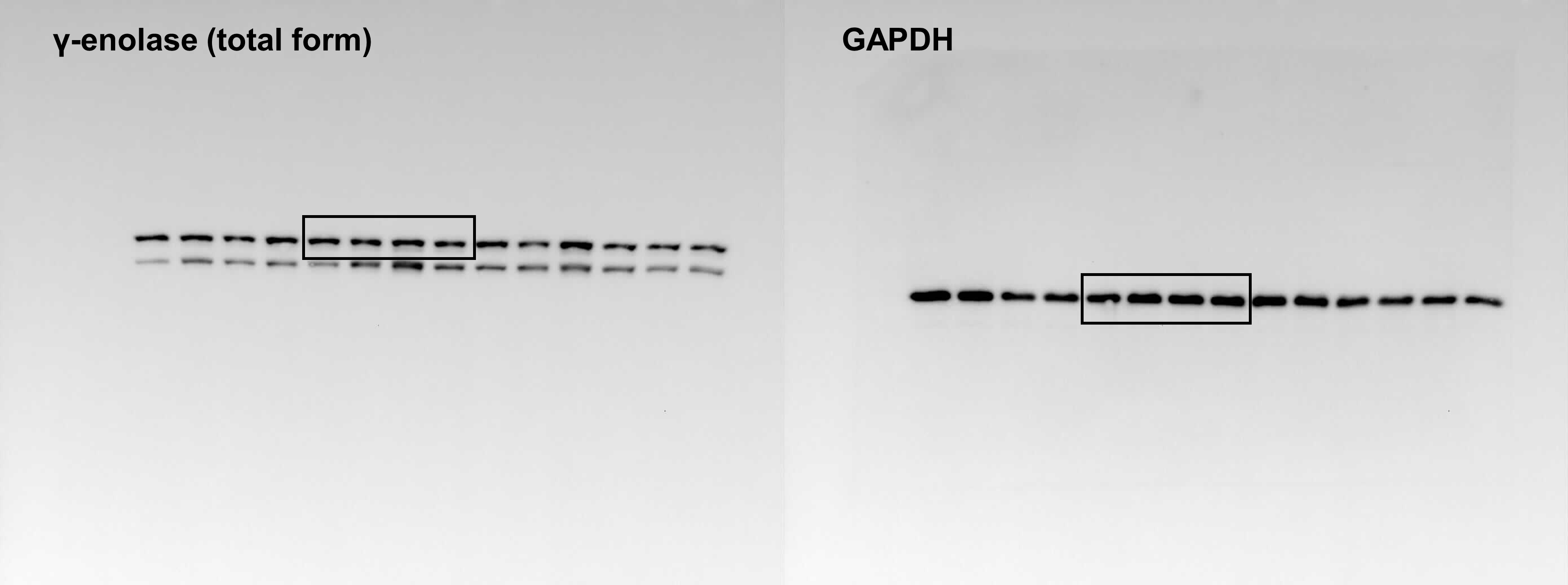
**

**
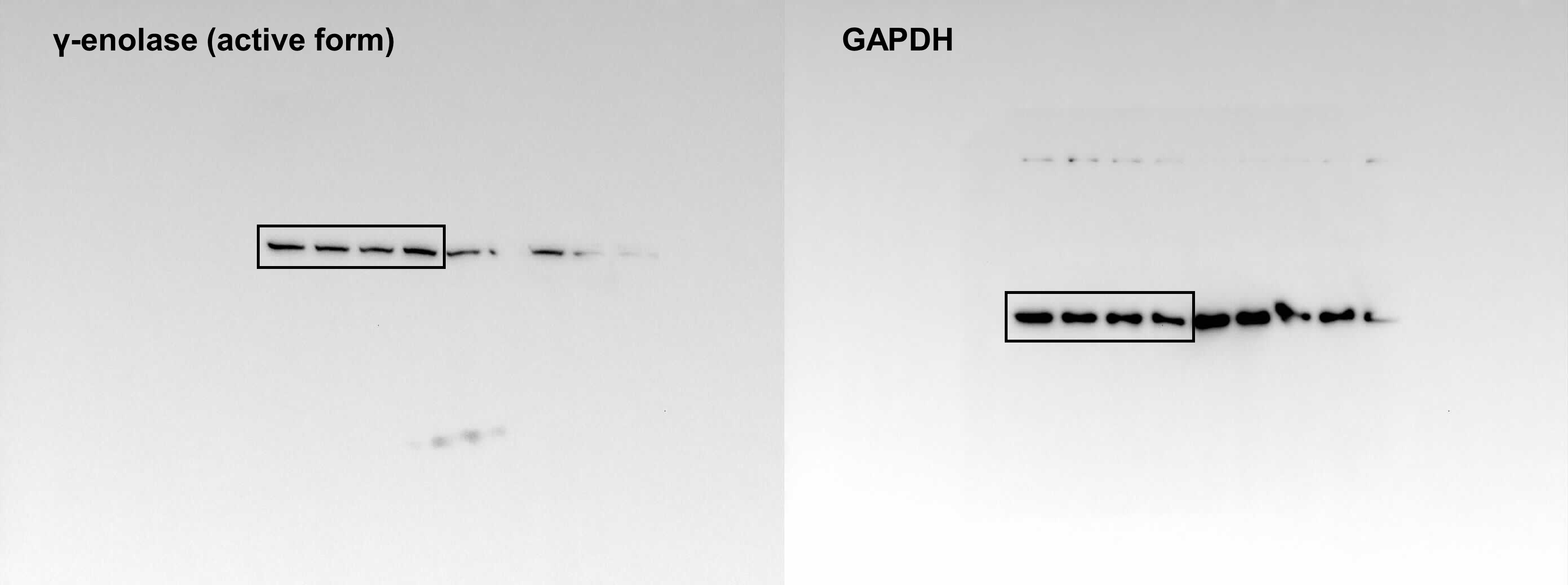
**

**Addition to Fig. 2C:** Raw images of the representative western-blotted membranes of the expression of α-enolase and γ-enolase (total and active form) with the appropriate representative western-blotted membranes of the expression of GAPDH.

**
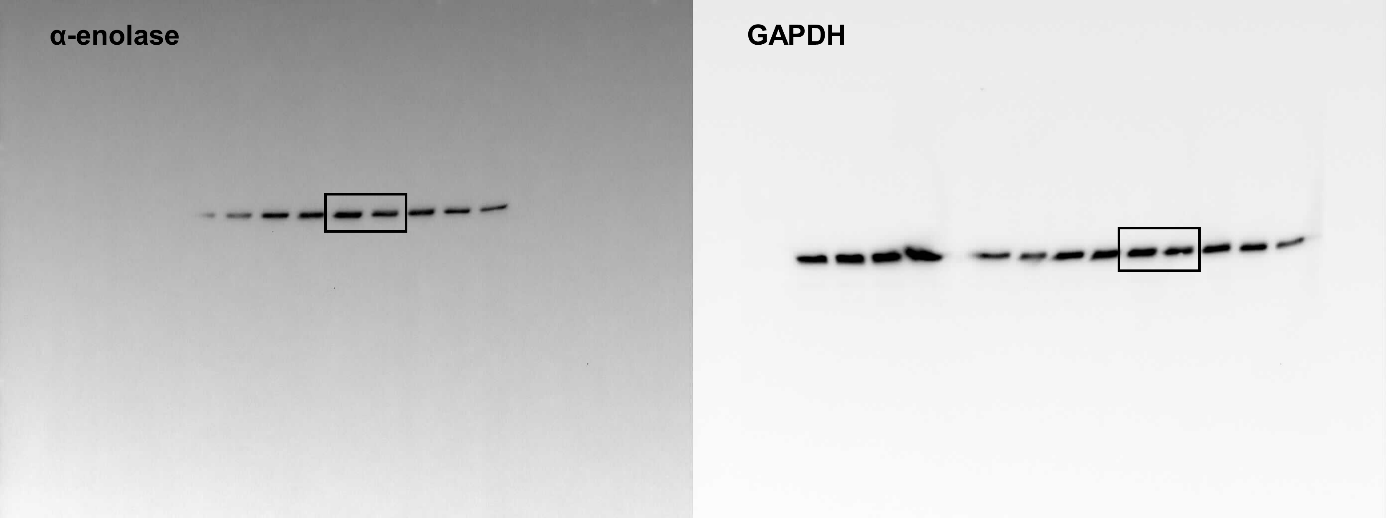
**

**
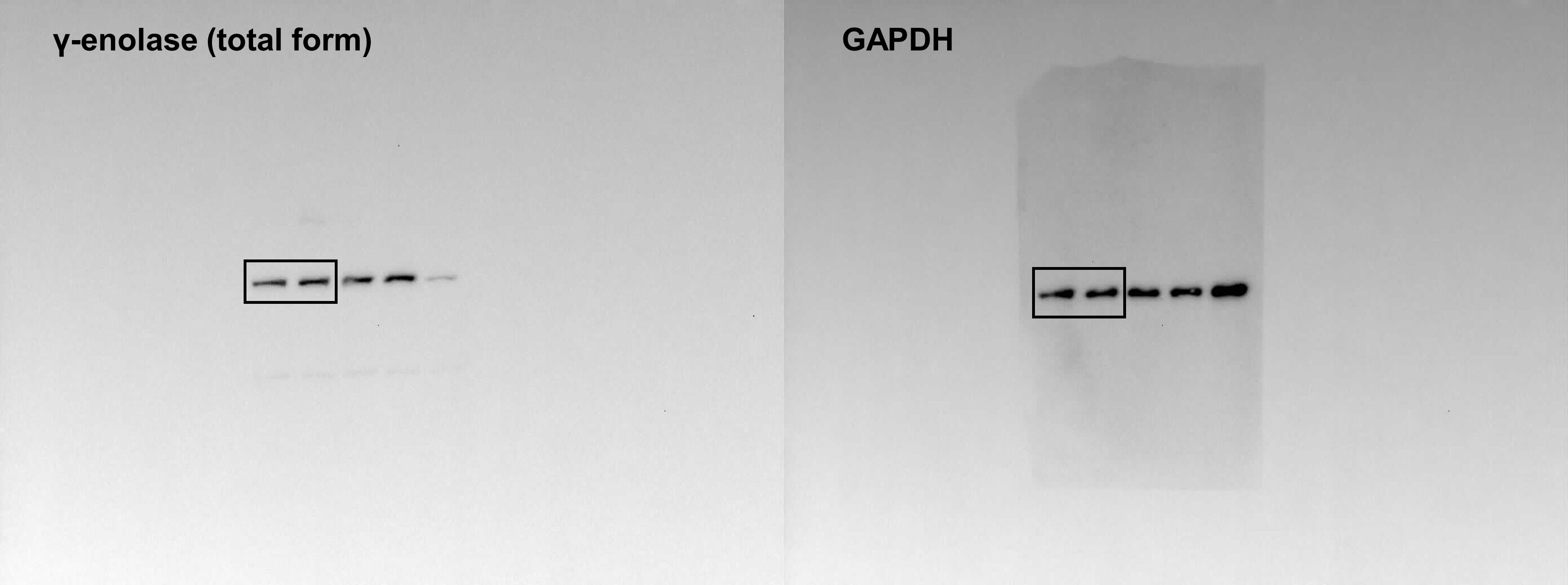
**

**
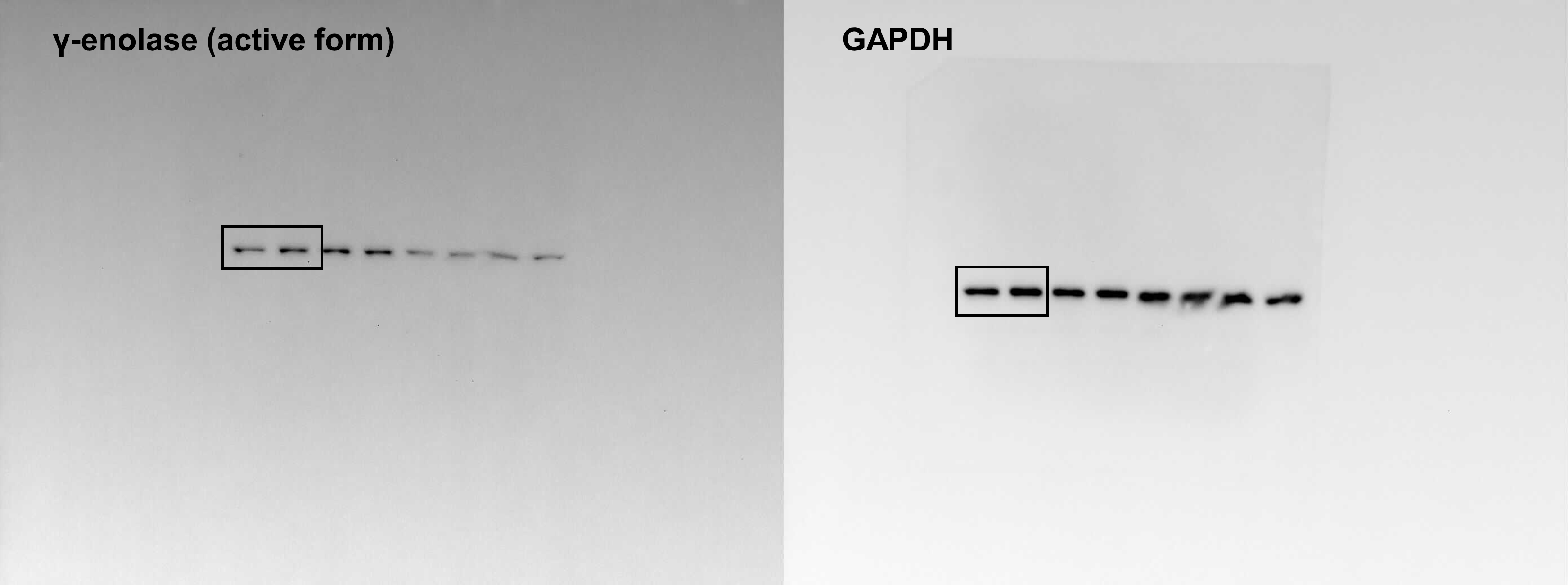
**

**Addition to Fig. 2D:** Raw images of the representative western-blotted membranes of the expression of α-enolase and γ-enolase (total and active form) with the appropriate representative western-blotted membranes of the expression of GAPDH**.**

**
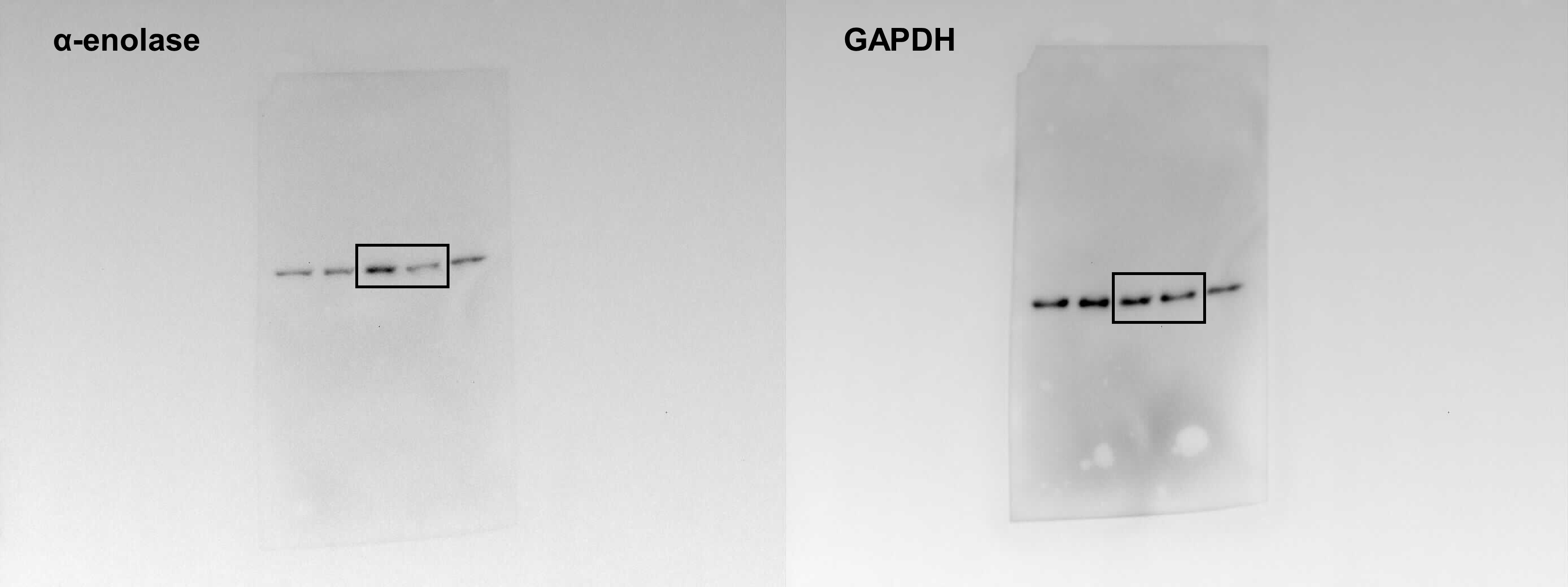
**

**
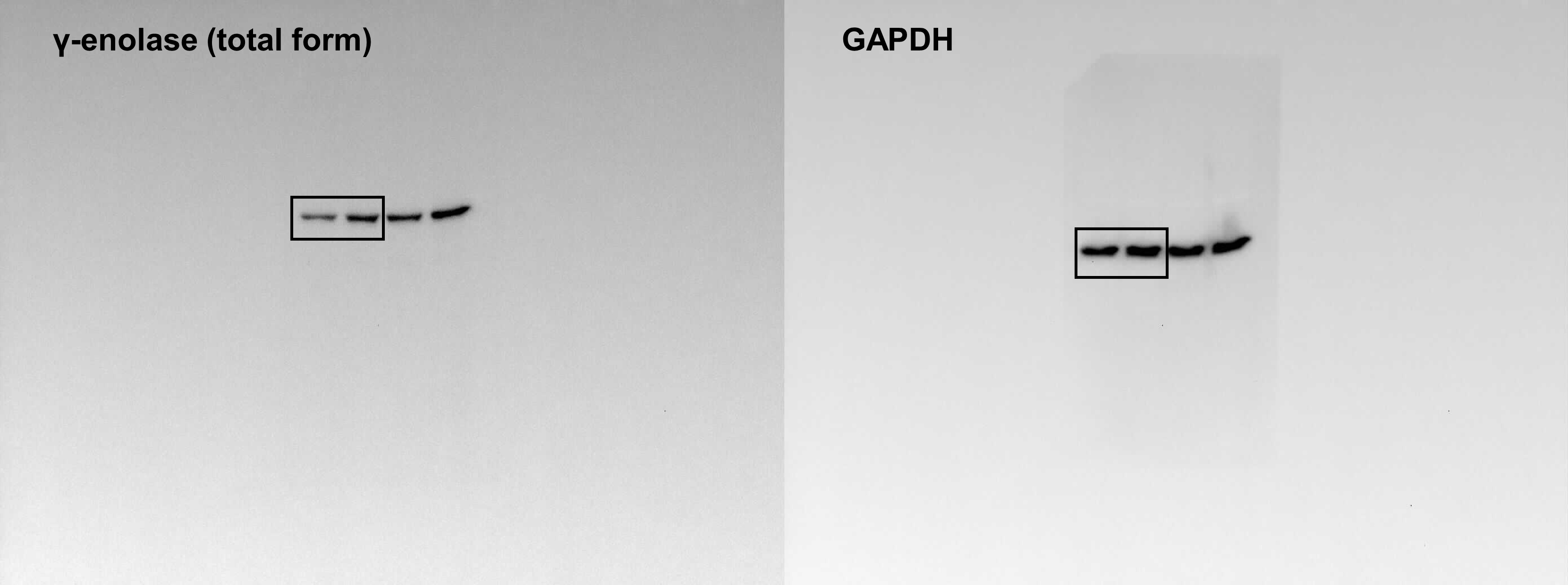
**

**
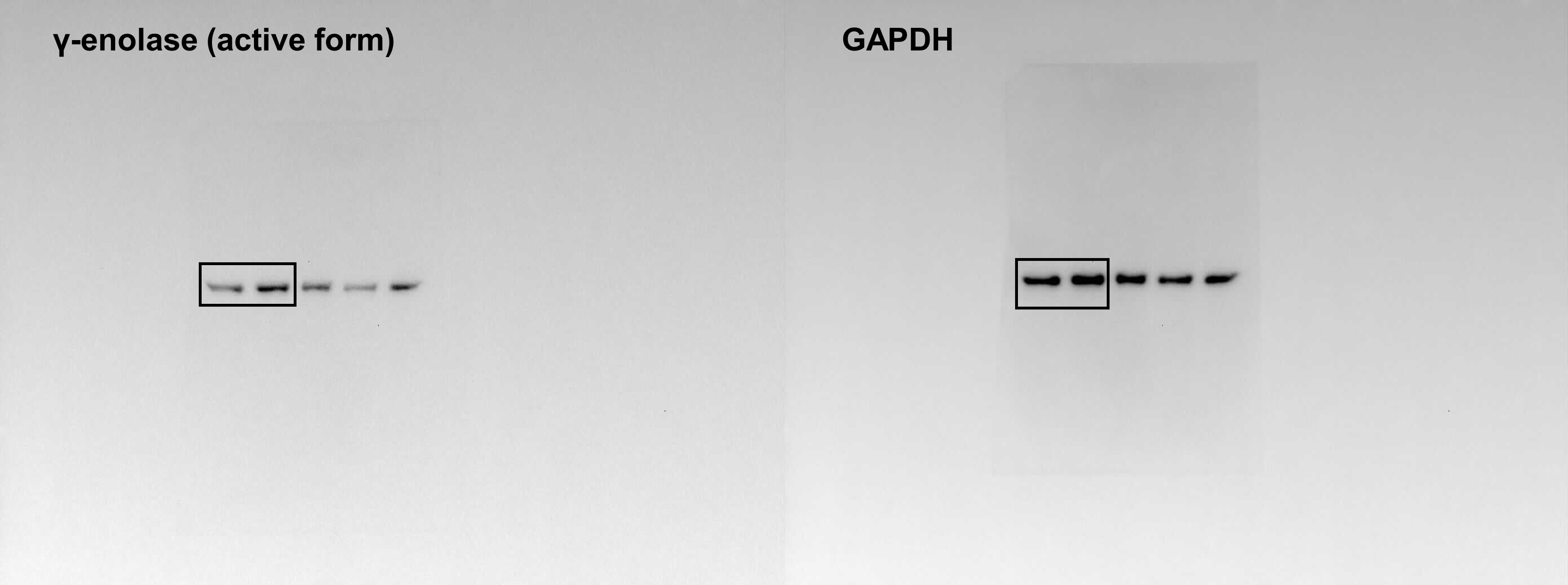
**

**Addition to Fig. 3A:** Raw images of the representative western-blotted membranes of the expression of α-enolase and γ-enolase (total and active form) with the appropriate representative western-blotted membranes of the expression of GAPDH.

**
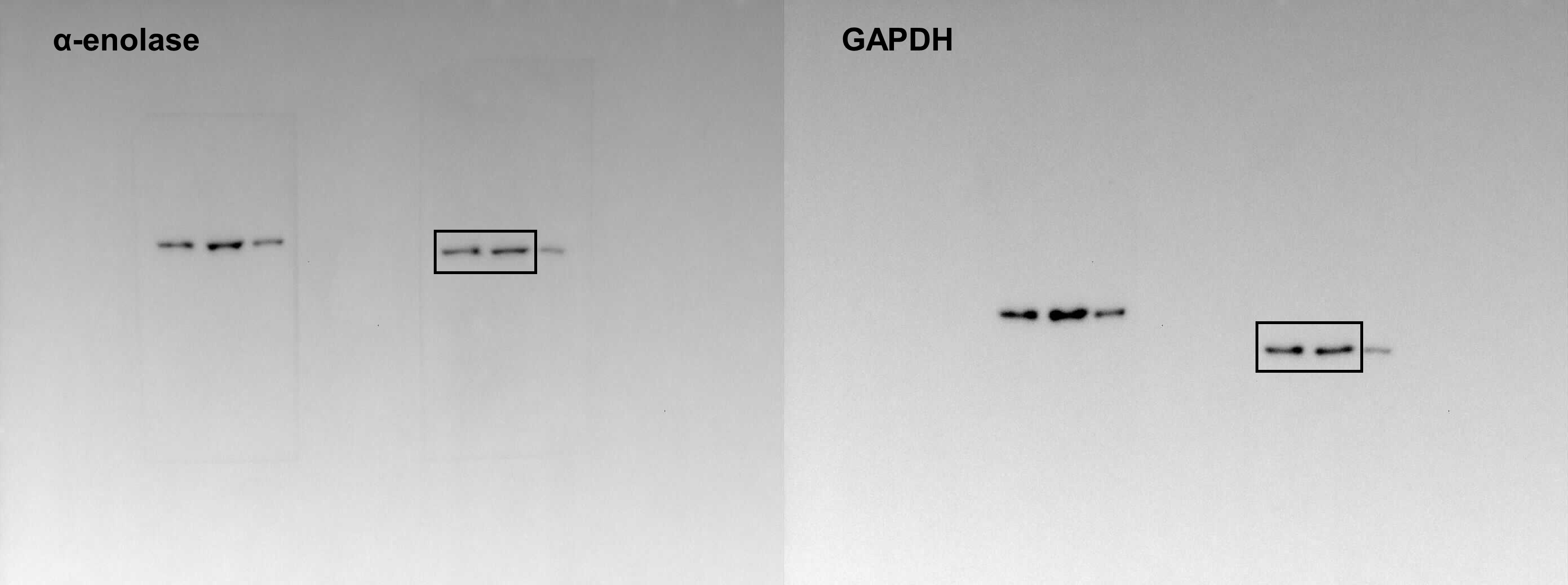
**

**
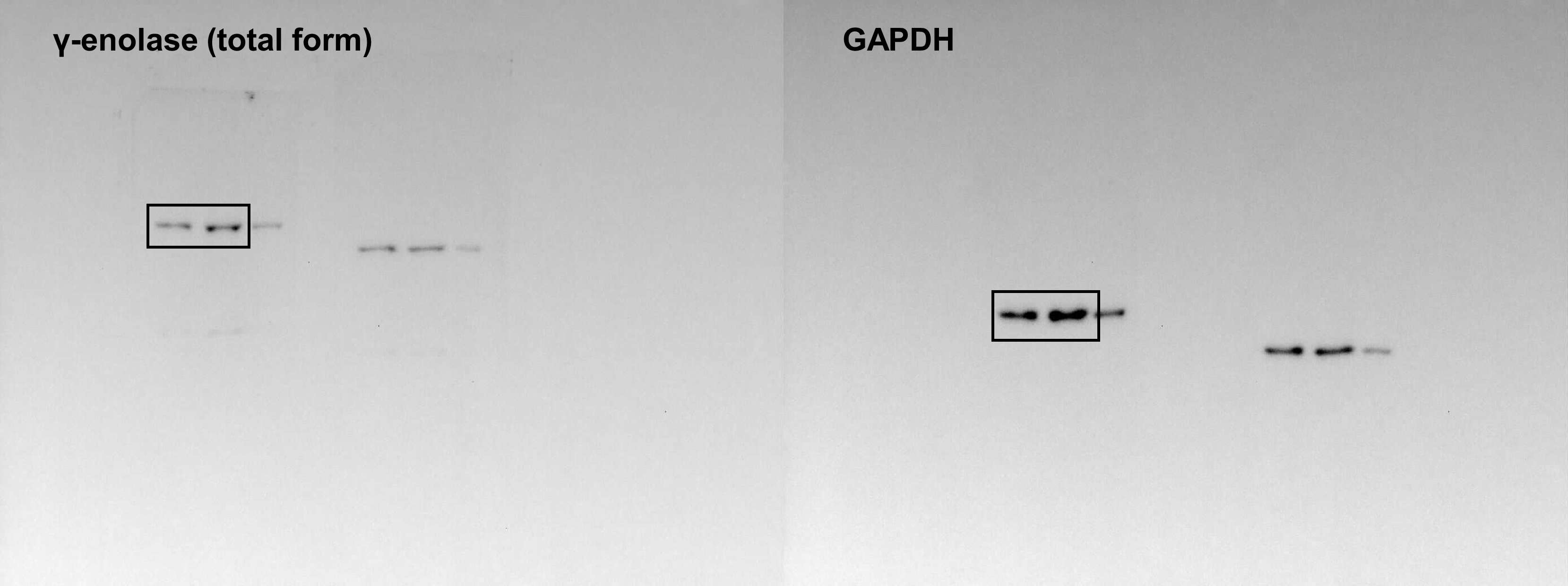
**

**
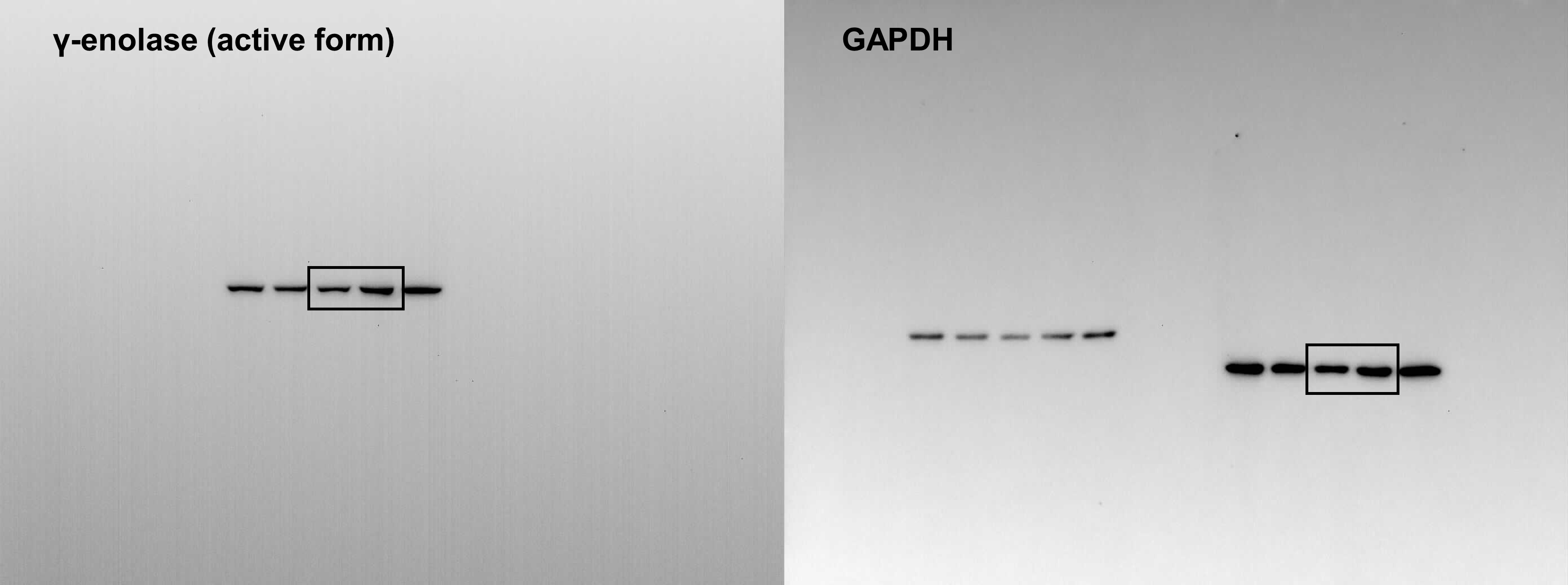
**

**Addition to Fig. 3B:** Raw images of the representative western-blotted membranes of the expression of α-enolase and γ-enolase (total and active form) with the appropriate representative western-blotted membranes of the expression of GAPDH.

**
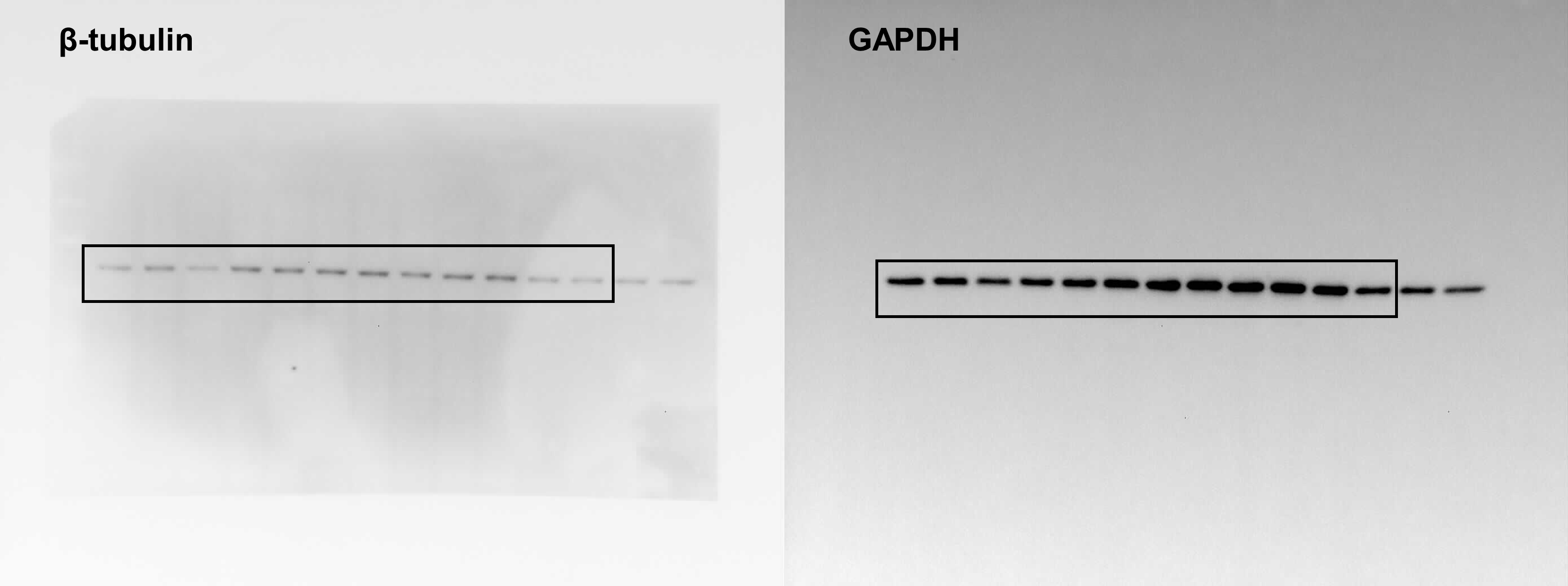
**

**Addition to Fig. 4C:** Raw images of the representative western-blotted membrane of the expression of β-tubulin with the appropriate representative western-blotted membrane of the expression of GAPDH.

**
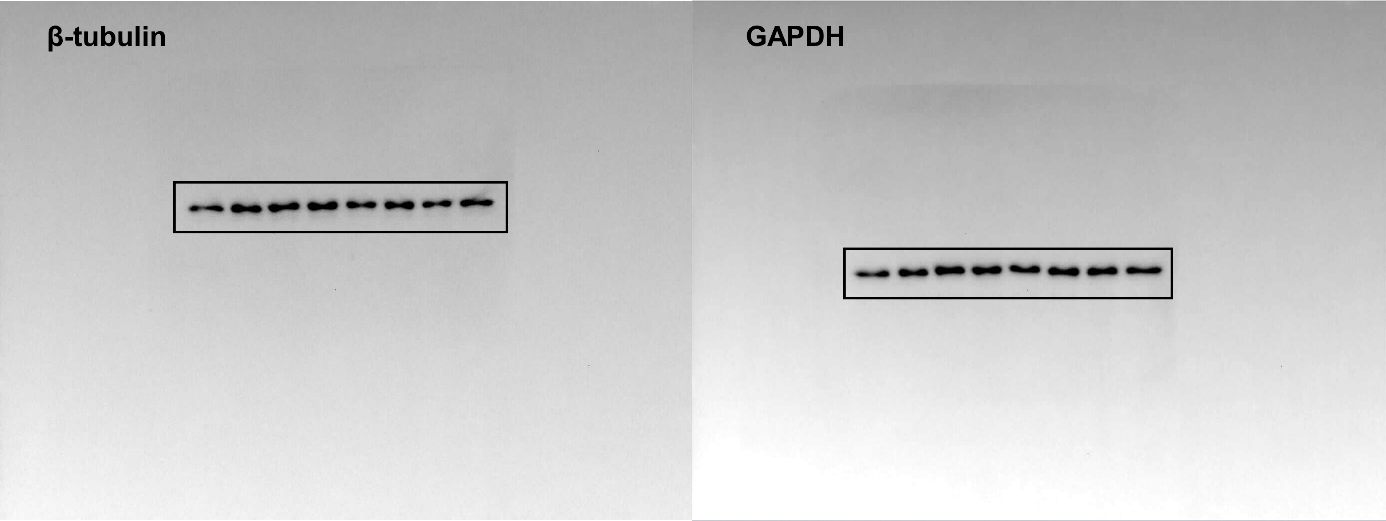
**

**Addition to Fig. 6D:** Raw images of the representative western-blotted membrane of the expression of β-tubulin with the appropriate representative western-blotted membrane of the expression of GAPDH.

**
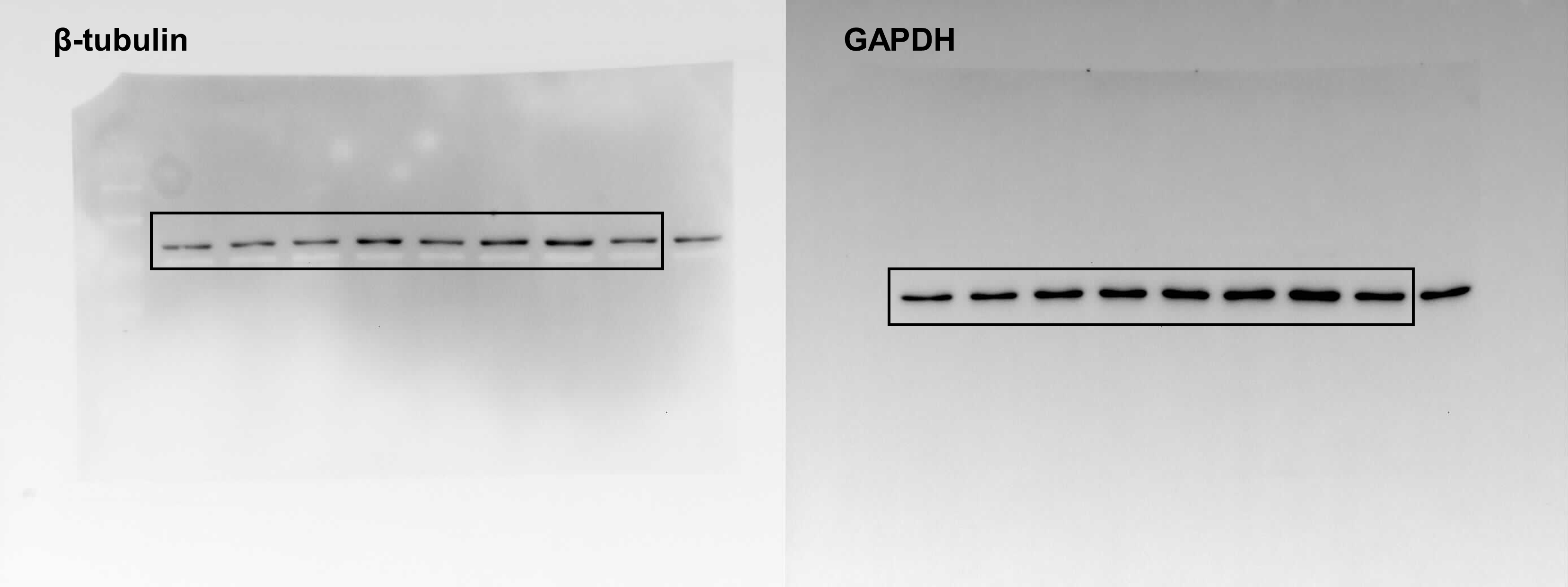
**

**Addition to Fig. 8D:** Raw images of the representative western-blotted membrane of the expression of β-tubulin with the appropriate representative western-blotted membrane of the expression of GAPDH.

**
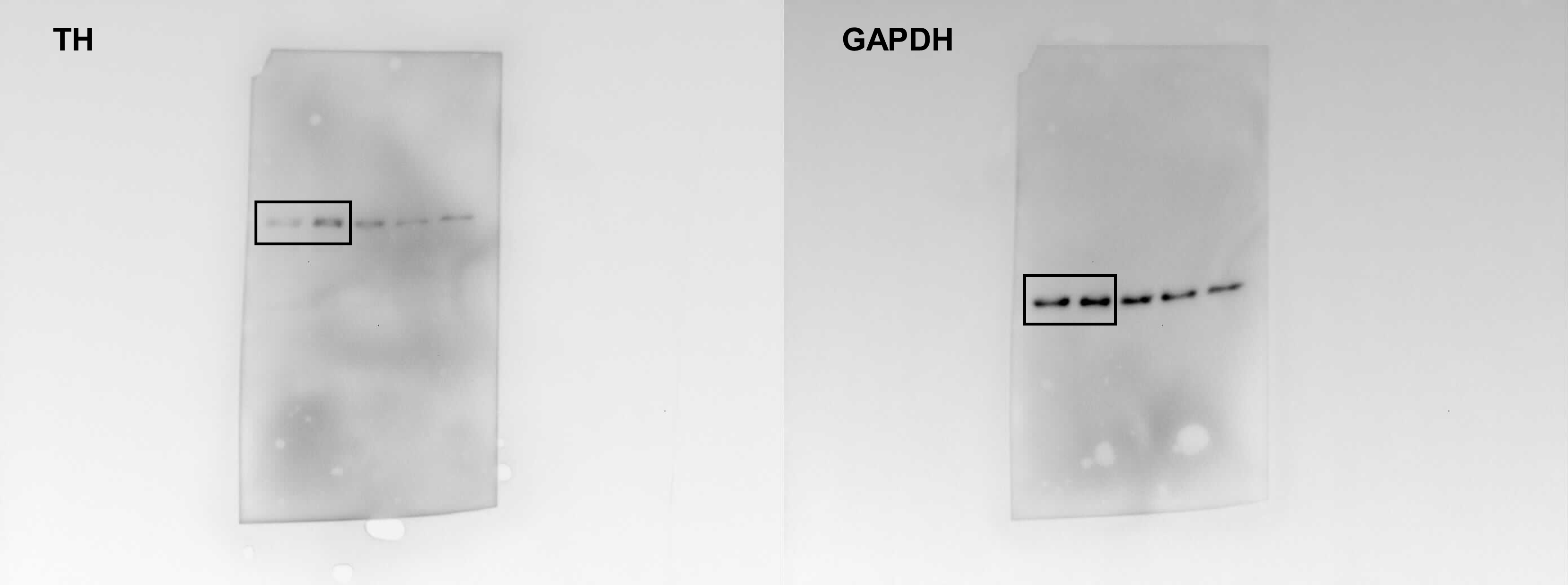
**

**Addition to Supplementary Fig. S4G:** Raw images of the representative western-blotted membrane of the expression of tyrosine (TH) with the appropriate representative western-blotted membrane of the expression of GAPDH.

**
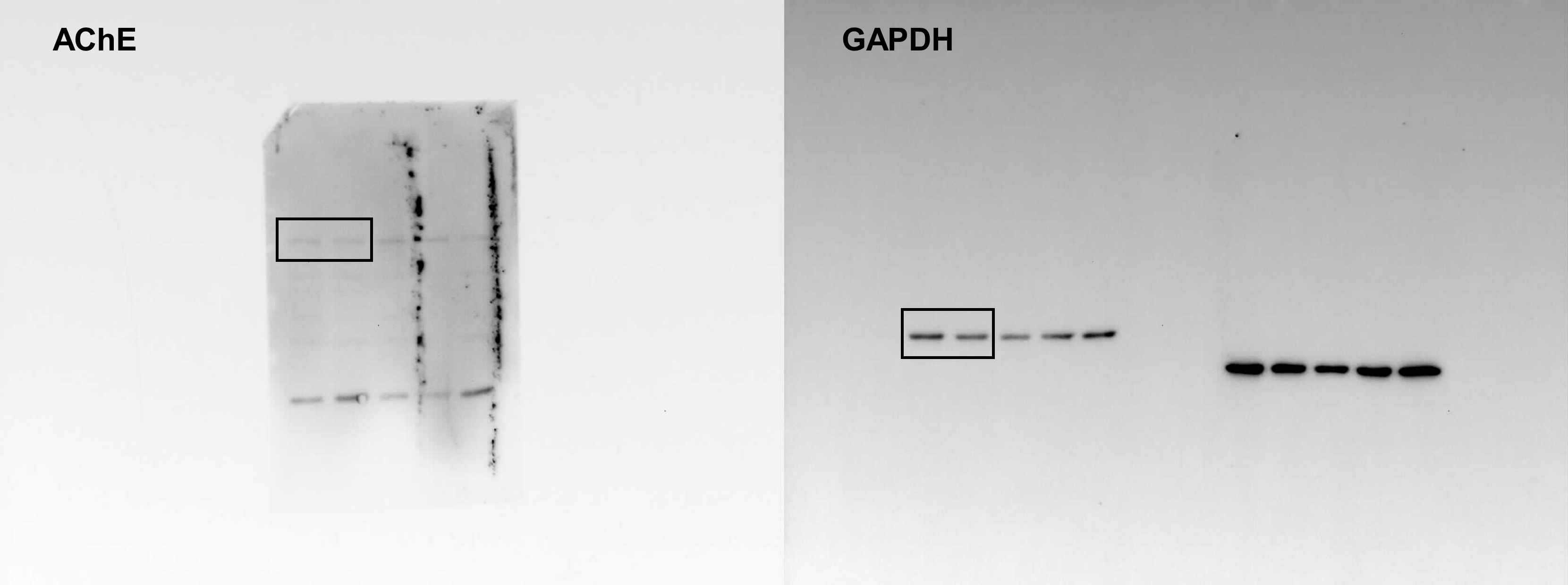
**

**Addition to Supplementary Fig. S4H:** Raw images of the representative western-blotted membrane of the expression of acetylcholinesterase (AChE) with the appropriate representative western-blotted membrane of the expression of GAPDH.

**
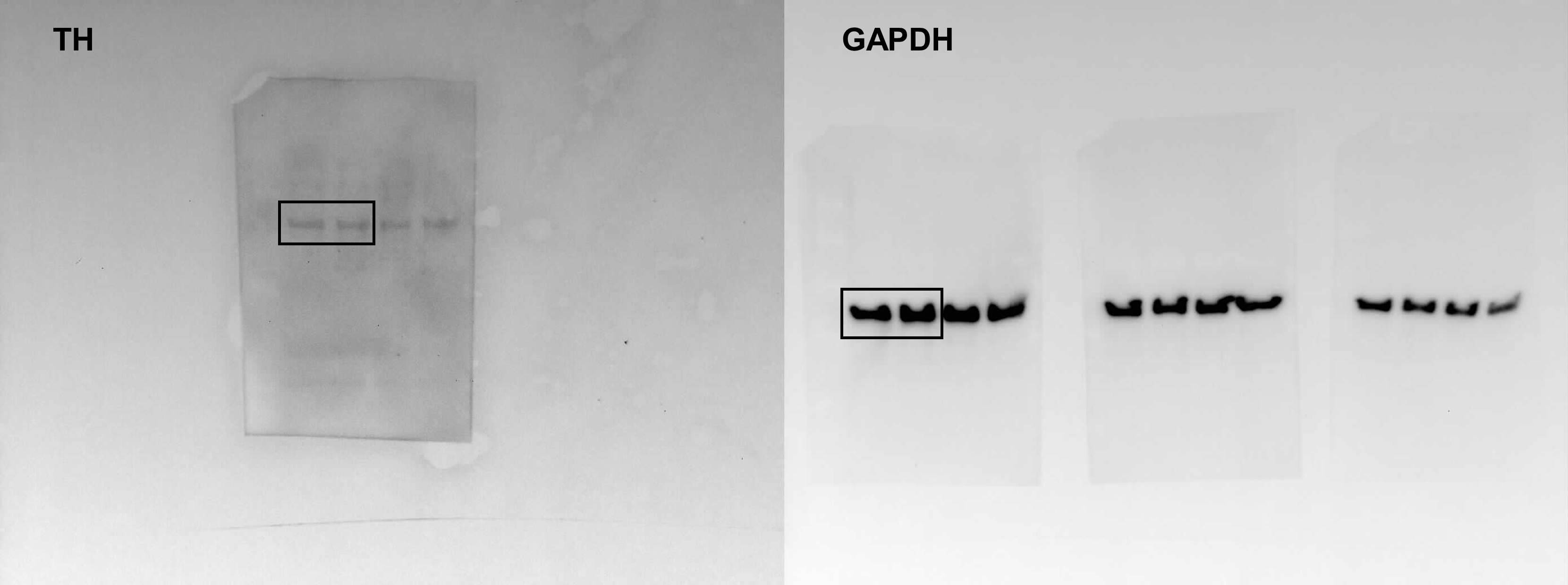
**

**Addition to Supplementary Fig. S4I:** Raw images of the representative western-blotted membrane of the expression of tyrosine (TH) with the appropriate representative western-blotted membrane of the expression of GAPDH.


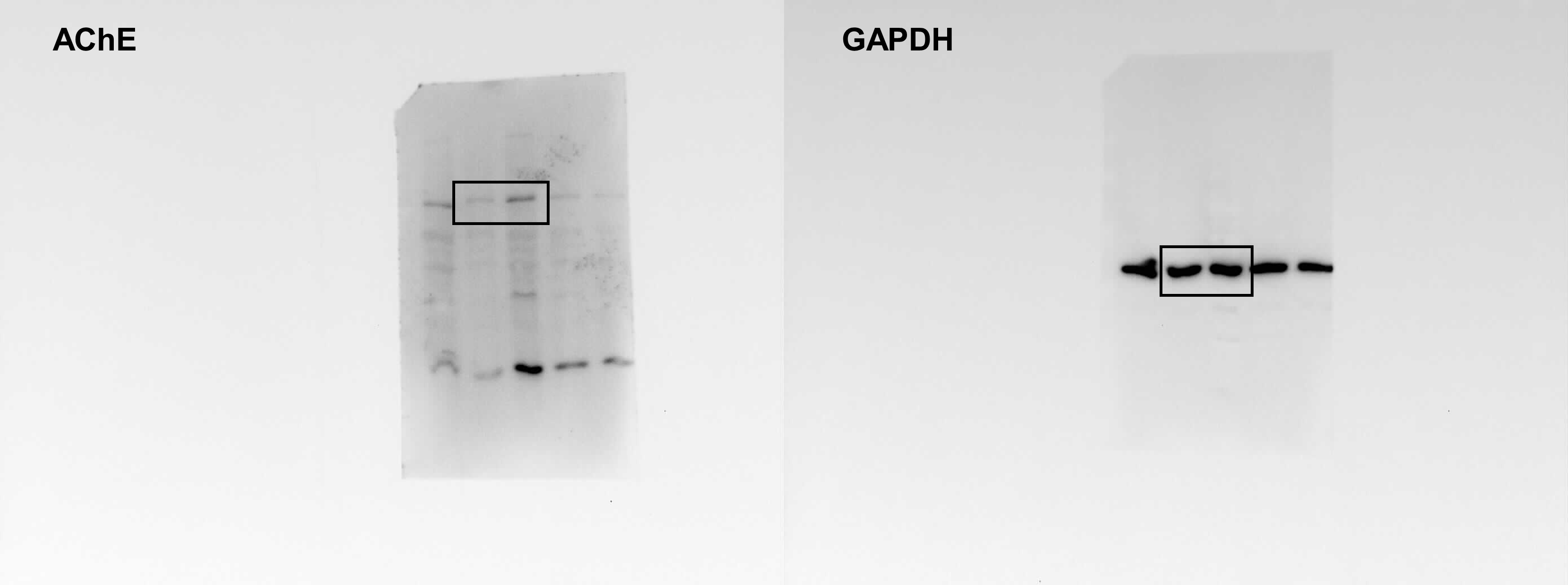


**Addition to Supplementary Fig. S4J:** Raw images of the representative western-blotted membrane of the expression of acetylcholinesterase (AChE) with the appropriate representative western-blotted membrane of the expression of GAPDH.


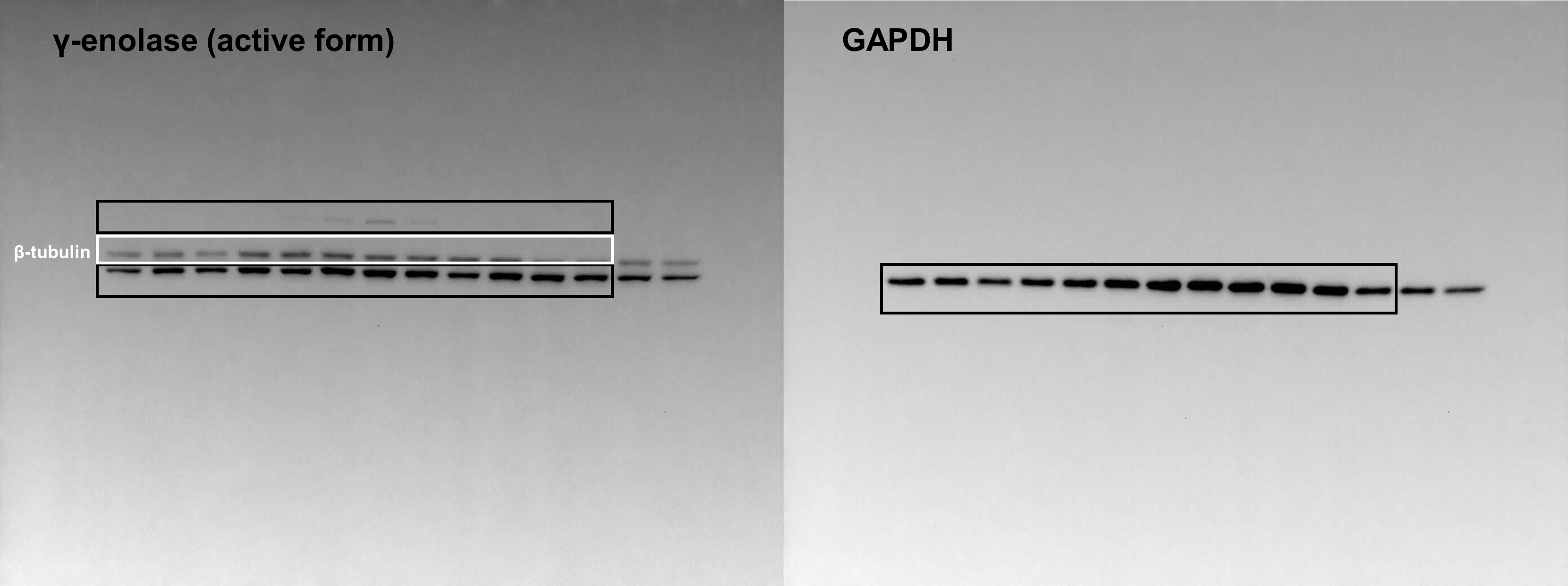


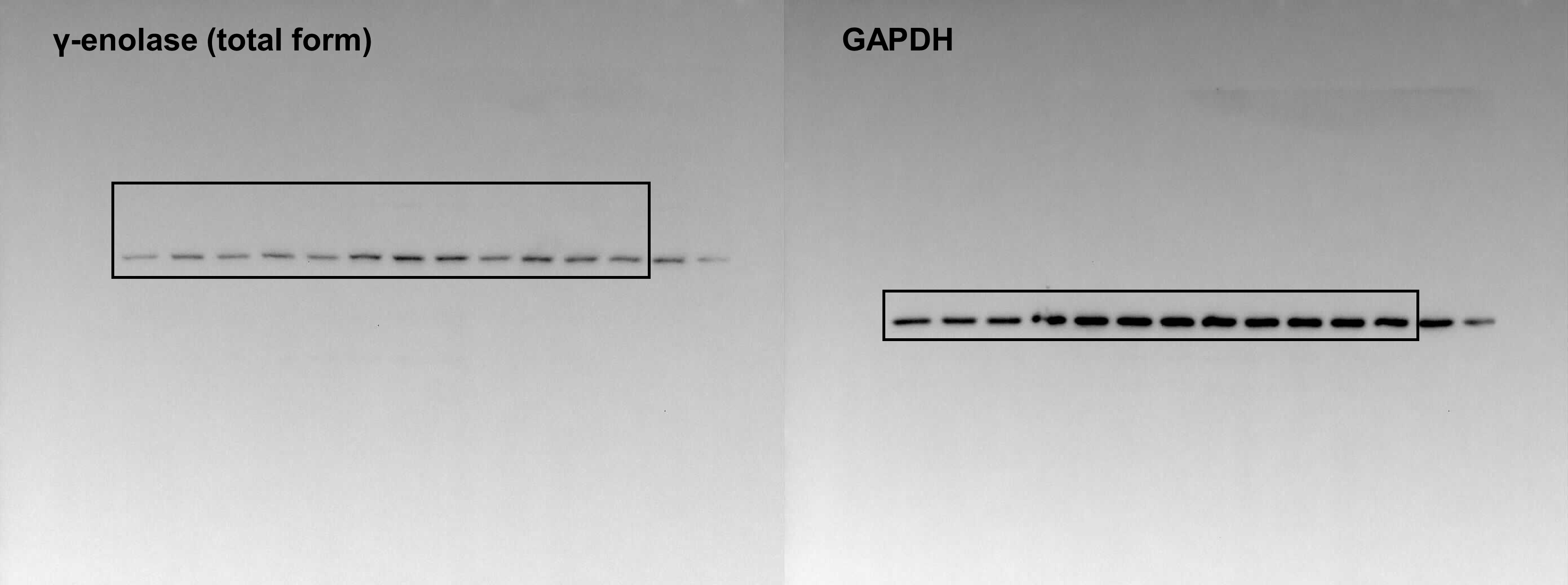


**Addition to Supplementary Fig. S5A:** Raw images of the representative western-blotted membranes of the expression of γ-enolase (active and total form) with the appropriate representative western-blotted membranes of the expression of GAPDH.


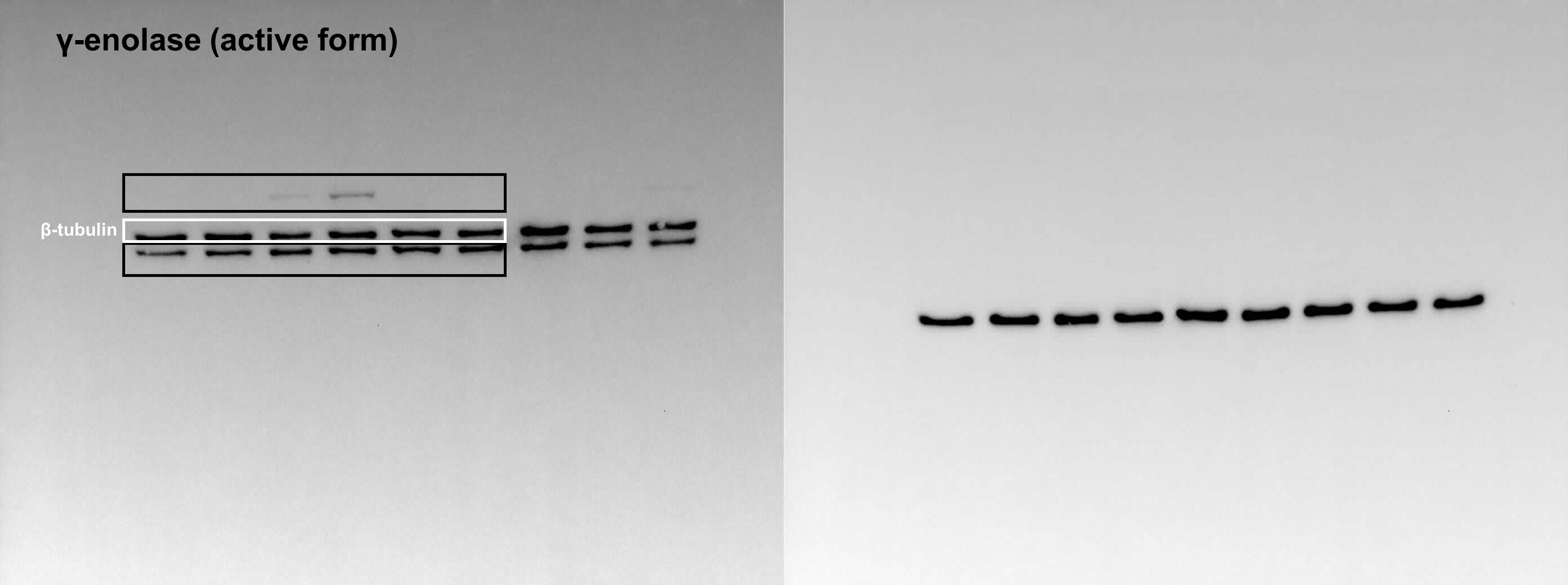


**
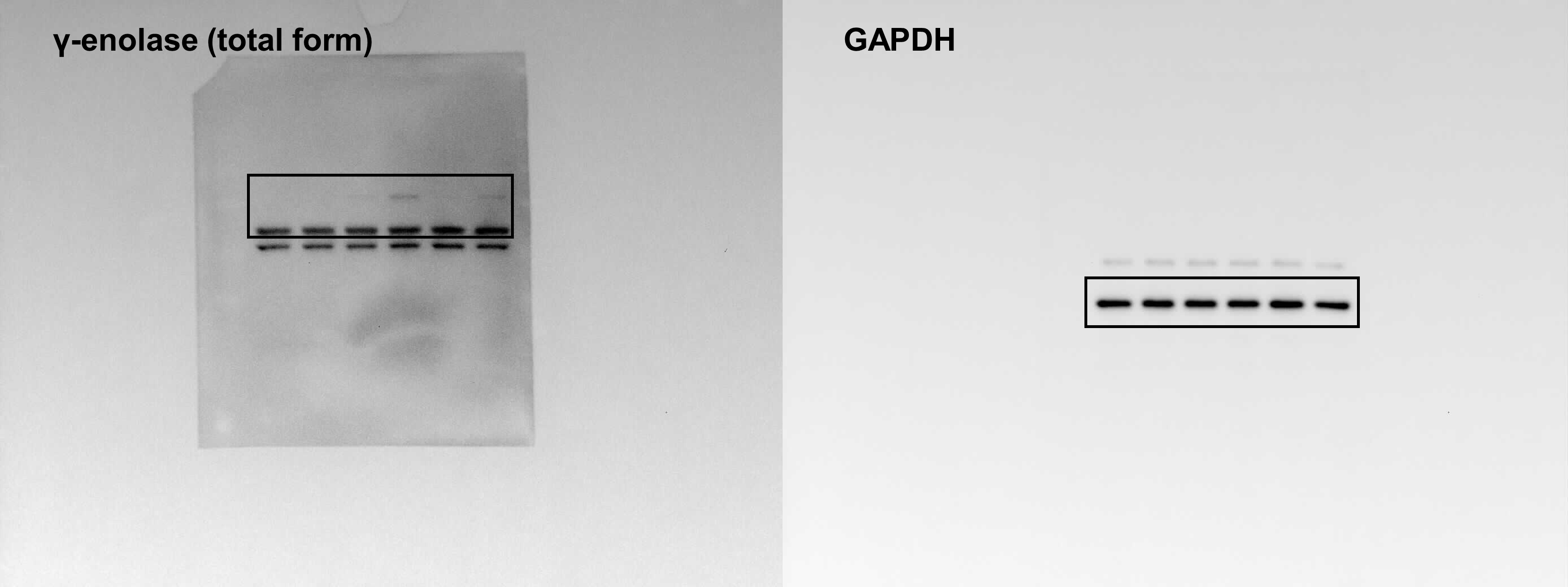
**

**Addition to Supplementary Fig. S5B:** Raw images of the representative western-blotted membranes of the expression of γ-enolase (active and total form) with the appropriate representative western-blotted membranes of the expression of GAPDH.

**
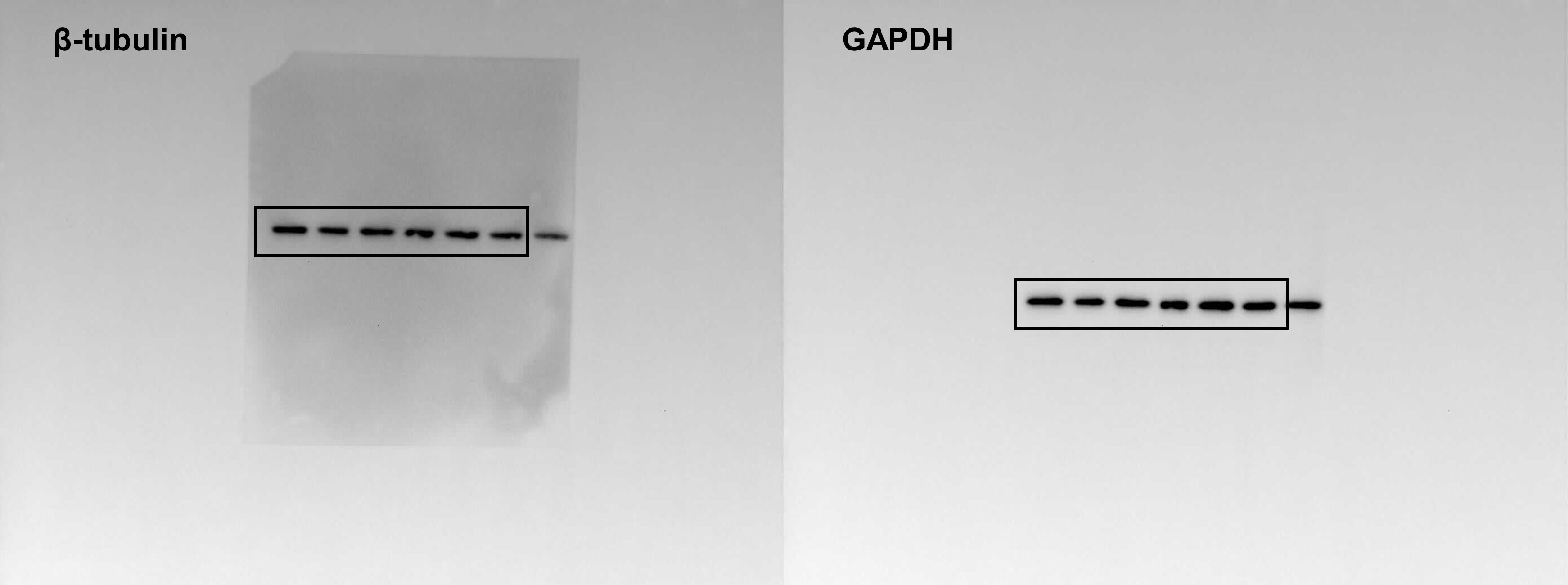
**

**Addition to Supplementary Fig. S6C:** Raw images of the representative western-blotted membrane of the expression of β-tubulin with the appropriate representative western-blotted membrane of the expression of GAPDH.

**
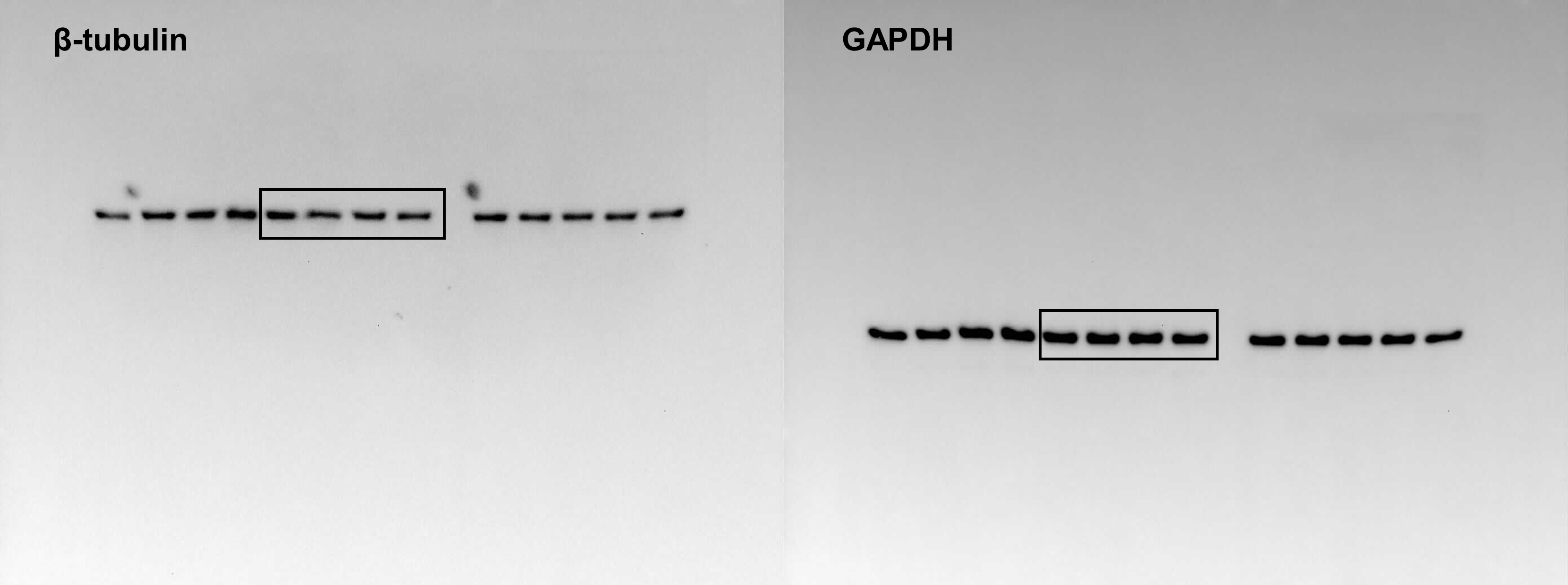
**

**Addition to Supplementary Fig. S7D:** Raw images of the representative western-blotted membrane of the expression of β-tubulin with the appropriate representative western-blotted membrane of the expression of GAPDH.

**
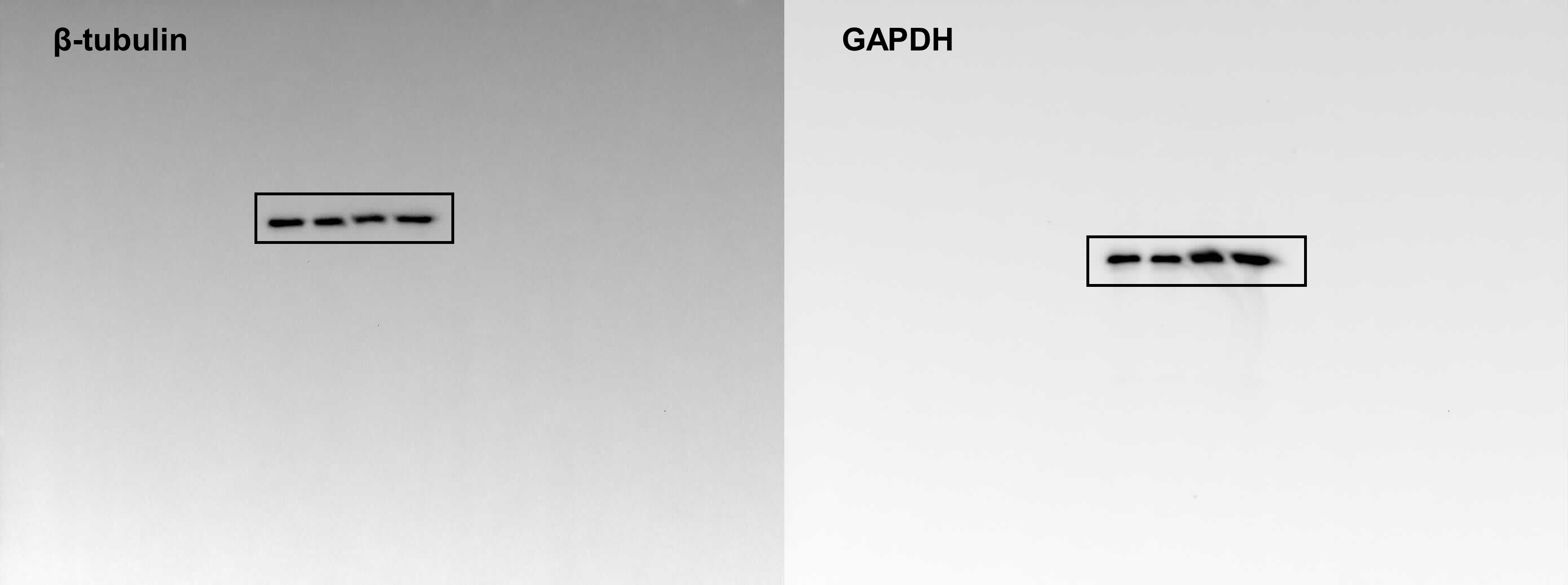
**

**Addition to Supplementary Fig. S10D:** Raw images of the representative western-blotted membrane of the expression of β-tubulin with the appropriate representative western-blotted membrane of the expression of GAPDH.

**
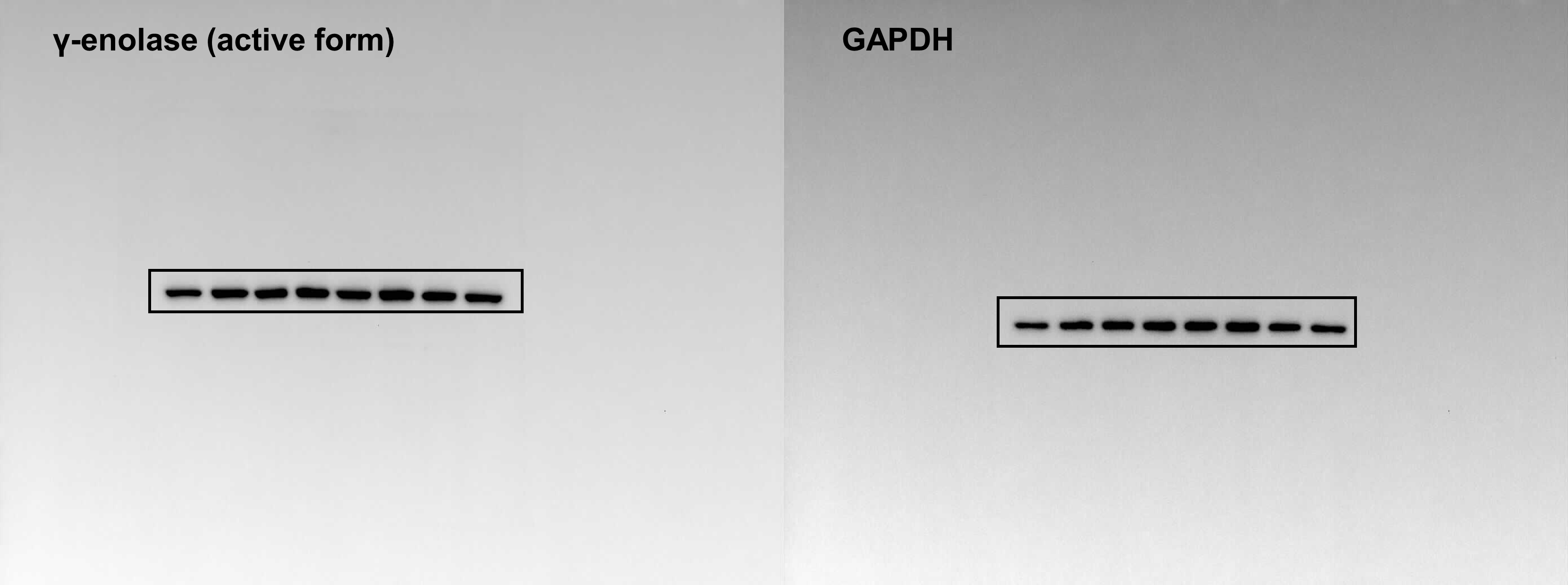
**

**
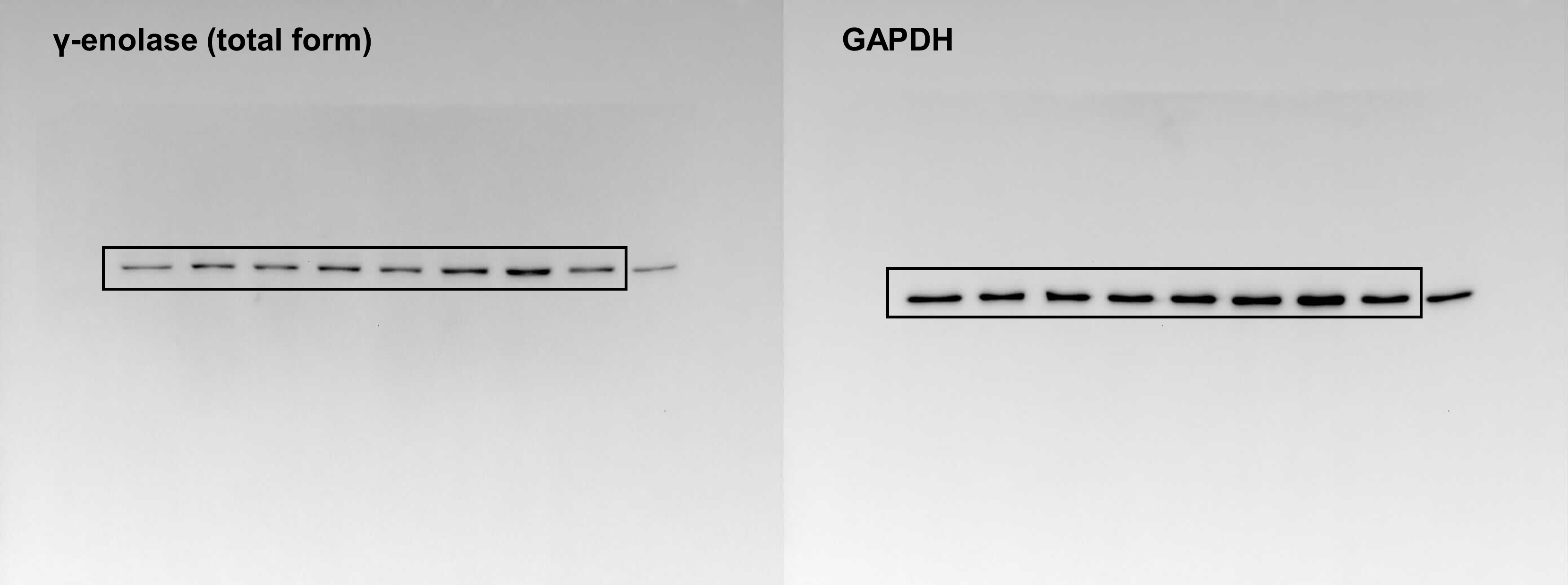
**

**Addition to Supplementary Fig. S11A:** Raw images of the representative western-blotted membranes of the expression of γ-enolase (active and total form) with the appropriate representative western-blotted membranes of the expression of GAPDH.

**
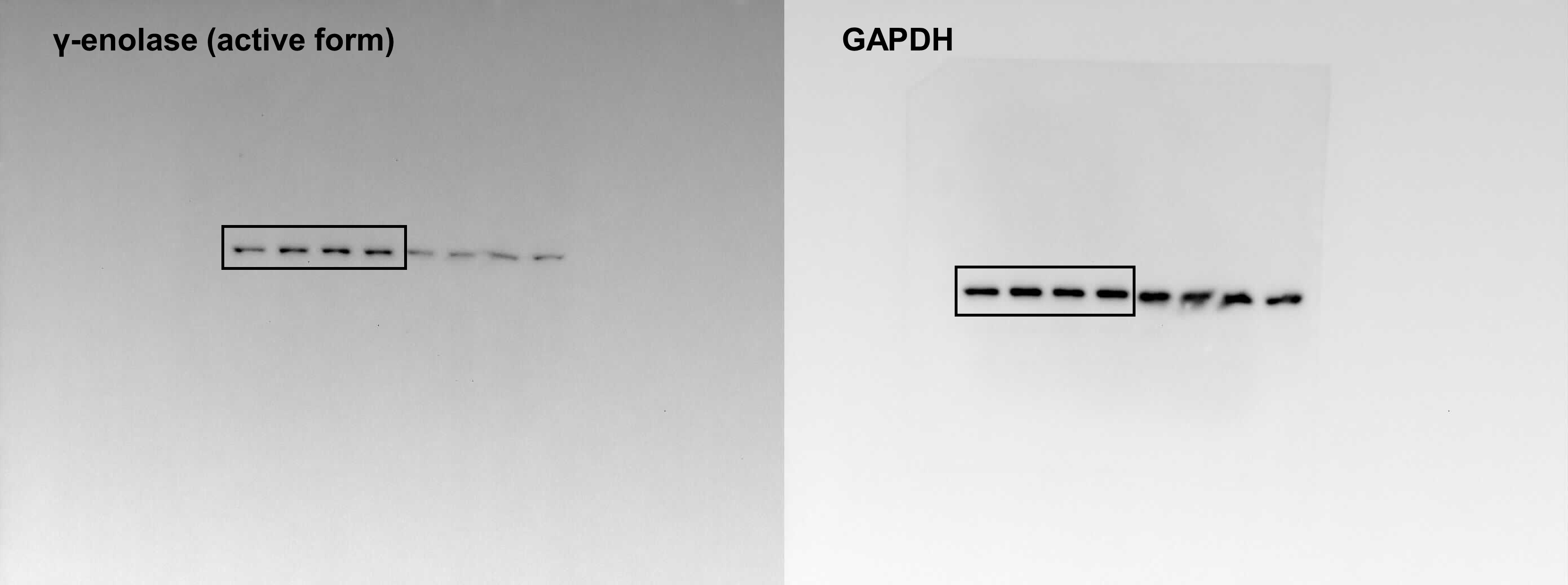
**


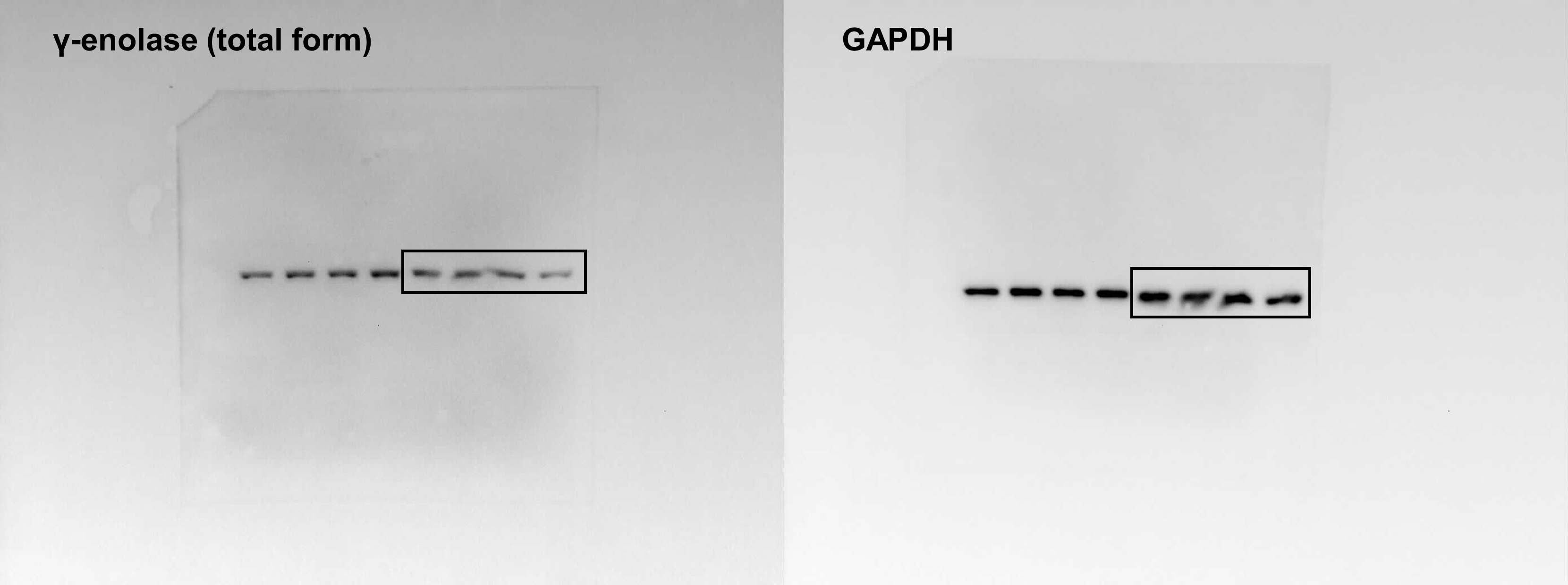


**Addition to Supplementary Fig. S11B**: Raw images of the representative western-blotted membranes of the expression of γ-enolase (active and total form) with the appropriate representative western-blotted membranes of the expression of GAPDH.
