# Supplementary material for: Neuronal Subtype-Specific Expression of γ-Enolase: Its Role in Neuronal Differentiation
Source: Neuromolecular Med. 2026 Jan 30;28(1):4. doi: 10.1007/s12017-025-08902-9 (PMC12858592; doi:10.1007/s12017-025-08902-9)
Supplement: Supplementary file 2 — Supplementary Material 2 [file 12017_2025_8902_MOESM2_ESM.docx]

**Neuronal Subtype-Specific Expression of γ-Enolase: Its Role in Neuronal Differentiation**

Selena Horvat ([selena.horvat@ffa.uni-lj.si](mailto:selena.horvat@ffa.uni-lj.si))^a^, Urša Pečar Fonović ([ursa.pecarfonovic@ffa.uni-lj.si](mailto:ursa.pecarfonovic@ffa.uni-lj.si))^a^, Nace Zidar ([nace.zidar@ffa.uni-lj.si](mailto:nace.zidar@ffa.uni-lj.si))^b^, Bojan Doljak ([bojan.doljak@ffa.uni-lj.si](mailto:bojan.doljak@ffa.uni-lj.si))^a^, Janko Kos ([janko.kos@ffa.uni-lj.si](mailto:janko.kos@ffa.uni-lj.si))^a,c^, Anja Pišlar ([anja.pislar@ffa.uni-lj.si](mailto:anja.pislar@ffa.uni-lj.si))^a,*^

^a^ Department of Pharmaceutical Biology, Faculty of Pharmacy, University of Ljubljana, Aškerčeva 7, 1000 Ljubljana, Slovenia

^b^ Department of Pharmaceutical Chemistry, Faculty of Pharmacy, University of Ljubljana, Aškerčeva 7, 1000 Ljubljana, Slovenia

^c^ Department of Biotechnology, Jožef Stefan Institute, Jamova 39, 1000 Ljubljana, Slovenia

***Correspondence: Anja Pišlar**

Department of Pharmaceutical Biology, Faculty of Pharmacy, University of Ljubljana, Aškerčeva 7, 1000 Ljubljana, Slovenia; Tel: +386-1-4769526; Fax: +386-1-4258031; E-mail: [anja.pislar@ffa.uni-lj.si](mailto:anja.pislar@ffa.uni-lj.si); ORCID iD: 0000-0002-1159-1024.

**Supplementary**

**
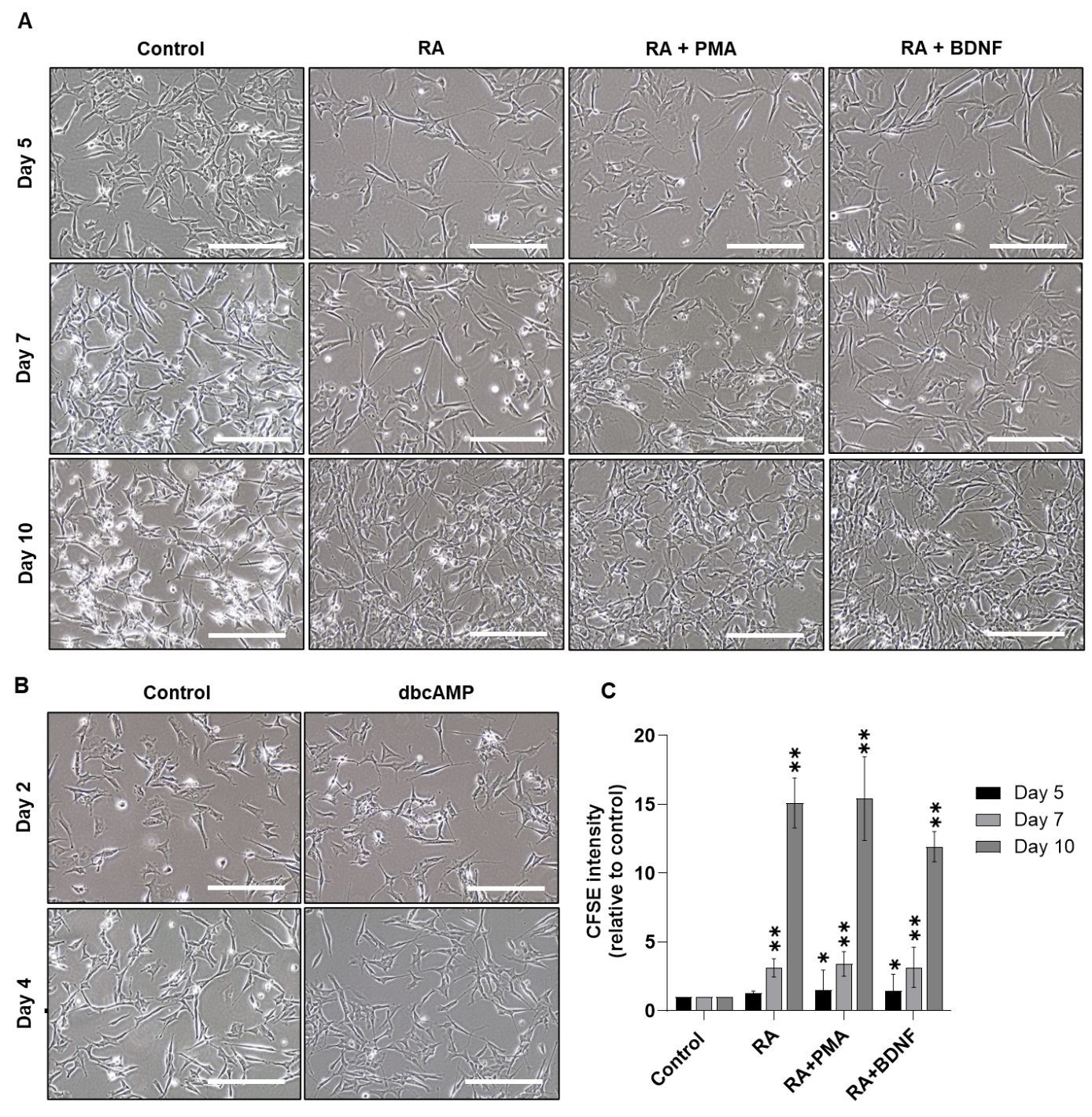
**

**Fig. S1. Time-course differentiation of SH-SY5Y cells into specific neuronal subtypes**. SH-SY5Y cells were seeded and maintained in complete growth media for 24 h. Afterwards, they were differentiated in reduced-serum media (RSM) supplemented with specific differentiation agents. For differentiation into dopaminergic- and cholinergic-like neuronal cells, RSM was supplemented with retinoic acid (RA, 10 μM). For differentiation into adrenergic-like neuronal cells, RSM was supplemented with N6,2′-O-dibutyryladenosine 3′,5′-cyclic monophosphate sodium salt (dbcAMP, 0.5 mM). On day 4, phorbol 12-myristate 13-acetate (PMA, 80 nM) or brain-derived neurotrophic factor (BDNF, 50 ng/mL) were added to the differentiation medium for dopaminergic- and cholinergic-like neuronal cells, respectively. Cholinergic- and dopaminergic-like differentiation was achieved by day 7, and adrenergic-like differentiation was achieved by day 4. (**A, B**) Phase-contrast images were captured at various time points: on days 5, 7, and 10 for cholinergic- and dopaminergic-like neuronal cells (**A**) and on days 2 and 4 for adrenergic-like neuronal cells (**B**). Scale bars: 100 μm. (**C**) The proliferation rates of dopaminergic-like and cholinergic-like neuronal cells were assessed with carboxyfluorescein succinimidyl ester (CFSE) labeling and flow cytometry on days 5, 7, and 10 of differentiation. Data are shown as mean ± 95 % CI from N = 2-3 independent experiments, each performed in duplicate. Statistical significance was assessed by one-way ANOVA followed by Tukey’s post hoc test. Exact p-values: day 5 - Control vs RA, p = 0.191; Control vs RA+PMA, p = 0.031; Control vs RA+BDNF, p = 0.039; day 7 - Control vs RA, p = 0.001; Control vs RA+PMA, p = 0.001; Control vs RA+BDNF, p = 0.001; day 10 - Control vs RA, p = 0.001; Control vs RA+PMA, p = 0.001; Control vs RA+BDNF, p = 0.001.

**
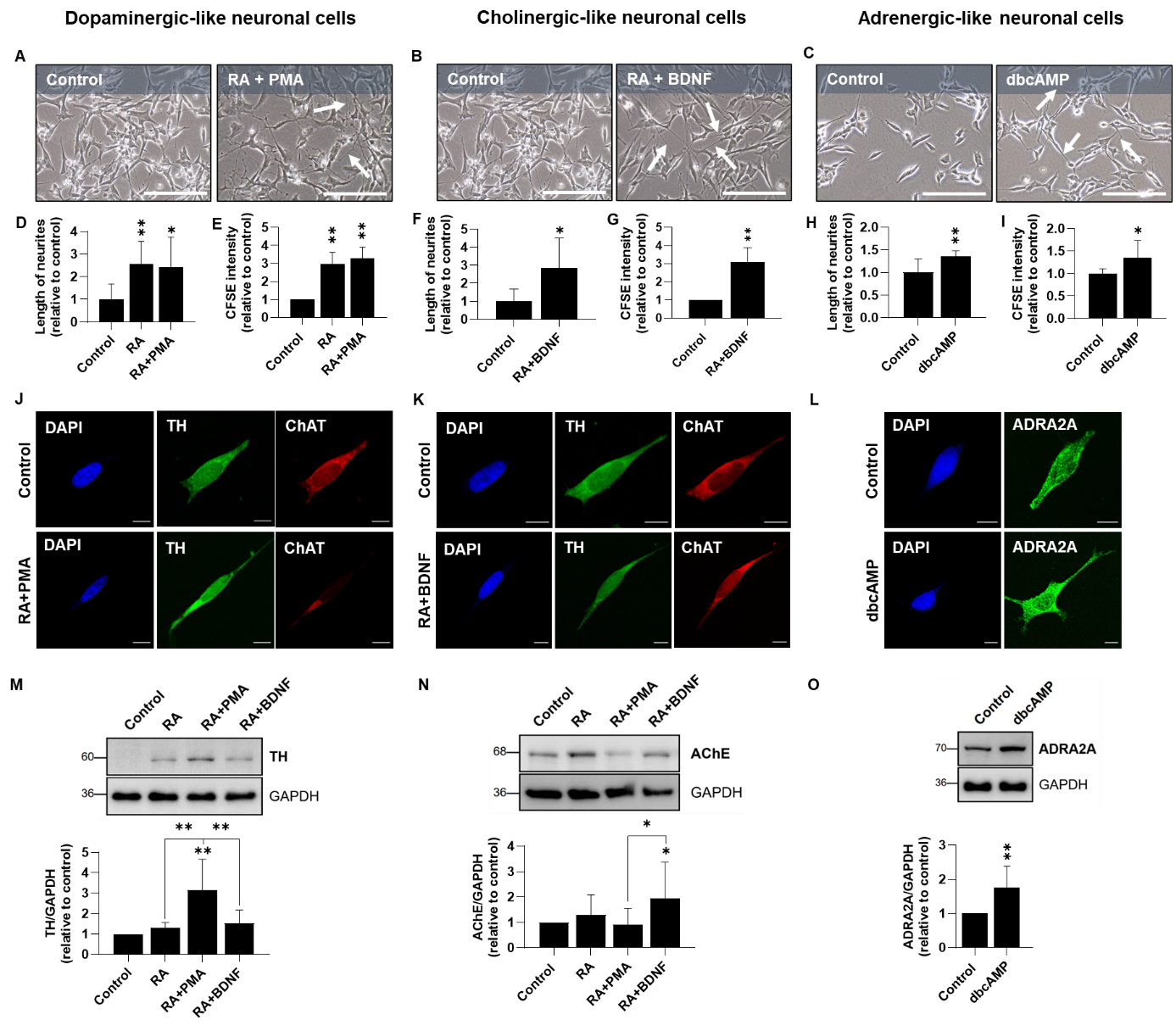
**

**Fig. S2. Differentiation of SH-SY5Y cells into specific neuronal subtypes. (A–C)** Representative phase-contrast images of SH-SY5Y cells differentiated into (**A**) dopaminergic-like neuronal cells, (**B**) cholinergic-like neuronal cells, and (**C**) adrenergic-like neuronal cells. White arrows indicate cell extensions. Scale bars: 100 μm. **(D, F, H)** Neurite lengths in (**D**) dopaminergic-, (**F**) cholinergic-, and (**H**) adrenergic-like neuronal cells were determined in pixels when the extensions were longer than the cell diameter using ImageJ software. Data are shown as mean ± 95 % CI from N = 3 independent experiments, each performed in duplicate. Statistical significance was assessed by one-way ANOVA followed by Tukey’s post hoc test. Exact p-values: (**D**) Control vs RA, p = 0.008; Control vs RA+PMA, p = 0.013; RA vs RA+PMA, p = 0.900; (**F**) Control vs RA+BDNF, p = 0.012; (**G**) Control vs dbcAMP, p = 0.009. (**E, G, I**) The proliferation rates of (**E**) dopaminergic-, (**G**) cholinergic-, and (**I**) adrenergic-like neuronal cells were assessed with carboxyfluorescein succinimidyl ester (CFSE) labeling and flow cytometry. Data are shown as mean ± 95 % CI from N = 3-4 independent experiments, each performed in duplicate. Statistical significance was assessed by one-way ANOVA followed by Tukey’s post hoc test. Exact p-values: (**E**) Control vs RA, p = 0.001; Control vs RA+PMA, p = 0.001; RA vs RA+PMA p = 0.770; (**G**) Control vs RA+BDNF, p = 0.001; (**I**) Control vs dbcAMP, p = 0.031. (**J, K, L**) Representative images of immunofluorescence staining for tyrosine hydroxylase (TH, green) and choline acetyltransferase (ChAT, red) in (**J**) dopaminergic- and (**K**) cholinergic-like neuronal cells and α-2 adrenergic receptor (ADRA2A, green) in (**L**) adrenergic-like neuronal cells. Nuclei were counterstained with DAPI (blue). Scale bars: 10 μm. Two independent experiments (N=2) were performed. (**M, N, O)** Representative western blots (top) and quantification (bottom) of the expression of TH in dopaminergic-like neuronal cells (**M**), acetylcholinesterase (AChE) in cholinergic-like neuronal cells (**N**), and ADRA2A in adrenergic-like neuronal cells (**O**). Protein levels are normalized to GAPDH. Data are shown as mean ± 95 % CI from N = 2-4 independent experiments. Statistical significance was assessed by one-way ANOVA followed by Tukey’s post hoc test. Exact p-values: (**M**) Control vs RA, p = 0.846; Control vs RA+PMA, p = 0.001; Control vs RA+BDNF, p = 0.458; RA vs RA+PMA, p = 0.004; RA vs RA+BDNF, p = 0.900; RA+PMA vs RA+BDNF, p = 0.006; (**N**) Control vs RA, p = 0.724; Control vs RA+PMA, p = 0.900; Control vs RA+BDNF, p = 0.044; RA vs RA+PMA, p = 0.567; RA vs RA+BDNF, p = 0.183; RA+PMA vs RA+BDNF, p = 0.029; (**O**) Control vs dbcAMP, p = 0.004. Data were obtained after 7 days of differentiation into dopaminergic- and cholinergic-like neuronal cells and after 4 days of differentiation into adrenergic-like neuronal cells.

**
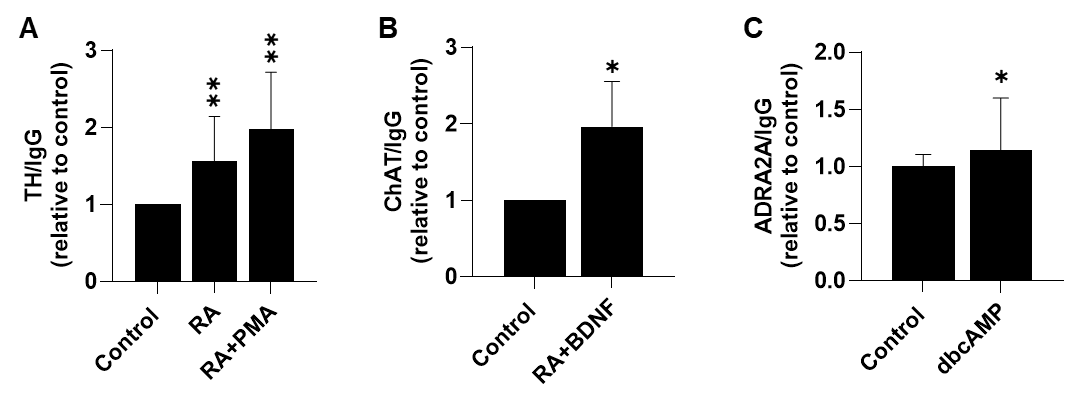
**

**Fig. S3. The expression of specific neuronal markers in SH-SY5Y cells differentiated into specific subtypes.** (**A-C**) Flow cytometry analysis of tyrosine hydroxylase (TH, **A**), choline acetyltransferase (ChAT, **B**), and α-2 adrenergic receptor (ADRA2A, **C**) expression in dopaminergic-, cholinergic-, and adrenergic-like neuronal cells, respectively. Data are shown as mean ± 95 % CI from N = 3-4 independent experiments, each performed in duplicate. Statistical significance was assessed by one-way ANOVA followed by Tukey’s post hoc test. Exact p-values: (**A**) Control vs RA, p = 0.356; Control vs RA+PMA, p = 0.002; RA vs RA+PMA, p = 0.009; (**B**) Control vs RA+BDNF, p = 0.036; (**C**) Control vs dbcAMP, p = 0.011. Data were obtained after 7 days of differentiation for dopaminergic- and cholinergic-like neuronal cells and after 4 days of differentiation for adrenergic-like cells and are expressed relative to IgG.

**
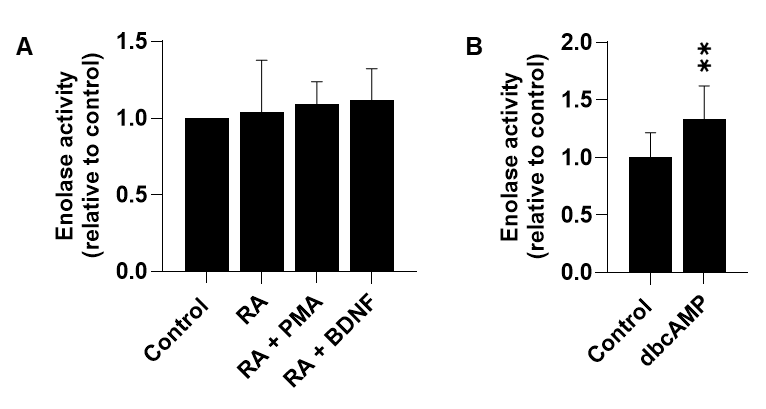
**

**Fig. S4.** **Enolase activity in SH-SY5Y cells differentiated into specific subtypes.** (**A, B**) The glycolytic activity of enolase in (**A**) dopaminergic- and cholinergic-like neuronal cells and (**B**) adrenergic-like neuronal cells. Data are shown as mean ± 95 % CI from N = 3-4 independent experiments, each performed in duplicate. Statistical significance was assessed by one-way ANOVA followed by Tukey’s post hoc test. Exact p-values: (**A**) one-way ANOVA not significant, p = 0.309; (**B**) Control vs dbcAMP, p = 0.005 Data were obtained after 7 days of differentiation for dopaminergic- and cholinergic-like neuronal cells and after 4 days of differentiation for adrenergic-like cells and are expressed relative to the control.

**
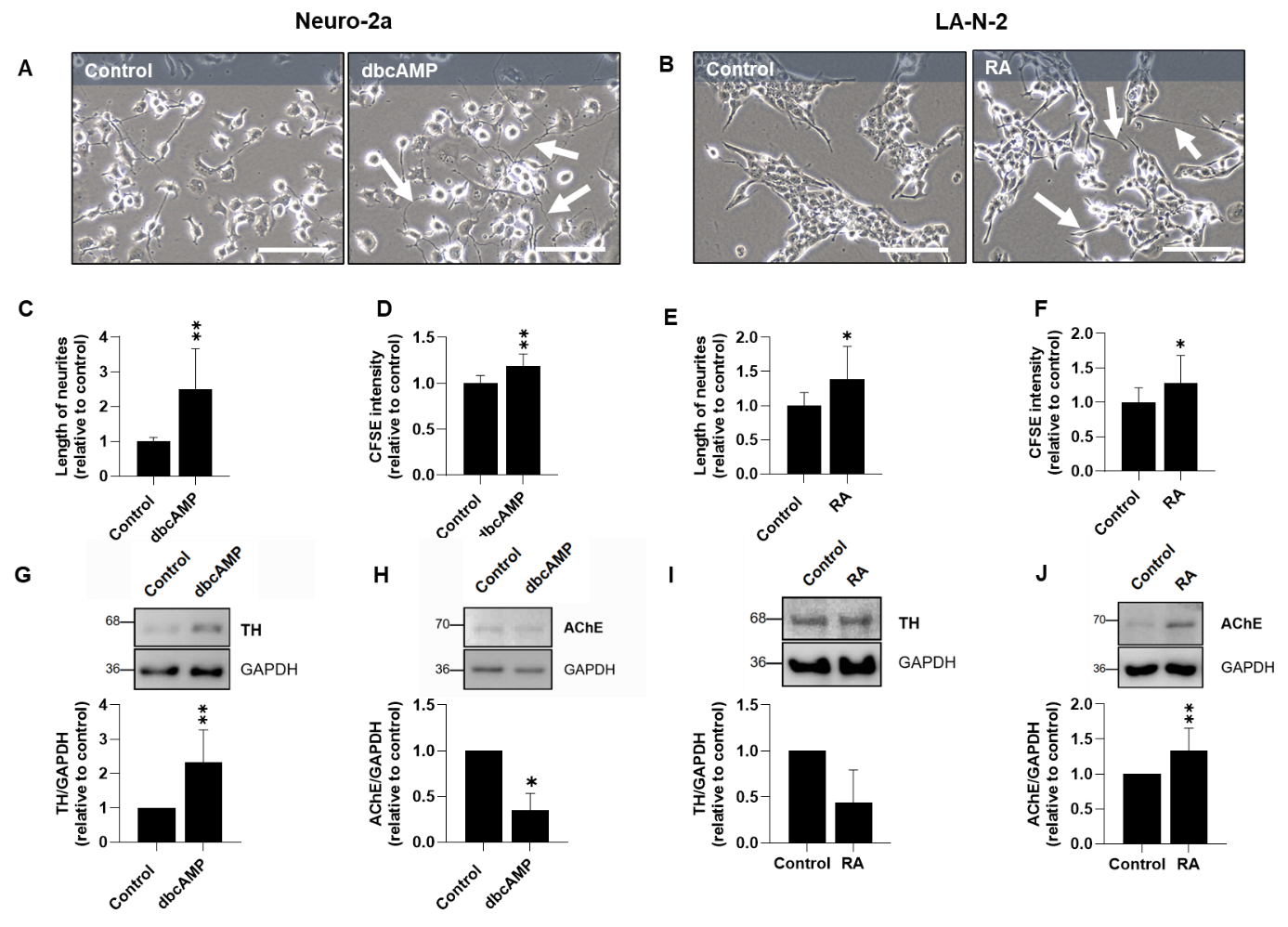
**

**Fig. S5. Differentiation of Neuro-2a and LA-N-2 cells into specific neuronal subtypes**. **(A, B**) Representative phase-contrast images of (**A**) Neuro-2a cells cultured in dibutyl cyclic AMP (dbcAMP, 0.5 mM) differentiation media and (**B**) LA-N-2 cells cultured in retinoic acid (RA) differentiation media. White arrows indicate cell extensions. Scale bars: 100 μm. (**C, E**) Neurite lengths in differentiated (**C**) Neuro-2a and (**E**) LA-N-2 cells were determined in pixels when the extensions were longer than the cell diameter using ImageJ software. Data are shown as mean ± 95 % CI from N = 3 independent experiments, each performed in duplicate. Statistical significance was assessed by one-way ANOVA followed by Tukey’s post hoc test. Exact p-values: (**C**) Control vs dbcAMP, p = 0.005; (**E**) Control vs RA, p = 0.034. (**D, F**) The proliferation rates of (**D**) Neuro-2a and (**F**) LA-N-2 cells were assessed with carboxyfluorescein succinimidyl ester (CFSE) labeling and flow cytometry. Data are shown as mean ± 95 % CI from N = 3 independent experiments, each performed in duplicate. Statistical significance was assessed by one-way ANOVA followed by Tukey’s post hoc test. Exact p-values: (**D**) Control vs dbcAMP, p = 0.009; (**F**) Control vs RA, p = 0.018. (**G–J**) Representative western blots (top) and quantification (bottom) of the expression of (**G, I**) tyrosine hydroxylase (TH) and (**H, J**) acetylcholinesterase (AChE) after differentiation of (**G, H**) Neuro-2a and (**I, J**) LA-N-2 cells. Protein levels are normalized to GAPDH. Data are shown as mean ± 95 % CI from N = 2 independent experiments. Statistical significance was assessed by one-way ANOVA followed by Tukey’s post hoc test. Exact p-values: (**G**) Control vs dbcAMP, p = 0.006; (**H**) Control vs dbcAMP, p = 0.014; (**I**) Control vs RA, p = 0.081; (**J**) Control vs RA, p = 0.004. Data were obtained after 4 days of differentiation.

**
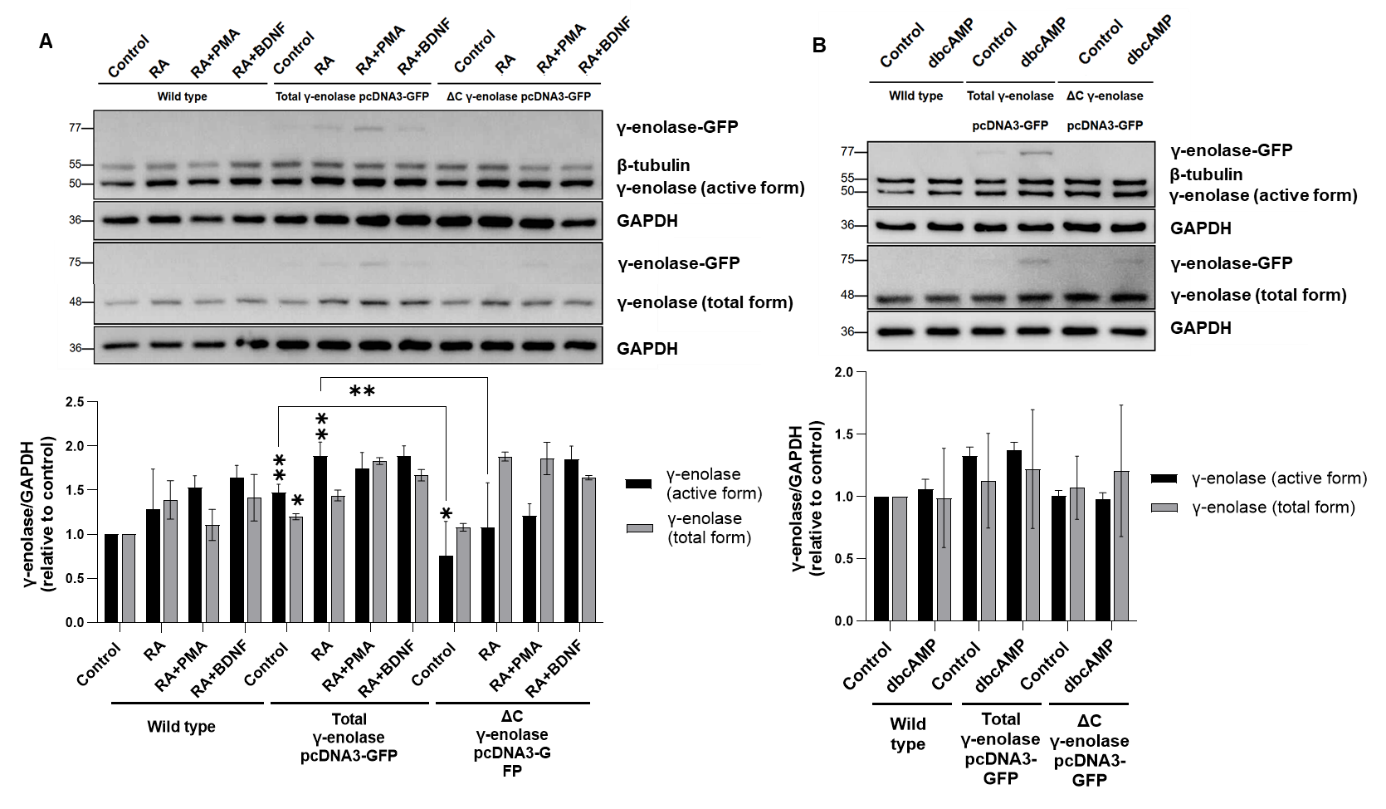
**

**Fig. S6. The efficiency of γ-enolase transfection in SH-SY5Y cells differentiated into specific subtypes.** (**A, B**) Representative western blots (top) and quantification (bottom) of the expression of the active and total form of γ-enolase in (**A**) dopaminergic-, cholinergic-, and (**B**) adrenergic-like neuronal cells. Protein levels are normalized to GAPDH. Data are shown as mean ± 95 % CI from N = 2-3 independent experiments. Statistical significance was assessed by one-way ANOVA followed by Tukey’s post hoc test. Exact p-values: (**A**) Control γ-enolase (active form) - Wild type vs Total γ-enolase pcDNA3-GFP, p = 0.001; Wild type vs ΔC γ-enolase pcDNA3-GFP, p = 0.024; Total γ-enolase pcDNA3-GFP vs ΔC γ-enolase pcDNA3-GFP, p = 0.001; Control γ-enolase (total form) - Wild type vs Total γ-enolase pcDNA3-GFP, p = 0.0246; Wild type vs ΔC γ-enolase pcDNA3-GFP, p = 0.225; Total γ-enolase pcDNA3-GFP vs ΔC γ-enolase pcDNA3-GFP, p = 0.091; RA γ-enolase (active form) - Wild type vs Total γ-enolase pcDNA3-GFP, p = 0.008; Wild type vs ΔC γ-enolase pcDNA3-GFP, p = 0.369; Total γ-enolase pcDNA3-GFP vs ΔC γ-enolase pcDNA3-GFP, p = 0.004; RA γ-enolase (total form) - not significant, p = 0.131; RA+PMA γ-enolase (active form) - not significant, p = 0.236; RA+PMA γ-enolase (total form) - Wild type vs Total γ-enolase pcDNA3-GFP, p = 0.065; Wild type vs ΔC γ-enolase pcDNA3-GFP, p = 0.059; Total γ-enolase pcDNA3-GFP vs ΔC γ-enolase pcDNA3-GFP, p = 0.890; RA+BDNF γ-enolase (active form) - not significant, p = 0.753; RA+BDNF γ-enolase (total form) - not significant, p = 0.787; (**B**) Control γ-enolase (active form) - not significant, p = 0.361; Control γ-enolase (total form) - not significant, p = 0.408; dbcAMP γ-enolase (active form) - not significant, p = 0.082; dbcAMP γ-enolase (total form) - not significant, p = 0.465. Data were obtained after 7 days of differentiation for dopaminergic- and cholinergic-like neuronal cells and after 4 days of differentiation for adrenergic-like neuronal cells.

**
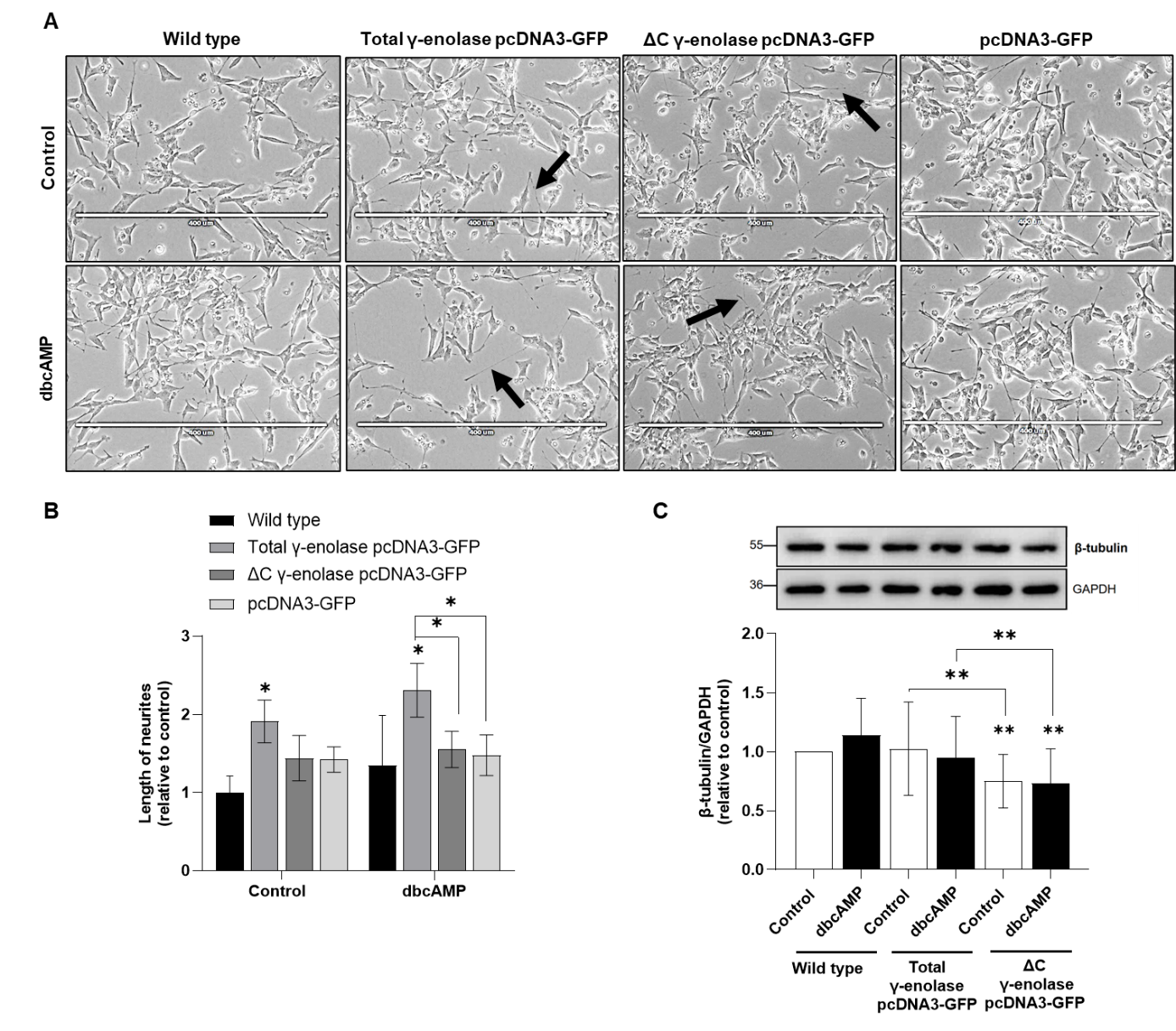
**

**Fig. S7. Morphological changes and expression of β-tubulin after γ-enolase upregulation in adrenergic-like SH-SY5Y cells.** (**A**) Representative phase-contrast images of SH-SY5Y cells transfected with the ΔC γ-enolase pcDNA3-GFP and total γ-enolase pcDNA3-GFP plasmids. Black arrows indicate cell extensions. Scale bars: 400 μm. (**B**) Neurite lengths were determined in pixels using ImageJ software when the extensions were longer than the cell diameter. Data are expressed relative to the control. Data are shown as mean ± 95 % CI from N = 2 independent experiments. Statistical significance was assessed by one-way ANOVA followed by Tukey’s post hoc test. Exact p-values: Control - Wild type vs Total γ-enolase pcDNA3-GFP, p = 0.023; Wild type vs ΔC γ-enolase pcDNA3-GFP, p = 0.173; Wild type vs pcDNA3-GFP, p = 0.217; Total γ-enolase pcDNA3-GFP vs ΔC γ-enolase pcDNA3-GFP, p = 0.206; Total γ-enolase pcDNA3-GFP vs pcDNA3-GFP, p = 0.165; ΔC γ-enolase pcDNA3-GFP vs pcDNA3-GFP, p = 0.900; dbcAMP - Wild type vs Total γ-enolase pcDNA3-GFP, p = 0.015; Wild type vs ΔC γ-enolase pcDNA3-GFP, p = 0.538; Wild type vs pcDNA3-GFP, p = 0.836; Total γ-enolase pcDNA3-GFP vs ΔC γ-enolase pcDNA3-GFP, p = 0.039; Total γ-enolase pcDNA3-GFP vs pcDNA3-GFP, p = 0.025; ΔC γ-enolase pcDNA3-GFP vs pcDNA3-GFP, p = 0.900. (**C**) Representative western blots (top) and quantification (bottom) of the expression of β-tubulin. Protein levels are normalized to GAPDH and expressed relative to the control (wild type). Data are shown as mean ± 95 % CI from N = 2-3 independent experiments. Statistical significance was assessed by one-way ANOVA followed by Tukey’s post hoc test. Exact p-values: Control - Wild type vs Total γ-enolase pcDNA3-GFP, p = 0.691; Wild type vs ΔC γ-enolase pcDNA3-GFP, p = 0.018; Total γ-enolase pcDNA3-GFP vs ΔC γ-enolase pcDNA3-GFP, p = 0.012; dbcAMP - Wild type vs Total γ-enolase pcDNA3-GFP, p = 0.123; Wild type vs ΔC γ-enolase pcDNA3-GFP, p = 0.012; Total γ-enolase pcDNA3-GFP vs ΔC γ-enolase pcDNA3-GFP, p = 0.107. Data were obtained after 4 days of differentiation.


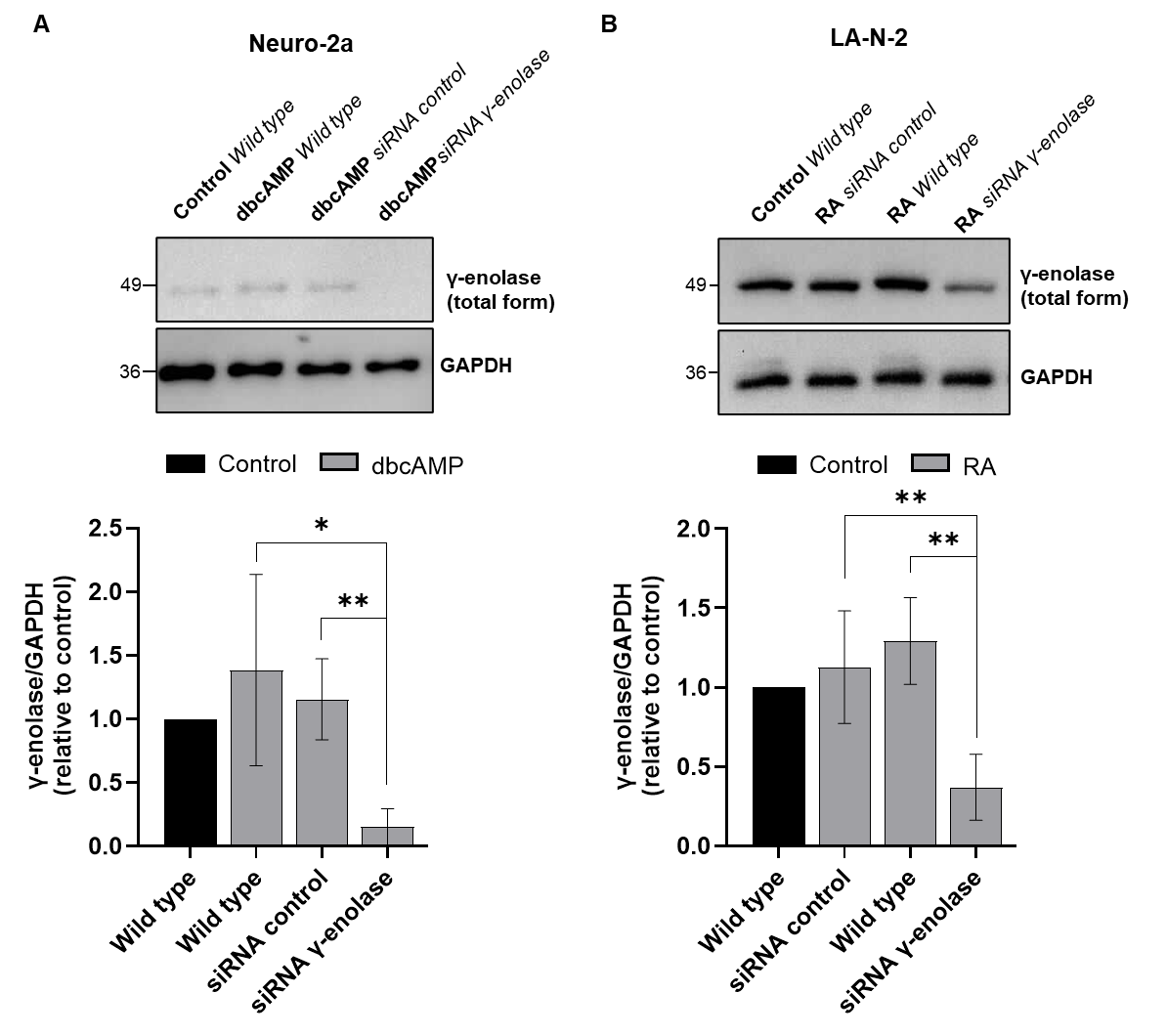


**Fig. S8. Effect of γ-enolase silencing in differentiated Neuro-2a and LA-N-2 cells into specific neuronal subtypes.** (**A, B**) Representative western blots (top) and quantification (bottom) of the expression of γ-enolase after silencing and differentiation of Neuro-2a (**A**) and LA-N-2 **(B**) cells. Protein levels are normalized to GAPDH. Data are shown as mean ± 95 % CI from N = 2-3 independent experiments. Statistical significance was assessed by one-way ANOVA followed by Tukey’s post hoc test. Exact p-values: (**A**) Control Wild type vs dbcAMP Wild type, p = 0.175; dbcAMP Wild type vs dbcAMP siRNA control, p = 0.481; dbcAMP Wild type vs dbcAMP siRNA γ-enolase, p = 0.034; dbcAMP siRNA control vs dbcAMP siRNA γ-enolase, p = 0.008; (**B**) Control Wild type vs RA Wild type, p = 0.111; RA Wild type vs RA siRNA control, p = 0.405; RA Wild type vs RA siRNA γ-enolase, p = 0.002; RA siRNA control vs RA siRNA γ-enolase, p = 0.004. Data were obtained after 4 days of differentiation.


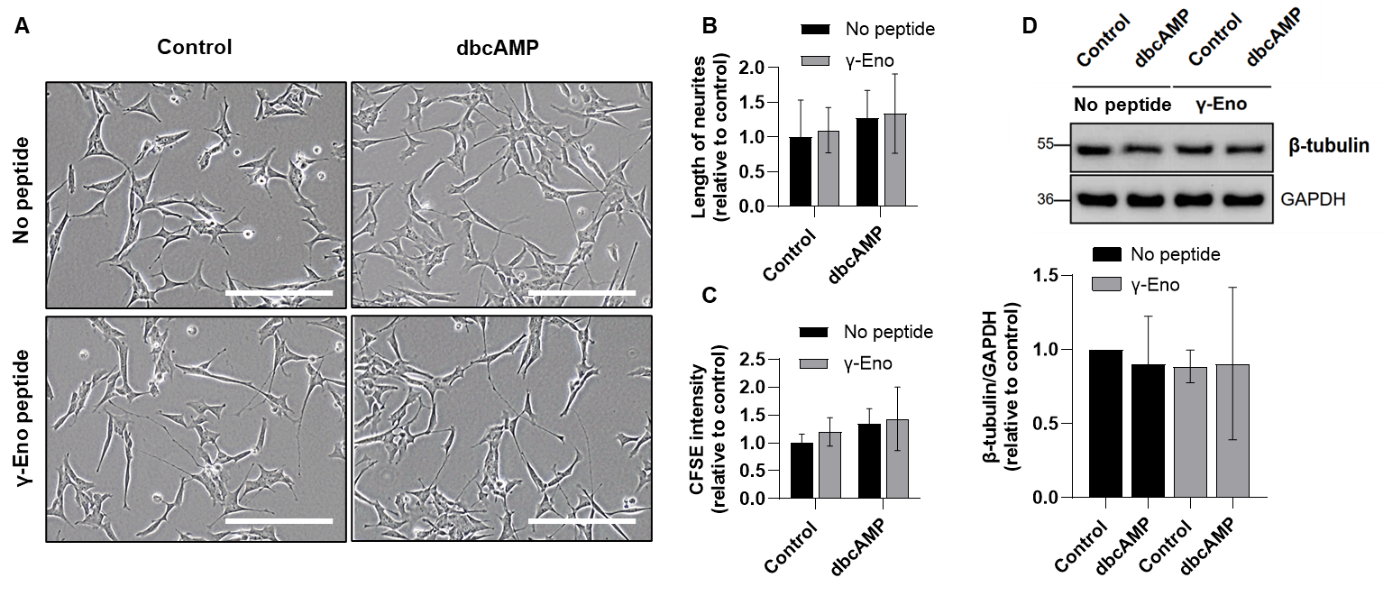


**Fig. S9. The effects of γ-enolase peptide treatment on the morphology, proliferation, and expression of β-tubulin in adrenergic-like SH-SY5Y cells.** (**A**) Representative phase-contrast images of SH-SY5Y cells treated with γ-enolase peptide corresponding to the last 30 amino acids of the protein (γ-Eno). Scale bars: 100 μm. (**B**) Neurite lengths in adrenergic-like neuronal cells treated with γ-Eno were determined in pixels when the extensions were longer than the cell diameter using ImageJ software. Data are shown as mean ± 95 % CI from N = 2 independent experiments, each performed in duplicate. Statistical significance was assessed by one-way ANOVA. Exact p-value: not significant, p = 0.112. (**C**) The proliferation rates of adrenergic-like neuronal cells treated with γ-Eno were assessed with carboxyfluorescein succinimidyl ester (CFSE) labeling and flow cytometry. Data are shown as mean ± 95 % CI from N = 4 independent experiments, each performed in duplicate. Statistical significance was assessed by one-way ANOVA. Exact p-value: not significant, p = 0.200. (**D**) Representative western blots (top) and quantification (bottom) of the expression of β-tubulin in adrenergic-like neuronal cells. Protein levels are normalized to GAPDH and expressed relative to the control. Data are shown as mean ± 95 % CI from N = 3 independent experiments. Statistical significance was assessed by one-way ANOVA. Exact p-value: not significant p = 0.052. Data were obtained after 4 days of differentiation.

**
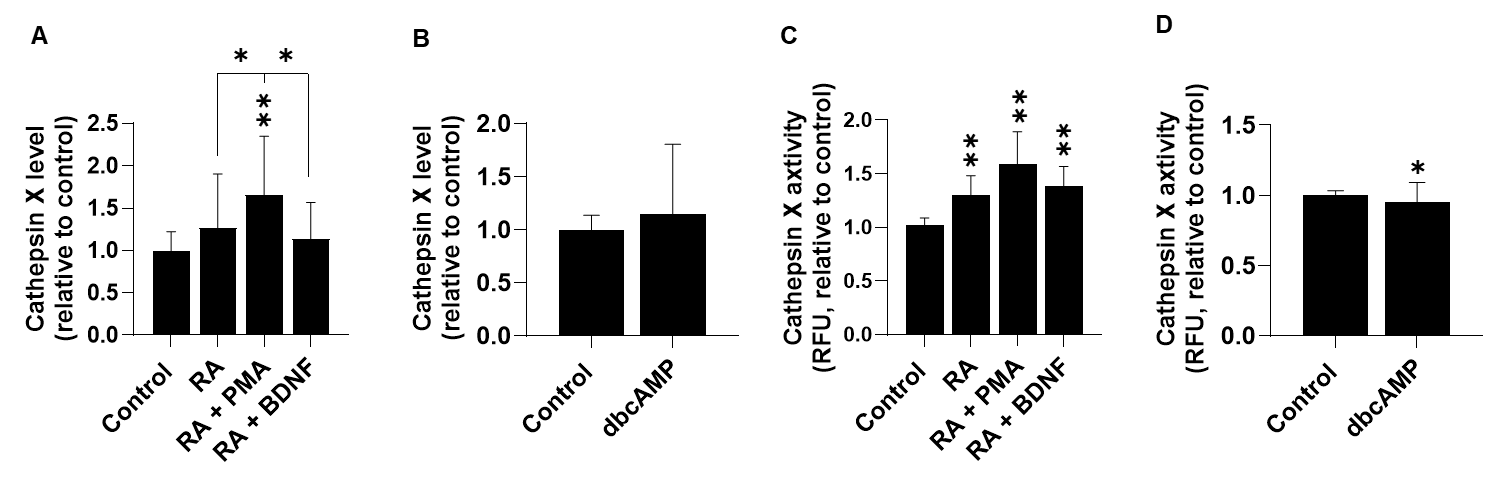
**

**Fig. S10. Cathepsin X expression and activity in SH-SY5Y cells differentiated into specific subtypes.** (**A, B**) ELISA analysis of cathepsin X expression in SH-SY5Y cells differentiated into (**A**) dopaminergic-, cholinergic-, and (**B**) adrenergic-like neuronal cells. Data are shown as mean ± 95 % CI from N = 2 independent experiments, each performed in duplicate. Statistical significance was assessed by one-way ANOVA followed by Tukey’s post hoc test. Exact p-values: (**A**) Control vs RA, p = 0.900; Control vs. RA+PMA, p = 0.001; Control vs RA+BDNF, p = 0.512; RA vs RA+PMA, p = 0.001; RA vs RA+BDNF, p = 0.830; RA+PMA vs RA+BDNF, p = 0.002; (**B**) Control vs dbcAMP, p = 0.398. (**C, D**) Cathepsin X activity was measured using the specific substrate Abz-Phe-Glu-Lys(Dnp)-OH in (**C**) dopaminergic-, cholinergic-, and (**D**) adrenergic-like neuronal cells. Data are shown as mean ± 95 % CI from N = 5-7 independent experiments, each performed in duplicate. Statistical significance was assessed by one-way ANOVA followed by Tukey’s post hoc test. Exact p-values: (**C**) Control vs RA, p = 0.003; Control vs. RA+PMA, p = 0.001; Control vs RA+BDNF, p = 0.001; RA vs RA+PMA, p = 0.055; RA vs RA+PMA, p = 0.634; RA+PMA vs RA+BDNF, p = 0.434; (**D**) Control vs dbcAMP, p = 0.032. Activity was assessed after 7 days of differentiation for dopaminergic- and cholinergic-like neuronal cells and 4 days of differentiation for adrenergic-like cells. Data are expressed relative to the control.

**
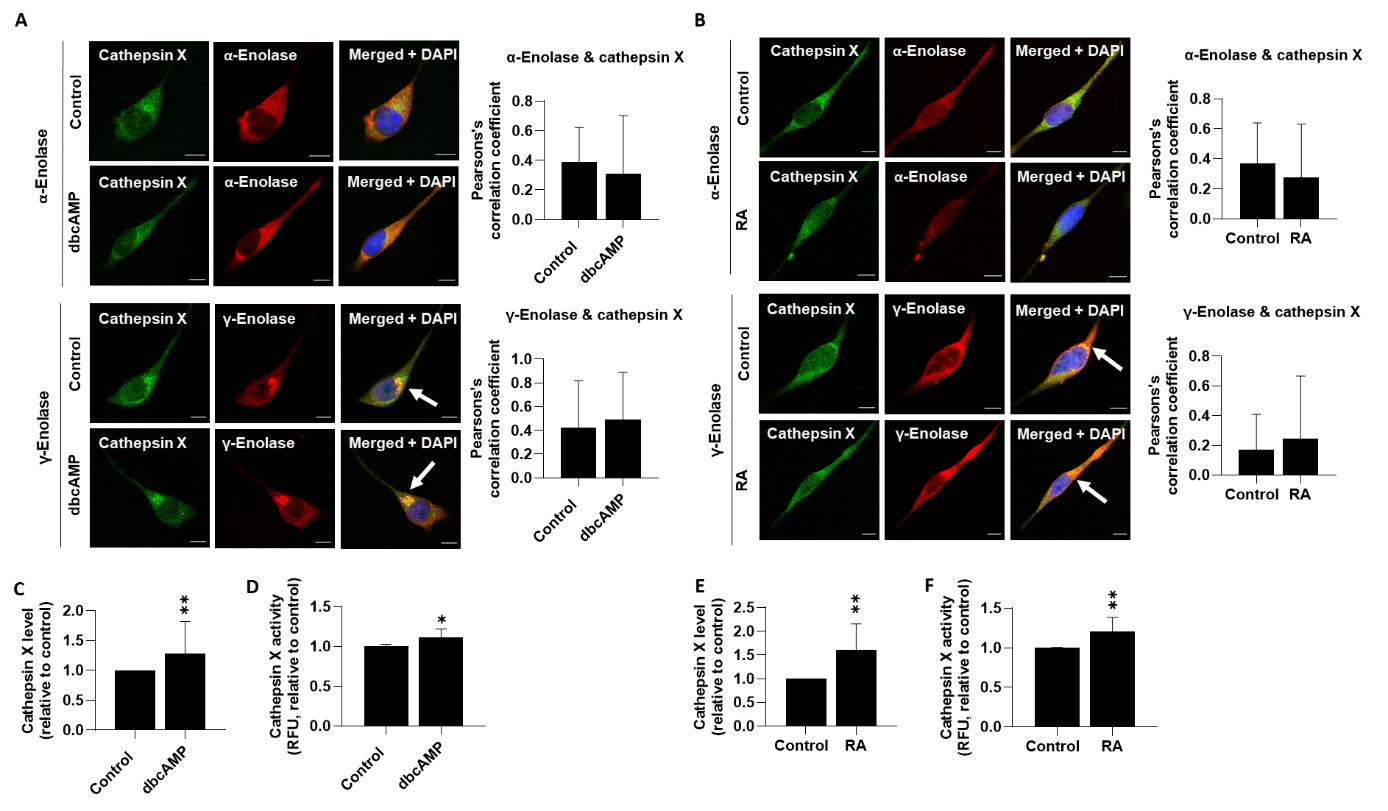
**

**Fig. S11. Cathepsin X co-localization, expression, and activity in Neuro-2a and LA-N-2 cells differentiated into specific neuronal subtypes.** (**A, B**) Representative images of immunofluorescence staining for cathepsin X (green) and α-enolase (red, top) or γ-enolase (red, bottom) for differentiated (**A**) Neuro-2a and (**B**) LA-N-2 cells. Nuclei were counterstained with DAPI (blue). White arrows indicate areas with strong co-localization. Scale bars: 10 μm. The graphs (right) show Pearson’s correlation coefficients. Data are shown as mean ± 95 % CI from N = 2 independent experiments. Statistical significance was assessed by one-way ANOVA. Exact p-values: (**A**) α-enolase & cathepsin X - Control vs dbcAMP, p = 0.648; γ-enolase & cathepsin X - Control vs dbcAMP , p = 0.070; (**B**) α-enolase & cathepsin X Control vs RA, p = 0.095; γ-enolase & cathepsin X - Control vs RA , p = 0.254. (**C, E**) ELISA analysis of cathepsin X expression in differentiated (**C**) Neuro-2a and (**E**) LA-N-2 cells. Data are shown as mean ± 95 % CI from N = 2 independent experiments, each in duplicate. Statistical significance was assessed by one-way ANOVA. Exact p-values: (**C**) Control vs dbcAMP , p = 0.021; (**E**) Control vs RA, p = 0.019. (**D, F**) Cathepsin X activity based on cathepsin X-specific substrate (Abz-Phe-Glu-Lys(Dnp)-OH) in differentiated (**D**) Neuro-2a and (**F**) LA-N-2 cells. Data are shown as mean ± 95 % CI from N = 4-6 independent experiments, each performed in duplicate. Statistical significance was assessed by one-way ANOVA. Exact p-values: (**D**) Control vs dbcAMP, p = 0.035; (**F**) Control vs RA, p = 0.010. Data were obtained after 4 days of differentiation and are expressed relative to the control.

**
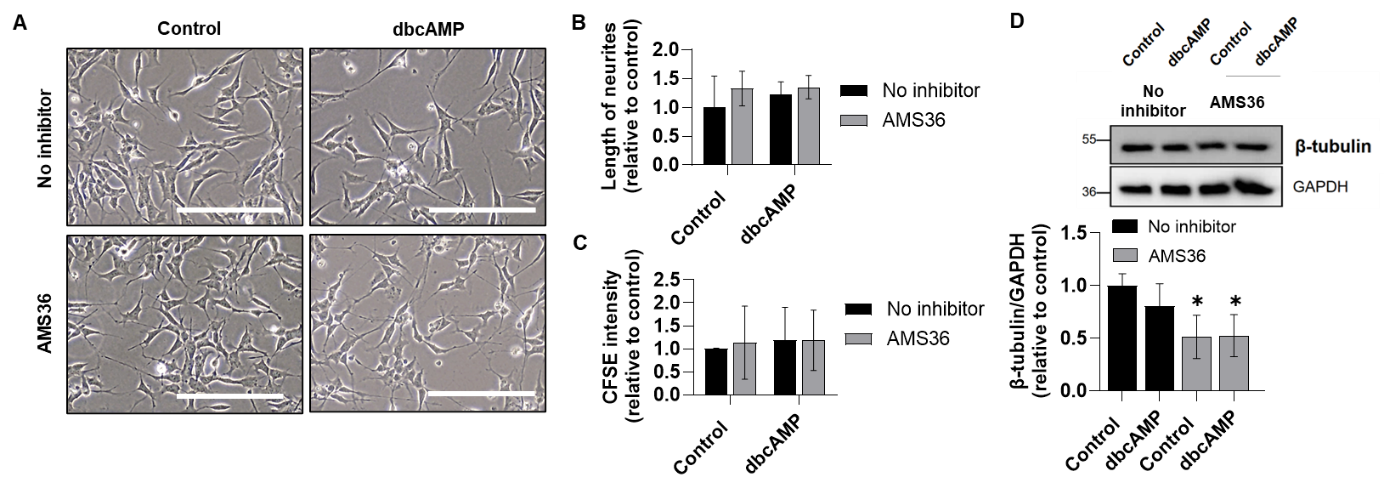
**

**Fig. S12. The effects of cathepsin X inhibition on cell morphology and the expression of γ-enolase, β-tubulin in adrenergic-like SH-SY5Y cells.** (**A**) Representative phase-contrast images of SH-SY5Y cells treated with AMS36. Scale bars: 100 μm. (**B**) Neurite lengths were determined in pixels using ImageJ software when cell extensions were longer than the cell diameter. Data are shown as mean ± 95 % CI from N = 2 independent experiments, each performed in duplicate. Statistical significance was assessed by one-way ANOVA. Exact p-value: not significant, p = 0.075. (**C**) The proliferation rates of AMS36-treated adrenergic-like neuronal cells were assessed with carboxyfluorescein succinimidyl ester (CFSE) labeling and flow cytometry. Data are shown as mean ± 95 % CI from N = 4 independent experiments, each performed in duplicate. Statistical significance was assessed by one-way ANOVA. Exact p-value: not significant, p = 0.200. **(D)** Representative western blots (top) and quantification (bottom) of the expression of β-tubulin. Protein levels are normalized to GAPDH and expressed relative to the control. Data are shown as mean ± 95 % CI from N = 2 independent experiments. Statistical significance was assessed by one-way ANOVA followed by Tukey’s post hoc test. Exact p-values: Control / No inhibitor vs dbcAMP / No inhibitor, p = 0.322; Control / No inhibitor vs Control / AMS36, p = 0.022; Control / No inhibitor vs dbcAMP / AMS36, p = 0.032; dbcAMP / No inhibitor vs dbcAMP / AMS36, p = 0.900. Data were obtained after 4 days of differentiation and are expressed relative to the control.

**
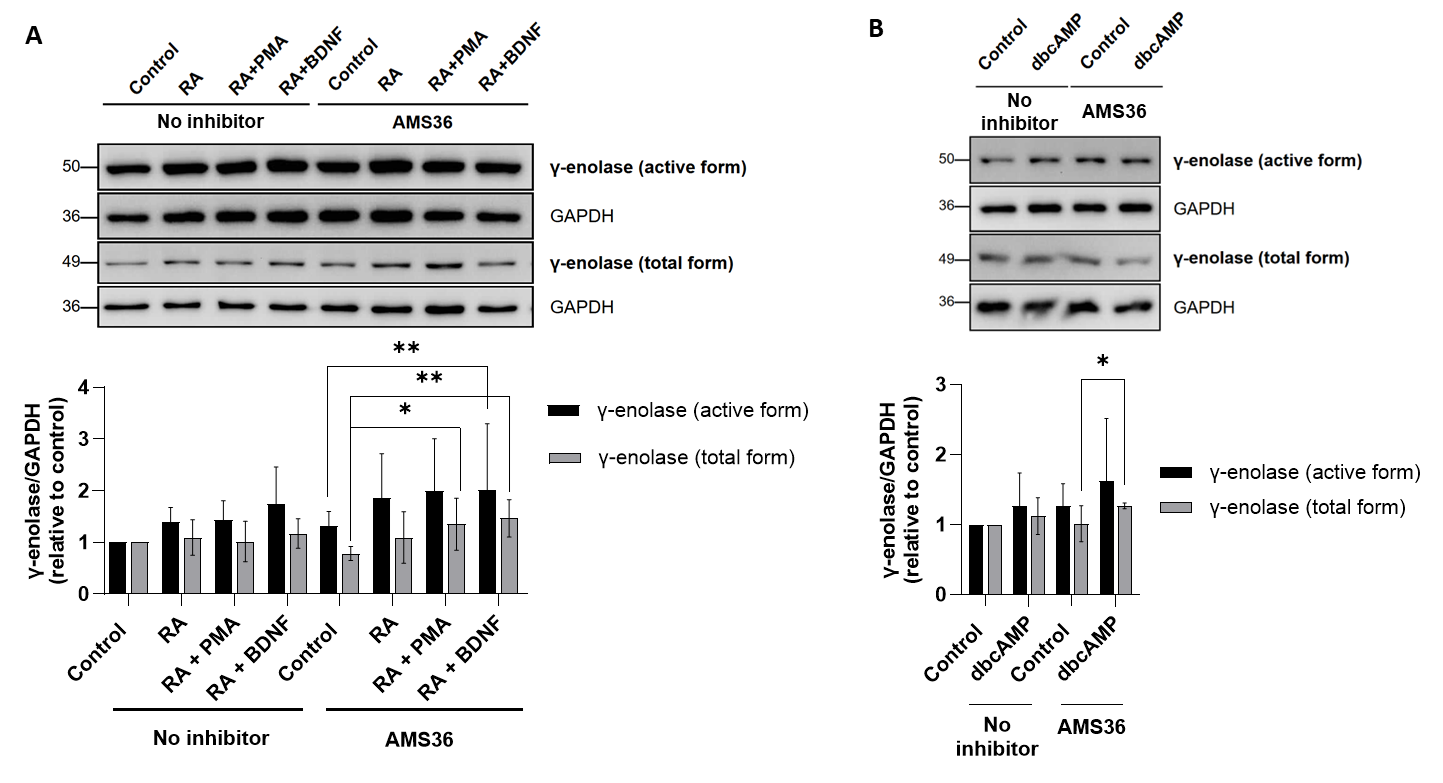
**

**Fig. S13. The effects of cathepsin X inhibition on γ-enolase expression in differentiated SH-SY5Y cells. (A, B)** Representative western blots (top) and quantification (bottom) of the active and total forms of γ-enolase in (**A**) dopaminergic-, cholinergic- and (**B**) adrenergic-like cells neuronal cells. Protein levels are normalized to GAPDH and expressed relative to the control. Data are shown as mean ± 95 % CI from N = 3-4 independent experiments. Statistical significance was assessed by one-way ANOVA followed by Tukey’s post hoc test. Exact p-values: (**A**) Control γ-enolase (active form) - No inhibitor vs AMS36, p = 0.856; RA γ-enolase (active form) - No inhibitor vs AMS36, p = 0.430; RA+PMA γ-enolase (active form) - No inhibitor vs AMS36, p = 0.657; RA+BDNF γ-enolase (active form) - No inhibitor vs AMS36, p = 0.300; AMS36 γ-enolase (active form) – Control vs RA, p = 0.4345; AMS36 γ-enolase (active form) – Control vs RA+PMA, p = 0.319; AMS36 γ-enolase (active form) – Control vs RA+BDNF, p = 0.009; Control γ-enolase (total form) - No inhibitor vs AMS36, p = 0.851; RA γ-enolase (total form) - No inhibitor vs AMS36, p = 0.900; RA+PMA γ-enolase (total form) - No inhibitor vs AMS36, p = 0.518; RA+BDNF γ-enolase (total form) - No inhibitor vs AMS36, p = 0.900; AMS36 γ-enolase (total form) – Control vs RA, p = 0.531; AMS36 γ-enolase (total form) – Control vs RA+PMA, p = 0.030; AMS36 γ-enolase (total form) – Control vs RA+BDNF, p = 0.006; (**B**) γ-enolase (active form), not significant, p = 0.368; Control γ-enolase (total form) - No inhibitor vs AMS36, p = 0.900; dbcAMP γ-enolase (total form) - No inhibitor vs AMS36, p = 0.269; AMS36 γ-enolase (total form) – Control vs dbcAMP, p = 0.037. Data were obtained after 7 days of differentiation for dopaminergic- and cholinergic-like neuronal cells and after 4 days of differentiation for adrenergic-like neuronal cells and are expressed relative to the control.
